# Gene Structures, Evolution and Transcriptional Profiling of the *WRKY* Gene Family in Castor Bean (*Ricinus communis* L.)

**DOI:** 10.1371/journal.pone.0148243

**Published:** 2016-02-05

**Authors:** Zhi Zou, Lifu Yang, Danhua Wang, Qixing Huang, Yeyong Mo, Guishui Xie

**Affiliations:** 1 Danzhou Investigation & Experiment Station of Tropical Crops, Ministry of Agriculture, Rubber Research Institute, Chinese Academy of Tropical Agricultural Sciences, Danzhou, Hainan, P. R. China; 2 Institute of Tropical Biosciences and Biotechnology, Chinese Academy of Tropical Agricultural Sciences, Haikou, Hainan, P. R. China; Jawaharlal Nehru University, INDIA

## Abstract

*WRKY* proteins comprise one of the largest transcription factor families in plants and form key regulators of many plant processes. This study presents the characterization of 58 *WRKY* genes from the castor bean (*Ricinus communis* L., Euphorbiaceae) genome. Compared with the automatic genome annotation, one more *WRKY*-encoding locus was identified and 20 out of the 57 predicted gene models were manually corrected. All *RcWRKY* genes were shown to contain at least one intron in their coding sequences. According to the structural features of the present *WRKY* domains, the identified *RcWRKY* genes were assigned to three previously defined groups (I–III). Although castor bean underwent no recent whole-genome duplication event like physic nut (*Jatropha curcas* L., Euphorbiaceae), comparative genomics analysis indicated that one gene loss, one intron loss and one recent proximal duplication occurred in the *RcWRKY* gene family. The expression of all 58 *RcWRKY* genes was supported by ESTs and/or RNA sequencing reads derived from roots, leaves, flowers, seeds and endosperms. Further global expression profiles with RNA sequencing data revealed diverse expression patterns among various tissues. Results obtained from this study not only provide valuable information for future functional analysis and utilization of the castor bean *WRKY* genes, but also provide a useful reference to investigate the gene family expansion and evolution in Euphorbiaceus plants.

## Introduction

WRKY transcription factors, defined by the presence of the conserved WRKY domain of approximate 60 amino acids, play an essential regulatory role in plant growth, development, metabolism, and biotic and abiotic stress responses [1–3]. Since the first WRKY-encoding gene was isolated from sweet potato (*Ipomoea batatas*) [4], its homologs have been found in a wide range of plants and several non-plant species including *Giardia lamblia*, *Dictyostelium discoideum*, diplomonads, social amoebae, fungi *incertae sedis* and amoebozoa [5,6]. Compared with low and non-plants, the *WRKY* genes in high plants were shown to be highly expanded. For example, there are 57 members in cucumber (*Cucumis sativus*), 58 in physic nut (*Jatropha curcas*), 59 in grapevine (*Vitis vinifera*), 72 in *Arabidopsis thaliana*, 103 in white pear (*Pyrus bretschneideri*), 105 in poplar (*Populus trichocarpa*), 105 in foxtail millet (*Setaria italica*) and more than 100 in rice (*Oryza sativa*) [7–14]. WRKY proteins contain one or two WRKY domains, comprising the highly conserved WRKYGQK heptapeptide at the N-termini and a novel zinc finger motif (Cx_4–7_Cx_22–23_HxH/C) at the C-termini [10]. Both of these two motifs are vital for the high binding affinity of the WRKY proteins to the consensus cis-acting element termed the W box (TTGACT/C) [15,16]. According to the number of WRKY domains and the features of their zinc finger motifs, WRKY proteins can be categorized into three main groups. The group I members have two WRKY domains and feature the zinc finger motif of C_2_H_2_. Both groups II and III members contain a single WRKY domain, and the group III members possess the C_2_HC zinc finger motif which is different from C_2_H_2_ as observed in groups I and II members. Base on the evolutionary relationship and certain amino acid motifs present outside the WRKY domain, the group II can be further divided into 5 subgroups (a–e) [10]. In contrast to the presence of a conserved PR intron located after the codon encoding arginine (N terminal to the zinc finger motif) of subgroups c-e as seen in the group III and the C-terminal WRKY domain of the group I, members of subgroups a and b harbor a VQR intron in the zinc finger motif instead [6,10,17].

Castor bean (*Ricinus communis* L.), a tropical perennial shrub that belongs to the Euphorbiaceae family, is one of the most important non-food oilseed crops cultivated for industrial, medicinal and cosmetic purposes. Although native to Africa, the economic importance of castor bean oil and its well-adaptation to unfavorable conditions has prompted its wide-domestication to many tropical, subtropical and warm temperate regions around the world [18,19]. Given the crucial role of WRKY transcription factors in plant adaptation, two independent groups performed the homology search against the recently available castor bean draft genome [20] for the *RcWRKY* genes [17,21]. The study performed by Li et al. [21] focused on the expression analysis of the 47 identified *RcWRKY* genes in roots, stems, leaves, male flowers, female flowers and fruits at different developmental stages (i.e. 7, 15, 30 and 45 days post-anthesis) by using quantitative real-time PCR (qRT-PCR). Another study carried out by Zou [17] described the identification of nine more family members (i.e. 56 *RcWRKYs*) based on the automatic annotation of the castor bean genome, mainly focusing on the analysis of the evolutionary relationships between RcWRKY members by using the conserved WRKY domains. However, when compared with physic nut, another Euphorbiaceae plant species without the occurrence of any recent whole-genome duplication as castor bean [20,22], the family number of castor bean [17] seems to be relatively small and several physic nut *WRKY* genes [7] have no counterparts in castor bean. These results suggest that the *RcWRKY* genes have not been fully identified or the loss of specific genes has occurred in the castor bean genome. Thereby, rechecking the *RcWRKY* gene family is still needed.

Along with the 4.6 × draft genome of castor bean, as of Apr 2015, 88212 nucleotides and 62629 expressed sequence tags (ESTs) have been deposited in NCBI GenBank. In addition, RNA sequencing data from several tissues such as root, leaf, flower, seed and endosperm is also available in NCBI SRA, which includes 1,138,884 Roche 454 reads and 386,847,526 Illumina reads [23–26]. These datasets provide a good chance to analyze the castor bean *WRKY* gene family from a global view. In the present study, we take advantage of the genome sequences and available transcriptome data to identify the complete set of the *RcWRKY* genes and conduct the expert revision of their gene structures via mapping the ESTs and RNA sequencing reads against the scaffolds. Further, the sequence characteristics, evolutionary relationships and transcriptional profiling of the identified *RcWRKY* genes were also investigated.

## Methods

### Datasets and sequence retrieval

Sequences of 72 *Arabidopsis* and 58 physic nut WRKY proteins described before [7,10] were obtained from TAIR (release 10, http://www.arabidopsis.org/) and NCBI (http://www.ncbi.nlm.nih.gov/), respectively (the accession number are available in S1 Table). The genome sequences and annotation information of castor bean [20] were downloaded from phytozome v10.2 (http://phytozome.jgi.doe.gov/pz/portal.html), whereas the nucleotides, Sanger ESTs and raw RNA sequencing reads were downloaded from NCBI.

### Identification and manual curation of the castor bean *WRKY* genes

To obtain the complete set of castor bean *WRKY* genes, the tBlastn search [27] was performed using a representative WRKY domain from each WRKY subgroups (I, IIa, IIb, IIc, IId, IIe and III) and the e-value was set to 10. Positive genomic sequences were also analyzed using the HMMER program [28] and Hidden Markov Model (HMM) trained with RcWRKYs. The presence of WRKY domains in candidate RcWRKY proteins was confirmed using the SMART program (http://smart.embl-heidelberg.de/) [29]. The predicted gene models were further checked with ESTs and raw RNA sequencing reads. Gene structures were displayed using GSDS [30]. Homology search for nucleotides or ESTs was performed using Blastn [27] and sequences with a similarity of more than 98% were taken into account, whereas RNA sequencing clean reads (see below) were mapped using Bowtie 2 [31] with default parameters and mapped read number of more than one was counted as expressed. The alternative splicing isoforms were identified using Cufflinks (v2.2.1) [32]. In addition, the ortholog of each RcWRKY in *Arabidopsis* and physic nut was identified using Blastp [27] (e-value, 1e−20) against AtWRKYs and JcWRKYs, and the reciprocal Blastp was performed to confirm true orthologs. Tandem or proximal duplications were considered when two duplicated genes were consecutive in the genome or separated by 20 or fewer gene loci, respectively.

### Sequence alignments, phylogenetic analysis and classification of *RcWRKY* genes

Multiple alignments were performed using MUSCLE [33]. The alignment of all RcWRKY domains were displayed using Boxshade (http://www.ch.embnet.org/software/BOX_form.html), whereas the alignment including *Dictyostelium discoideum* WRKY1 [5] (UniProtKB accession number Q554C5; the N and C-terminal WRKY domain was denoted as DdWRKY1N or DdWRKY1C, respectively; the same as for other group I members), RcWRKYs, AtWRKYs and JcWRKYs were used for phylogenetic tree construction. By using DdWRKY1C as an outgroup, the tree was constructed using MEGA 6.0 [34] with the maximum likelihood method and with the bootstrap test replicated 1000 times. Classification of RcWRKYs into groups and subgroups was done based on the structural features and evolutionary relationships of the WRKY domains.

### Protein properties and conserved motif analysis

Protein properties of RcWRKYs, e.g., the molecular weight (MW), isoelectric point (*p*I), and grand average of hydropathicity (GRAVY) were calculated using ProtParam (http://web.expasy.org/protparam/). Analysis for conserved motifs in RcWRKY proteins was carried out using MEME (http://meme.sdsc.edu/meme/cgi-bin/meme.cgi) [35]. The optimized parameters were: any number of repetitions; maximum number of motifs, 15; and the optimum width of each motif, between 6 and 50 residues. Subsequently, the MAST program was used to search detected motifs in protein databases. The online software 2ZIP (http://2zip.molgen.mpg.de/index.html) was used to predict the conserved Leu zipper motif, whereas HARF, LxxLL (x, any amino acid) and LxLxLx motifs were identified manually.

### Gene expression analyses

To analyze the global expression profiles of *RcWRKY* genes among different tissues or certain tissue of developmental stages, RNA sequencing data of leaf (NCBI SRA accession number ERX021378), flower (ERX021379), endosperm (ERX021375 and ERX021376) and seed (ERX021377) described before [24] were examined. The clean reads were obtained by removing adaptor sequences, adaptor-only reads, reads with “N” rate larger than 10% (“N” representing ambiguous bases) and low quality reads containing more than 50% bases with Q-value≤5. Then, the clean reads were mapped to the 58 identified *RcWRKY* genes (coding sequence, CDS) and released transcripts using Bowtie 2 [31], and the RPKM (reads per kilo bases per million reads) method [36] was used for the expression annotation. Unless specific statements, the tools used in this study were performed with default parameters.

## Results and Discussion

### Characterization of 58 *WRKY*-encoding sequences in castor bean

The homology search resulted in 58 loci putatively encoding *WRKY* genes from 41 scaffolds of the castor bean genome. Among them, 57 loci were predicted by the genome annotation [20] and further annotated by the PlantTFDB which used the released gene models for the annotation of *RcWRKY* genes [37], whereas one more loci encoding 117 residues was identified from the scaffold28842 (Table 1) and its ortholog was also found in physic nut [7]. Since the gene models of *RcWRKY* genes were the result of an automatic annotation due to the lack of transcriptome data at that time, an expert revision of their gene structures was conducted via mapping the ESTs and reads against the scaffolds. Interestingly enough, the results showed that 20 out of the 57 predicted gene models seem not to be properly annotated (Table 1). The locus 29929.t000090 was predicted to encode 609 residues which is relatively shorter than its ortholog in physic nut (JcWRKY10, 740 residues) [7], however, hundreds of RNA sequencing reads indicated that the “TTNNNTTGAC” sequence was misassembled into its first exon. Thereby, this locus is promised to harbor four introns putatively encoding 711 residues (see S1 File). The locus 29820.t000050 was predicted to encode 558 residues, however, read mapping indicated that partial sequences of its second and third exons were annotated as the second intron, thus this locus is promised to encode 598 residues (see S2 File) which is similar to that of its physic nut ortholog (JcWRKY08, 576 residues) [7]. As for the locus 29635.t000028, though both the predicted and identified CDSs encode 510 residues, read mapping indicated that the “GCAA” sequence of the second intron was annotated as the second exon and the “GCAG” sequence of the third exon was annotated as the second intron (see S3 File). The locus 30174.t000563 was predicted to encode 468 residues, however, read mapping and ORF (open reading frame) analysis suggested that it represents only the 3’ sequence of the gene which is promised to encode 524 residues (see S4 File). The locus 29687.t000003 was predicted to contain five introns encoding 503 residues, however, sequence analysis indicated that the N-terminal WRKY domain of the deduced protein is incomplete. EST and read mapping suggested that this locus is promised to harbor four introns and putatively encode 511 residues (see S5 File). The locus 29848.t000095 was predicted to have two introns encoding 372 residues, however, read mapping indicated that it represents only the 3’ sequence of this gene which is promised to harbor four introns putatively encoding 451 residues. In addition, its third exon was also misannotated as an intron (see S6 File). The locus 30174.t000066 was predicted to encode 192 residues, however, read mapping indicated that this locus is promised to encode 196 residues (see S7 File). The locus 28040.t000001 was predicted to contain a single intron encoding 103 residues, however, read mapping and ORF analysis suggested that it represents only the 3’ sequence of this gene which is promised to have two introns putatively encoding 217 residues (see S8 File). The locus 29709.t000007 was predicted to encode 185 residues, however, it didn’t contain the complete WRKY domain. Instead, read mapping indicated that this locus is promised to encode 205 residues (see S9 File). The locus 29889.t000087 was predicted to encode 351 residues, however, read mapping and ORF analysis suggested that it represents only the 3’ sequence of the gene which is promised to encode 360 residues (see S10 File). The locus 30174.t000532 was predicted to encode 313 residues, however, read mapping indicated that this locus is promised to encode 308 residues (see S11 File). The locus 43951.t000001 was predicted to encode 195 residues, however, EST and read mapping indicated that another locus 30131.t000001 from scaffold43951 (1019 bp) also belongs to this gene, and the gene is promised to harbor three introns putatively encoding 318 residues (see S12 File). The locus 29848.t000101 was predicted to encode 211 residues, however, EST and read mapping indicated that this locus is promised to encode 330 residues (see S13 File). The locus 29848.t000100 was predicted to contain three introns encoding 139 residues, however, sequence analysis revealed that its WRKY domain is incomplete. Further read mapping indicated that this locus is promised to harbor three introns putatively encoding 242 residues. The first exon and the first intron of this gene were not annotated previously, whereas partial sequences of its fourth exon were not annotated or misannotated as the third intron (see S14 File). The locus 29736.t000019 was predicted to contain three introns encoding 562 residues, however, read mapping indicated that this locus is promised to harbor four introns putatively encoding 634 residues (see S15 File). The locus 29598.t000004 was predicted to contain three introns encoding 263 residues, however, read mapping indicated that this locus is promised to harbor two introns putatively encoding 353 residues and partial sequence of its first exon was misannotated as an intron (see S16 File). The locus 29644.t000015 was predicted to contain one intron encoding 105 residues, however, EST and read mapping indicated that another locus 29644.t000016 on the same scaffold also belongs to this gene, and the misannotation was resulted from the “TCTTGCTCCAGAAGAG” sequence that was misassembled into its first exon. Thereby, this locus is promised to harbor two introns putatively encoding 356 residues (see S17 File). The locus 28455.t000009 was predicted to contain four introns encoding 367 residues, however, read mapping indicated that this locus is promised to harbor two introns putatively encoding 317 residues (see S18 File). The locus 27996.t000002 was predicted to contain four introns encoding 466 residues, however, read mapping indicated that this locus is promised to harbor two introns putatively encoding 480 residues, and partial sequences of its first exon and intron were misannotated as an intron or an exon, respectively (see S19 File). The locus 28690.t000001 was predicted to contain three introns encoding 287 residues, however, read mapping indicated that this locus is promised to harbor two introns putatively encoding 339 residues (see S20 File).

**Table 1:** List of the 58 *RcWRKY* genes identified in this study.

Gene name	Scaffold ID	Predicted position	Locus ID	Transcript ID	Identified position	EST hits	Expressed	AS^a^	AS^b^	(Sub)group and comments	Deduced polypeptide				At_ortholog	Jc_ortholog
											Length (aa)	MW (kDa)	pI	GRAVY		
*RcWRKY01*	scaffold29949	50158–52013	29949.t000007	29949.m000123	49111–52359	-	Yes	-	Yes	I	484	52.89	7.04	-0.921	AtWRKY01	JcWRKY01
*RcWRKY02*	scaffold27613	207515–213650	27613.t000032	27613.m000639	207484–214016	2	Yes	-	Yes	I	562	60.78	6.84	-0.789	AtWRKY20	JcWRKY09
*RcWRKY03*	scaffold29929	510214–512861	29929.t000090	29929.m004587	509685–512932	-	Yes	-	-	I, misassembled	711	77.45	5.66	-0.675	AtWRKY20	JcWRKY10
*RcWRKY04*	scaffold28966	27612–23998	28966.t000003	28966.m000524	29398–23813	-	Yes	-	Yes	I	733	79.82	6.13	-0.761	AtWRKY02,34	JcWRKY11
*RcWRKY05*	scaffold29717	13600–10576	29717.t000002	29717.m000222	13200–10572	43	Yes	Yes	Yes	I	575	63.47	6.71	-1.043	AtWRKY33,25,26	JcWRKY07
*RcWRKY06*	scaffold29820	294372–291661	29820.t000050	29820.m001029	294438–289616	-	Yes	-	Yes	I, misannotated	598	65.52	7.59	-0.770	AtWRKY33,25,26	JcWRKY08
*RcWRKY07*	scaffold29635	203409–198815	29635.t000028	29635.m000468	203640–198543	1	Yes	-	Yes	I, misannotated	510	55.69	7.33	-0.812	AtWRKY04,03	JcWRKY06
*RcWRKY08*	scaffold30174	3380314–3384496	30174.t000563	30174.m009166	3380374–3384508	9	Yes	-	-	I, misannotated	524	57.12	7.75	-0.837	AtWRKY04,03	JcWRKY05
*RcWRKY09*	scaffold29805	189773–191900	29805.t000035	29805.m001504	187614–192328	-	Yes	-	Yes	I	474	52.00	8.59	-0.946	AtWRKY44	JcWRKY04
*RcWRKY10*	scaffold29687	15684–21009	29687.t000003	29687.m000562	15455–21624	1	Yes	-	Yes	I, misannotated	511	56.01	5.67	-0.811	AtWRKY32	JcWRKY02
*RcWRKY11*	scaffold29848	493883–492201	29848.t000095	29848.m004539	494292–491256	-	Yes	-	Yes	I, misannotated	451	45.84	8.65	-0.754	AtWRKY32	JcWRKY03
*RcWRKY12*	scaffold29771	4111–8719	29771.t000001	29771.m000072	4111–8868	-	Yes	-	-	IIc	215	24.33	6.83	-0.966	AtWRKY51,50,59,68	JcWRKY12
*RcWRKY13*	scaffold29739	137722–136858	29739.t000022	29739.m003586	137722–136462	-	Yes	-	Yes	IIc	159	18.03	5.98	-0.959	AtWRKY50,51,59,68	JcWRKY14
*RcWRKY14*	scaffold28644	112321–114401	28644.t000022	28644.m000915	112007–114711	-	Yes	-	-	IIc	168	19.40	5.49	-1.001	AtWRKY51,50,59,68	JcWRKY13
*RcWRKY15*	scaffold29929	718638–720485	29929.t000127	29929.m004624	718359–720760	-	Yes	-	-	IIc	203	22.79	9.15	-0.817	AtWRKY75,45	JcWRKY17
*RcWRKY16*	scaffold30190	612076–613585	30190.t000144	30190.m010908	611849–613809	-	Yes	-	-	IIc	194	22.28	9.30	-0.844	AtWRKY75,45	JcWRKY18
*RcWRKY17*	scaffold30147	2203761–2204393	30147.t000745	30147.m014474	2203665–2204574	-	Yes	-	-	IIc	164	18.96	9.49	-1.075	AtWRKY75,45	JcWRKY19
*RcWRKY18*	scaffold30174	2076040–2077754	30174.t000066	30174.m008669	2075781–2078009	-	Yes	-	Yes	IIc, misannotated	196	22.42	8.93	-0.622	AtWRKY43,24,56	JcWRKY21
*RcWRKY19*	scaffold30190	3026476–3027219	30190.t000514	30190.m011278	3026363–3027328	-	Yes	-	-	IIc	185	21.06	9.01	-0.661	AtWRKY56,24,43	JcWRKY20
*RcWRKY20*	scaffold28040	31334–28206	28040.t000001	28040.m000035	32051–27986	-	Yes	-	-	IIc, misannotated	217	24.87	9.34	-0.724	AtWRKY13	JcWRKY15
*RcWRKY21*	scaffold29709	41452–43070	29709.t000007	29709.m001171	41208–44408	-	Yes	-	Yes	IIc, misannotated	205	23.60	7.07	-1.020	AtWRKY12	JcWRKY16
*RcWRKY22*	scaffold29889	412384–414133	29889.t000087	29889.m003321	412309–414133	1	Yes	-	-	IIc, misannotated	360	39.98	6.32	-0.793	AtWRKY48	JcWRKY23
*RcWRKY23*	scaffold29693	677606–676301	29693.t000098	29693.m002060	677801–676041	-	Yes	-	-	IIc	310	34.44	6.97	-0.902	AtWRKY28,08	JcWRKY26
*RcWRKY24*	scaffold30076	1245583–1244284	30076.t000187	30076.m004623	1245777–1243970	-	Yes	-	-	IIc	317	35.19	6.40	-0.619	AtWRKY23	JcWRKY22
*RcWRKY25*	scaffold30174	3224608–3221434	30174.t000532	30174.m009135	3225599–3218630	1	Yes	-	Yes	IIc, misannotated	308	33.78	6.19	-0.909	AtWRKY57	JcWRKY24
*RcWRKY26*	scaffold29767	166469–163654	29767.t000010	29767.m000208	166564–163614	-	Yes	-	-	IIc	296	32.89	5.50	-0.700	AtWRKY49	JcWRKY38
*RcWRKY27*	scaffold28842	N/A	N/A	N/A	266259–272883	-	Yes	-	Yes	IIc, not predicted	117	13.57	6.30	-1.262	-	JcWRKY58
*RcWRKY28*	scaffold30131	4672–6497	30131.t000001	30131.m006850	4396–6722	7	Yes	-	Yes	IIa, misassembled	318	35.49	8.64	-0.803	AtWRKY40,18,60	JcWRKY27
	scaffold43951	916–31	43951.t000001	43951.m000016												
*RcWRKY29*	scaffold29848	538602–537211	29848.t000101	29848.m004545	538811–533980	1	Yes	-	Yes	IIa, misannotated	330	36.53	8.52	-0.629	AtWRKY40,18,60	JcWRKY28
*RcWRKY30*	scaffold29848	523071–522197	29848.t000100	29848.m004544	523248–521822	-	Yes	-	Yes	IIa, misannotated	242	27.84	9.63	-0.719	AtWRKY40,18,60	JcWRKY29
*RcWRKY31*	scaffold29842	273087–275527	29842.t000052	29842.m003555	272719–275658	1	Yes	Yes	Yes	IIb	580	63.10	5.95	-0.686	AtWRKY42,06,31	JcWRKY31
*RcWRKY32*	scaffold30010	386731–384306	30010.t000025	30010.m000675	386795–384065	2	Yes	-	-	IIb	652	70.28	5.89	-0.735	AtWRKY42,06,31	JcWRKY32
*RcWRKY33*	scaffold30076	678694–681282	30076.t000112	30076.m004548	678304–681837	1	Yes	Yes	Yes	IIb	498	53.86	8.01	-0.545	AtWRKY47	JcWRKY30
*RcWRKY34*	scaffold30064	205783–203340	30064.t000028	30064.m000506	205918–203262	-	Yes	-	-	IIb	559	61.32	6.42	-0.611	AtWRKY47	JcWRKY33
*RcWRKY35*	scaffold29736	182805–186919	29736.t000019	29736.m002023	182186–187534	-	Yes	-	Yes	IIb, misannotated	634	69.06	7.58	-0.837	AtWRKY72,61	JcWRKY35
*RcWRKY36*	scaffold29822	915514–919280	29822.t000159	29822.m003484	915472–919280	6	Yes	-	-	IIb	560	60.07	6.51	-0.606	AtWRKY72,61	JcWRKY37
*RcWRKY37*	scaffold30147	4428301–4432024	30147.t000358	30147.m014087	4428301–4432024	-	Yes	-	-	IIb	651	70.62	6.23	-0.765	AtWRKY72,61	JcWRKY36
*RcWRKY38*	scaffold29990	4397–6433	29990.t000001	29990.m000497	4081–6735	-	Yes	-	-	IIb	532	58.99	5.25	-0.814	AtWRKY09	JcWRKY34
*RcWRKY39*	scaffold29848	96608–94812	29848.t000020	29848.m004464	96843–94777	46	Yes	-	Yes	IId	321	34.61	9.54	-0.485	AtWRKY11,17	JcWRKY43
*RcWRKY40*	scaffold29598	24172–22752	29598.t000004	29598.m000445	24740–22377	-	Yes	-	Yes	IId, misannotated	353	39.92	9.75	-0.663	AtWRKY74,39	JcWRKY40
*RcWRKY41*	scaffold29644	80028–79578	29644.t000015	29644.m000187	81125–78861	45	Yes	Yes	Yes	IId, misassembled	356	38.95	9.43	-0.630	AtWRKY07	JcWRKY41
	scaffold29644	81125–80117	29644.t000016	29644.m000188												
*RcWRKY42*	scaffold29883	450119–452078	29883.t000063	29883.m002008	449401–452648	-	Yes	-	Yes	IId	353	39.75	9.66	-0.867	AtWRKY21,39,74	JcWRKY39
*RcWRKY43*	scaffold30170	1224146–1225662	30170.t000244	30170.m013832	1224113–1226229	1	Yes	-	-	IId	377	41.43	9.59	-0.773	AtWRKY07,15	JcWRKY42
*RcWRKY44*	scaffold28455	136512–134590	28455.t000009	28455.m000362	137020–134740	-	Yes	-	-	IId, misannotated	317	35.36	9.76	-0.584	AtWRKY21	JcWRKY44
*RcWRKY45*	scaffold30174	2058419–2060079	30174.t000060	30174.m008663	2058419–2060093	9	Yes	Yes	Yes	IIe	347	37.77	5.92	-0.676	AtWRKY22	JcWRKY47
*RcWRKY46*	scaffold30190	3016527–3017971	30190.t000512	30190.m011276	3016527–3018091	-	Yes	-	-	IIe	334	37.45	4.98	-0.601	AtWRKY29	JcWRKY45
*RcWRKY47*	scaffold30169	2000467–1998907	30169.t000358	30169.m006581	2000700–1998672	-	Yes	-	-	IIe	459	50.16	5.69	-0.814	AtWRKY27	JcWRKY48
*RcWRKY48*	scaffold30076	1384793–1383612	30076.t000212	30076.m004648	1385658–1383503	-	Yes	-	Yes	IIe	265	30.19	5.33	-0.988	AtWRKY69,69	JcWRKY46
*RcWRKY49*	scaffold30026	178343–179952	30026.t000025	30026.m001461	178328–180279	2	Yes	-	-	IIe	267	30.02	5.90	-1.064	AtWRKY69,69	JcWRKY50
*RcWRKY50*	scaffold27996	7134–9912	27996.t000002	27996.m000145	6705–10469	-	Yes	-	Yes	IIe, misannotated	480	52.06	5.28	-0.774	AtWRKY35,14	JcWRKY49
*RcWRKY51*	scaffold30190	3508463–3507055	30190.t000050	30190.m010814	3508665–3505132	2	Yes	Yes	Yes	III	338	38.01	5.41	-0.831	AtWRKY41,53	JcWRKY54
*RcWRKY52*	scaffold30174	1952662–1954347	30174.t000048	30174.m008651	1952428–1954708	-	Yes	-	-	III	333	37.80	5.38	-0.786	AtWRKY30	JcWRKY51
*RcWRKY53*	scaffold30169	1742567–1744656	30169.t000310	30169.m006533	1952662–1954347	-	Yes	-	Yes	III	370	41.17	5.54	-0.697	AtWRKY41,53	JcWRKY53
*RcWRKY54*	scaffold28690	13680–12224	28690.t000001	28690.m000025	1742258–1744988	-	Yes	-	-	III, misannotated	339	38.05	5.56	-0.663	AtWRKY41,53	JcWRKY52
*RcWRKY55*	scaffold29729	344149–342169	29729.t000063	29729.m002330	13973–11973	-	Yes	-	-	III	318	35.55	5.94	-0.704	AtWRKY55	JcWRKY55
*RcWRKY56*	scaffold29729	570667–568893	29729.t000103	29729.m002370	344520–341549	-	Yes	-	-	III	331	36.91	5.94	-0.693	AtWRKY55	JcWRKY55
*RcWRKY57*	scaffold29729	563671–564936	29729.t000102	29729.m002369	570667–568714	1	Yes	-	Yes	III	314	35.45	5.87	-0.603	AtWRKY70	JcWRKY57
*RcWRKY58*	scaffold29915	143043–141304	29915.t000015	29915.m000479	563637–565065	-	Yes	Yes	Yes	III	330	37.07	5.53	-0.667	AtWRKY70	JcWRKY56

^a^ Based on the EST data.

^b^ Based on the RNA sequencing data.

N/A, not available.

“-”, not detected.

**Fig 1:**
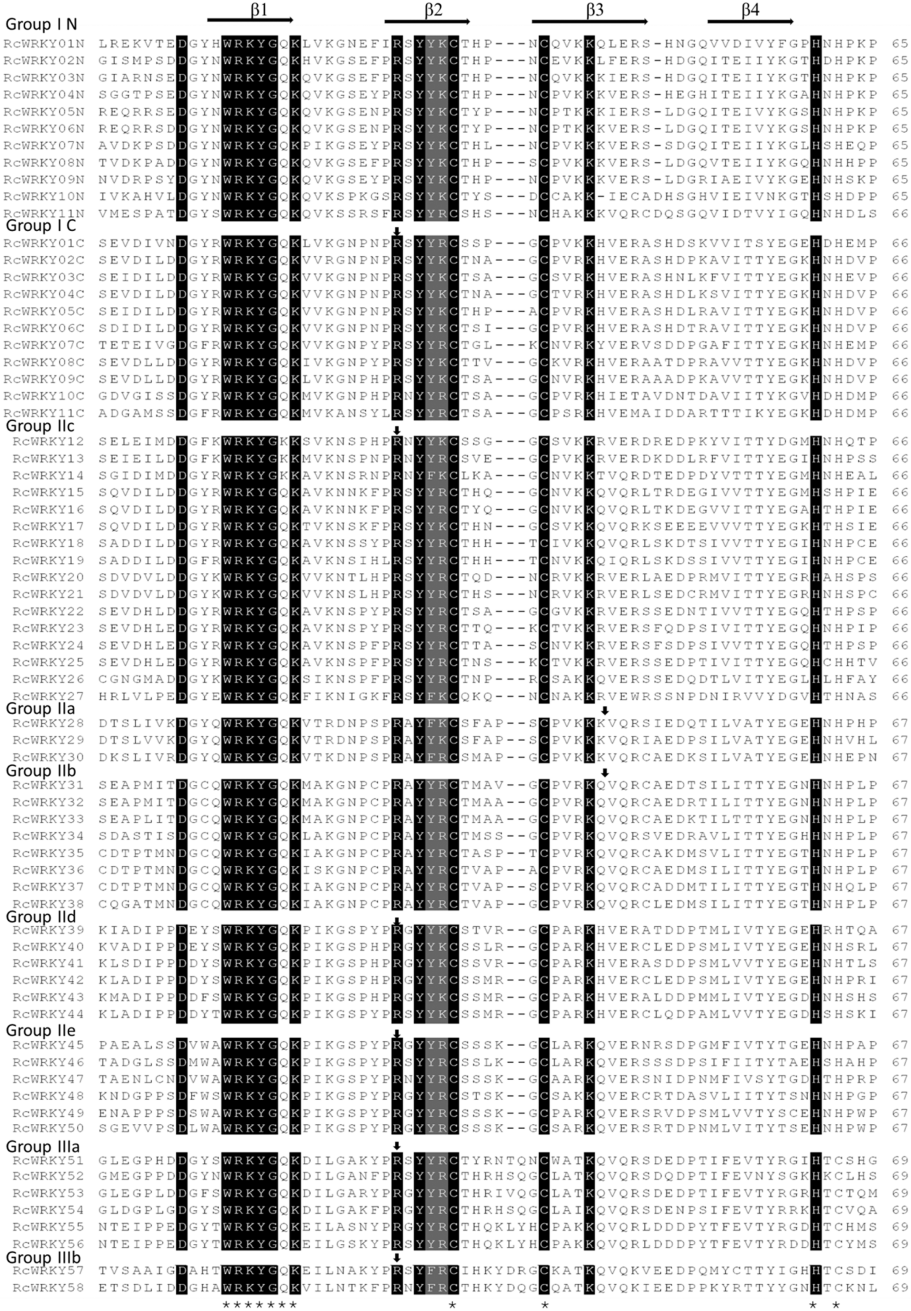
Comparison of the *WRKY* domain sequences from 58 RcWRKY proteins. WRKY^..^N/C represents the N or C-terminal *WRKY* domain of group I members, respectively. “-” has been inserted for the optimal alignment. Conserved amino acid residues are shown in gray and the highly conserved WRKYGQ/KK heptapeptide and C_2_H_2_/C and residues are indicated by “*”. The four β-strands are indicated by right arrows. For each (sub)group, the position of a conserved intron is indicated by a down arrow.

**Fig 2:**
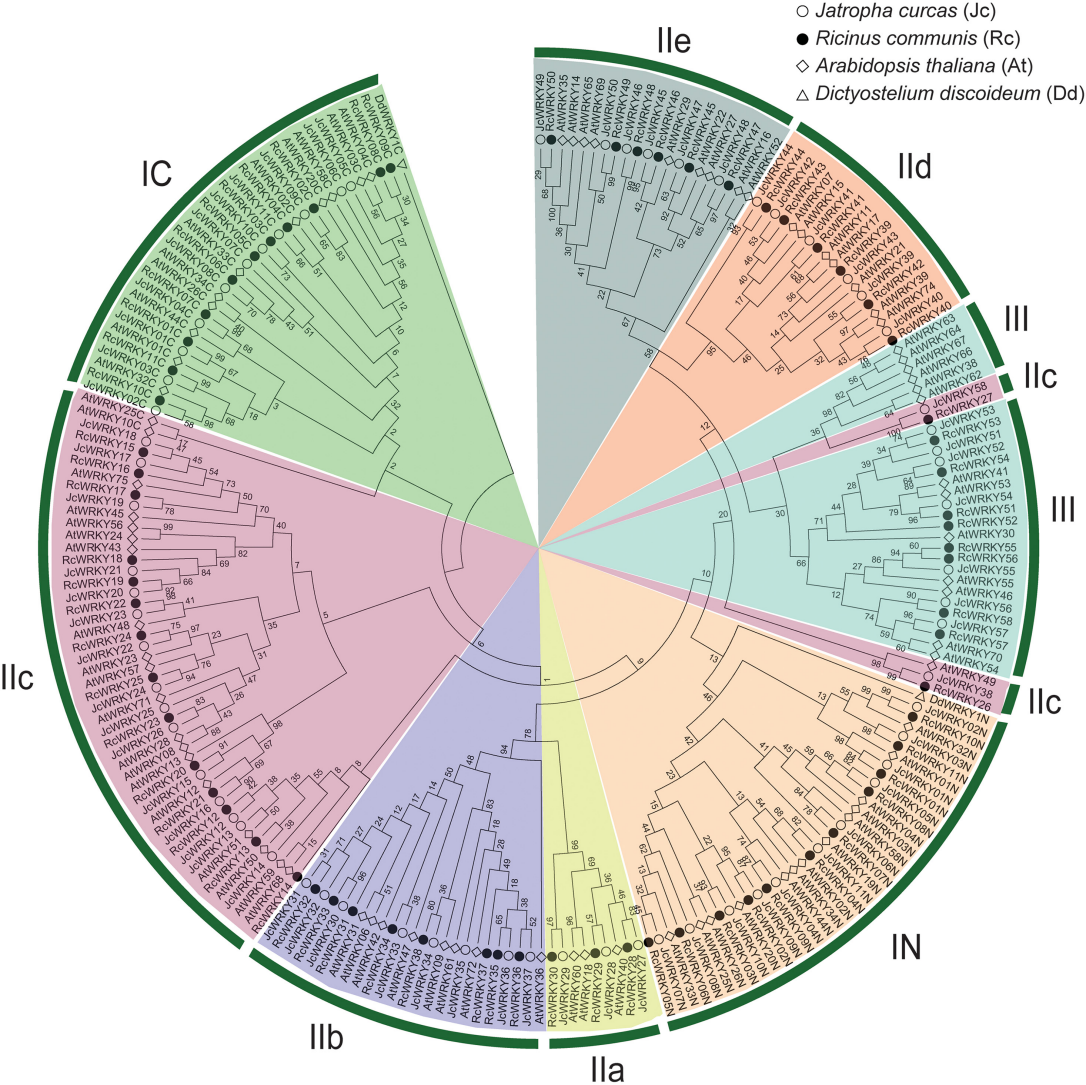
Phylogenetic analysis of *RcWRKY* proteins with *Arabidopsis* and physic nut homologs. The *WRKY* domains (WRKY^..^N/C representing the N and C-termini of group I members, respectively) extracted from deduced amino acid sequences were performed using MUSCLE and the phylogenetic tree adopting DdWRKY1C as an outgroup was constructed using bootstrap maximum likelihood tree (1000 replicates) method and MEGA6 software. The distance scale denotes the number of amino acid substitutions per site. The name of each (sub)group is indicated next to the corresponding group. Species and accession numbers are listed in Table 1 and S1 Table.

**Fig 3:**
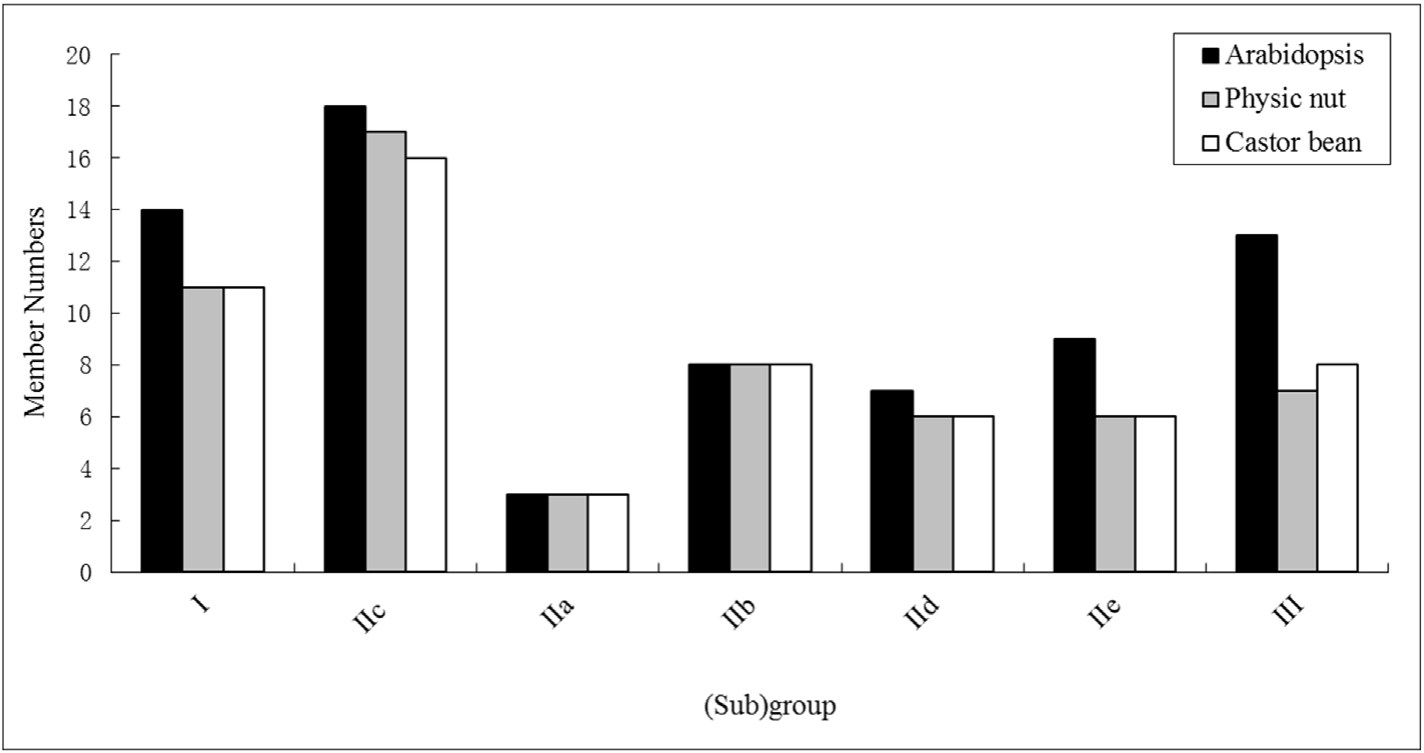
Distribution of the 58 *RcWRKY* genes and their *Arabidopsis* and physic nut homologs in subgroups.

Based on the structural features (Fig 1) and evolutionary relationships (Fig 2, see below), a systematic name was assigned to each of the 58 *RcWRKY* genes (Table 1). Eleven members that contain two WRKY domains and feature the C_2_H_2_-type zinc finger motif (N: Cx_4_Cx_22-23_HxH; C: Cx_4_Cx_23_HxH) were categorized into the group I, whereas the remainings that harbor a single WRKY domain were categorized into the group II (39 members, featuring the C_2_H_2_ zinc finger: Cx_4-5_Cx_23_HxH) or III (8 members, featuring the C_2_HC zinc finger: Cx_7_Cx_23_HxC) (Table 1 and Fig 1). *RcWRKY* genes of the group II were further divided into 5 subgroups, i.e., IIa (3), IIb (8), IIc (16), IId (6) and IIe (6) (Fig 3). As shown in Fig 2, RcWRKY26 and RcWRKY27 seem to form two new subgroups: RcWRKY26, JcWRKY38 and AtWRKY49 were clustered together and shown to be closer to the N-terminal WRKY domains, whereas RcWRKY27 and its ortholog JcWRKY58 were closer to the group III members. However, both of them exhibit a zinc finger pattern Cx_4_Cx_23_HxH as observed in the subgroup IIc and the C-terminal WRKY domains of group I members (Fig 1). Thereby, they were classed into the subgroup IIc in this study. Compared with *Arabidopsis*, castor bean and physic nut have fewer family members in any (sub)group. Although the total number of family members is the same between castor bean and physic nut, castor bean contains one more group III member but one fewer subgroup IIc (Fig 3).

Although most RcWRKYs harbor the conserved heptapeptide WRKYGQK, the WRKYGKK variety was also observed in three members (i.e. RcWRKY12, RcWRKY13 and RcWRKY14) (Fig 1) as seen in physic nut, *Arabidopsis* and other plant species [7,10,12]. Except for the automatic genome annotation, homology analysis showed that no cDNA sequences of the 58 identified *RcWRKY* genes were reported in any public database. Nevertheless, 20 members had EST hits in NCBI GenBank (as of Apr 2015). Though most of them had only one hit, we still observed that three members (*RcWRKY39*, *RcWRKY41* and *RcWRKY05*) matched more than 40 ESTs (Table 1). Further, read alignments against RNA sequencing data of root, leaf, flower, seed and endosperm supported the expression of other 38 *RcWRKY* genes. In addition, alternative splicing isoforms existing in 7 or 31 RcWRKY-encoding loci were supported by Sanger ESTs or RNA sequencing reads, respectively (Table 1).

As described above, the 1019-bp scaffold43951 was predicted to encode a WRKY domain-containing peptide. However, since it can be anchored to the 2696182-bp scaffold30131 sharing a 300-bp overlapping sequence, thus the scaffold30131 instead of scaffold43951 was counted as one WRKY-encoding scaffold. Among these 41 WRKY-encoding scaffolds, nine of them, i.e., scaffold30174 (5), scaffold29848 (4), scaffold30190 (4), scaffold30076 (3), scaffold29729 (3), scaffold29929 (2), scaffold30147 (2), scaffold29644 (2) and scaffold30169 (2), were shown to encode more than one *WRKY* genes, whereas the remainings encode a single one (Table 1).

**Fig 4:**
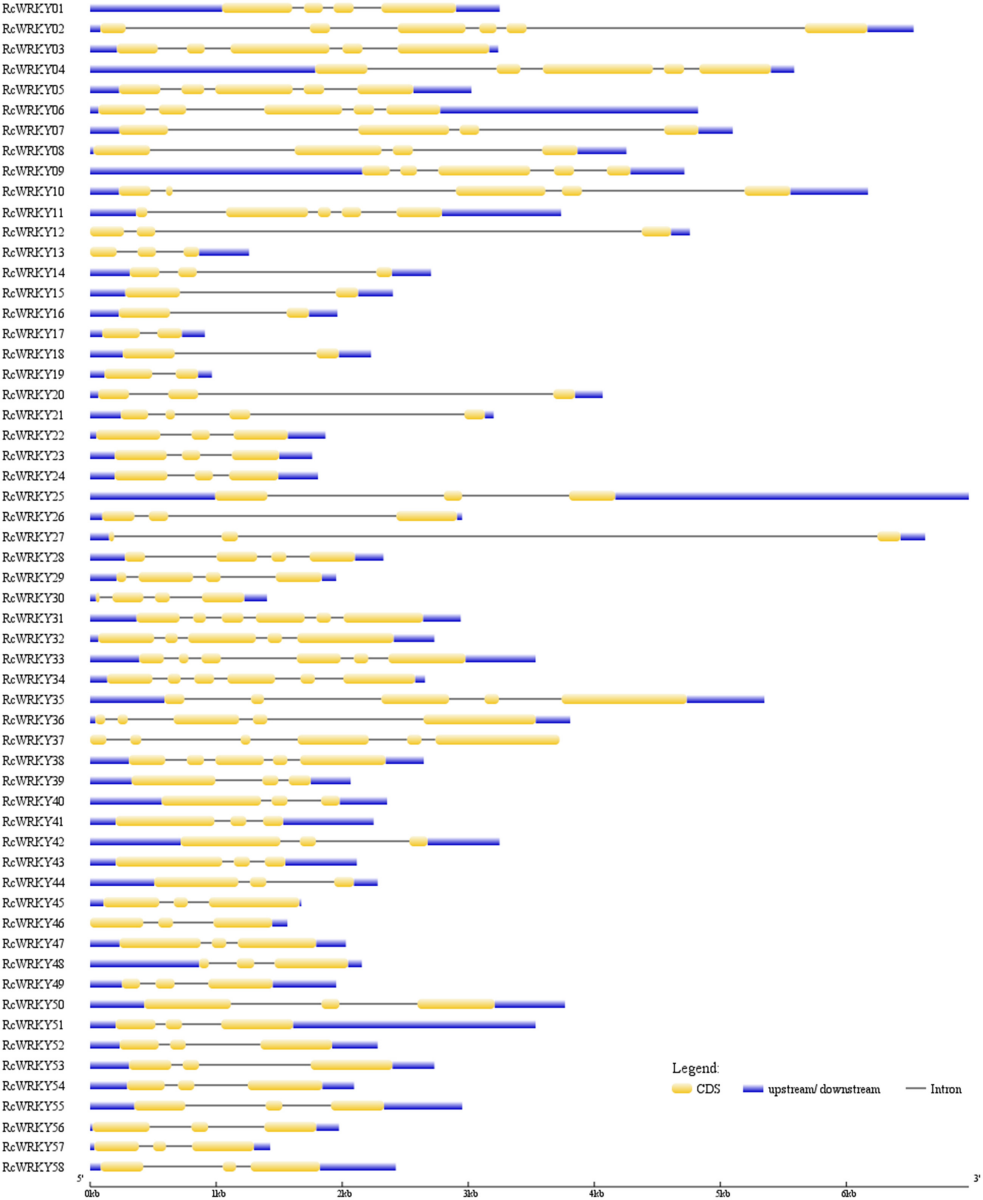
Exon-intron structures of the 58 identified *RcWRKY* genes. The graphic representation of the optimized gene models is displayed using GSDS.

The exon-intron structures of the 58 *RcWRKY* genes were investigated based on the optimized gene models. Though all the deduced polypeptides of the *RcWRKY* genes contain one or two complete WRKY domains (Fig 1), the length of these amino acid sequences is highly distinct (Table 1). Compared with the CDS length (354–2202 bp), the gene length (from start to stop codons) of *RcWRKYs* is even more variable (633–6280 bp) (Fig 4). All *RcWRKY* genes contain at least one intron in their CDSs: 5 have one intron; 30 (more than 51.7%) have two introns, which include all members of (sub)groups IId, IIe and III; 7 have three introns; 11 have four introns; and 5 have five introns (Fig 4). Except for *RcWRKY29*, similar exon-intron structures were also observed in physic nut [7], a plant species also belonging to the Euphorbiaceae family and having diverged from castor bean approximately 49.4 million years ago [20]. Although the peptide length is very similar, *RcWRKY29* (CDS, 993 bp) was shown to contain three introns (Fig 4); in contrast, its physic nut ortholog (*JcWRKY28*, CDS, 996 bp) has four introns [7]. Sequence analysis indicated that *RcWRKY29* has lost the second intron as observed in physic nut. Without any exception, all *RcWRKY* genes harbor one intron in the WRKY domain-coding sequences (the C-terminal WRKY domain of group I members) (Fig 1). In members of subgroups a and b, the conserved intron presents in the zinc finger motif (24 codons further towards the C-terminus), whereas in groups I and III, and subgroups c–e, the intron is located after the second base of the arginine codon close to the N-termini of the zinc finger motif (Fig 1). Similar results were also observed in *Arabidopsis* and other plant species [6,10], suggesting that this is a general feature of the entire gene family.

### Phylogenetic analysis of *RcWRKY* proteins

The homology analysis via Blastp showed that the 58 RcWRKYs have 56 or 36 counterparts in physic nut and *Arabidopsis*, respectively (Table 1), suggesting specific gene expansion and gene loss occurred in these plant species. Since the amino acid sequences beyond the WRKY domain are highly variable, the WRKY domain sequences were extracted from *D*. *discoideum*, *Arabidopsis*, physic nut and castor bean WRKY proteins, and used for the phylogenetic tree construction. *D*. *discoideum*, a slime mold closely related to the lineage of animals and fungi, was shown to encode a single group I-like *WRKY* gene which appears to be obtained via lateral gene transfer having occurred pre-date the formation of the WRKY groups in flowering plants [10,38]. The tree adopting DdWRKY1C as an outgroup was shown in Fig 2. According to the phylogenetic tree, a high number of *Arabidopsis* WRKY family members were grouped in pairs (Fig 2), corresponding to the occurrence of one whole-genome triplication event and two recent doubling events [39,40]. In contrast, few gene pairs were identified in castor bean as seen in physic nut (Fig 2). RcWRKY55 and RcWRKY56 were clustered together with their closest homolog in physic nut (JcWRKY55) (Fig 2). Both of them were clustered in scaffold29729 (spaced by 39 loci) (Table 1), indicating that they were resulted from proximal duplication after the divergence of castor bean and physic nut. In addition, the C-terminal WRKY domains of RcWRKY08 and RcWRKY09 were also clustered together apart from that of JcWRKY05 and JcWRKY06, however, the N-terminal WRKY domains of RcWRKY08 and RcWRKY09 were clustered with that of JcWRKY05 and JcWRKY04, respectively; moreover, the Blastp analysis indicated the ortholog of RcWRKY08 and RcWRKY09 is JcWRKY05 or JcWRKY04, respectively. Thereby, RcWRKY08 and RcWRKY09 are promised to emerge before the divergence of castor bean from physic nut. The homology analysis also suggested that the castor bean has lost the ortholog of JcWRKY25, since its ortholog was detected in another two Euphorbiaceae plants, i.e., cassava (*Manihot esculenta*) and rubber tree (*Hevea brasiliensis*) ([41,42] Zou et al., unpublished data).

### Protein properties and conserved motifs beyond the *WRKY* domain

The predicted RcWRKY proteins have an average length of about 383 residues, with the minimum of 117 residues for RcWRKY27 and the maximum of 733 residues for RcWRKY04, whereas the average molecular weight is about 42.22 kDa, with the minimum of 13.57 kDa for RcWRKY27 and the maximum of 79.82 kDa for RcWRKY04, which is consistent with their peptide length. Although harboring an average *p*I value of 7.08, more than 58.62% RcWRKY proteins have a *p*I value of less than 7, indicating that most of them are acid. All RcWRKY proteins were predicted to harbor a GRAVY value (average: -0.78) of less than 0, indicating their hydrophilic feather. According to the 2ZIP analysis, two RcWRKY proteins (i.e. RcWRKY29 and RcWRKY33) were predicted to harbor a conserved Leu zipper motif, which was shown to be involved in dimerization and DNA binding [43,44]. The HARF motif was identified in three subgroup IId members, RcWRKY39, RcWRKY41 and RcWRKY43, although little is known about its exact function. LxxLL, a coactivator motif, was not found in any of the 58 RcWRKY proteins. In contrast, the active repressor motif LxLxLx were identified in two out of the three subgroup IIa members (i.e. RcWRKY28 and RcWRKY30) and four out of eight subgroup IIb members (i.e. RcWRKY35, RcWRKY36, RcWRKY37 and RcWRKY38).

**Fig 5:**
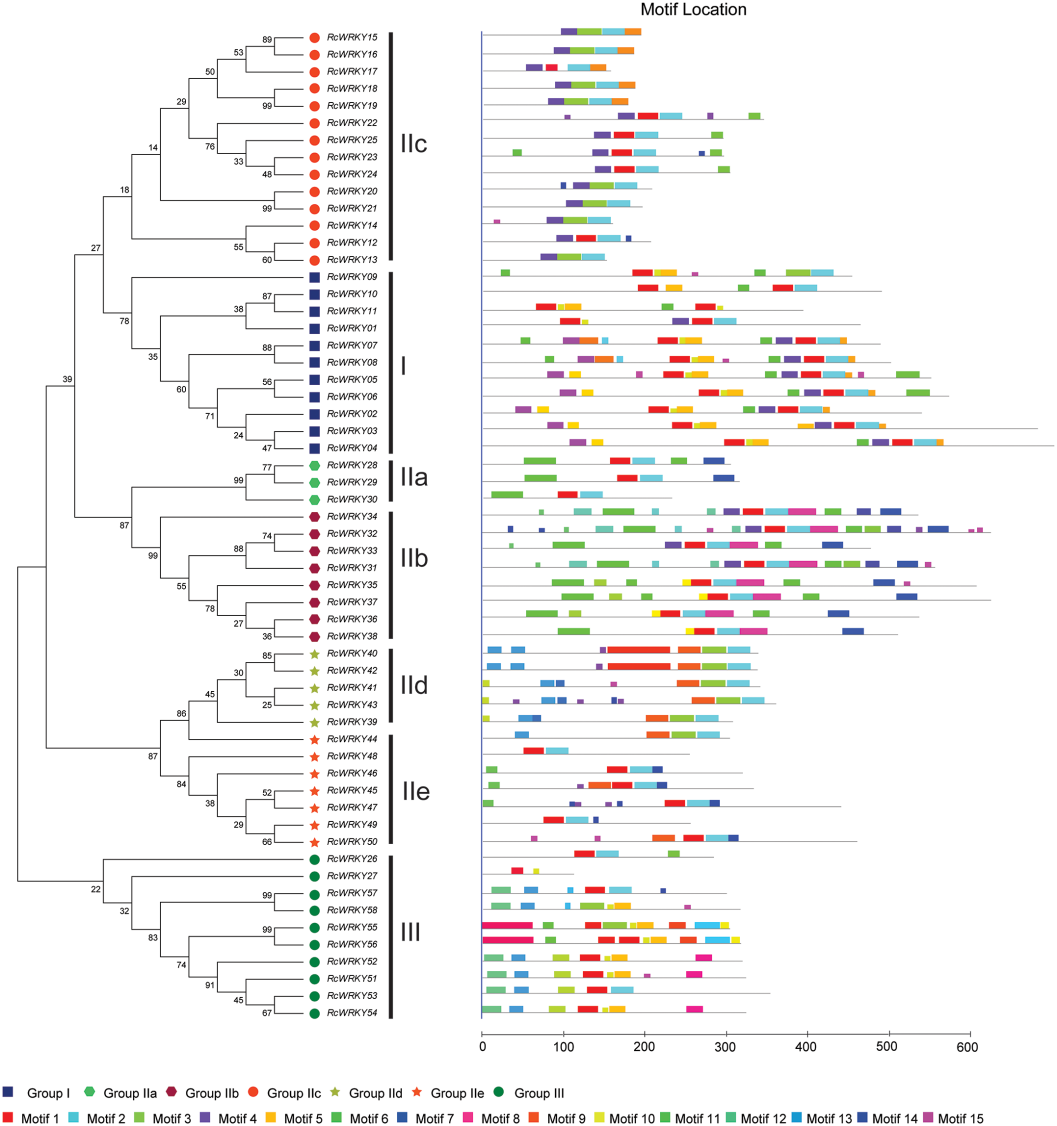
Structural and phylogenetic analysis of *RcWRKY* proteins. The unrooted phylogenetic tree resulting from the full-length amino acid alignment of all the RcWRKY proteins is shown on the left side of the figure. The different colored balls at the bottom of the figure indicate different groups. The distribution of conserved motifs among the *RcWRKY* proteins is shown on the right side of the figure. Different motif types are represented by different color blocks as indicated at the bottom of the figure. The same color in different proteins indicates the same group or motif.

To better understand the similarity and diversity of motif compositions among different RcWRKYs, a phylogenetic tree based on the full-length RcWRKY proteins was constructed (Fig 5) and the motifs in RcWRKY protein sequences were predicted using MEME (Fig 5, Table 2). Among 15 identified motifs, motifs 1, 2, 3 and 10 were characterized as WRKY domains that are broadly distributed across the RcWRKYs; the motif 9, characterized as the nuclear localization signal (NLS) sequence, was found in all members of subgroups IIa and IIb. In contrast, little information is available for other motifs: the motif 4 was found in most members of the group I, subgroups IIb and IIc; motifs 5 and 10 were found in most members of groups I and III; the motif 13 was found in the subgroup IId and group III; motifs 6, 7, 8 and 11 are limited to subgroups IIa and IIb members; motifs 12 and 15 are unique in the group III or I, respectively.

**Table 2:** Motif sequences of 58 *RcWRKY* proteins identified by the MEME tools.

Motif	E-value	Sites	Width	Best possible match
Motif1	3.0E-1047	50	26	DGYRWRKYGQKMVKGNPYPRSYYRCT
Motif2	5.2E-871	50	29	GCPVRKHVERCAEDPTMVITTYEGEHNH
Motif3	9.7E-326	17	31	DILDDGYRWRKYGQKPIKNSPHPRGYYRCTH
Motif4	8.80E-114	26	21	KKKGEKKIREPRFAFQTRSEV
Motif5	8.80E-89	17	21	ERDHDGQIFEIIYKGTHNCPK
Motif6	3.90E-95	11	41	LEVLQAELERMKEENERLRQMLTQMCKNYNALQMHFCELMQ
Motif7	6.20E-78	10	27	VEAATAAITADPNFTAALAAAITSIIG
Motif8	1.20E-72	8	36	AAMAMASTTSAAASMLLSGSSSSADGIMNHNTF
Motif9	5.90E-58	8	29	CASSGRCHCSKRRKMRVKRVIRVPAISNK
Motif10	4.20E-30	18	8	NCPAKKKV
Motif11	3.40E-29	8	21	MASISASAPFPTITLDLTHS
Motif12	3.00E-24	6	25	WEQHTLVGELIQGRELARQLRIHLN
Motif13	1.10E-22	14	18	LVQKIVSKFKKVLSLLNW
Motif14	1.80E-17	10	7	NHHHHHH
Motif15	4.00E-20	7	21	SPYITIPPGLSPTALLDSPVF

### Distinct expression profiles of *RcWRKY* family members in various tissues

**Fig 6:**
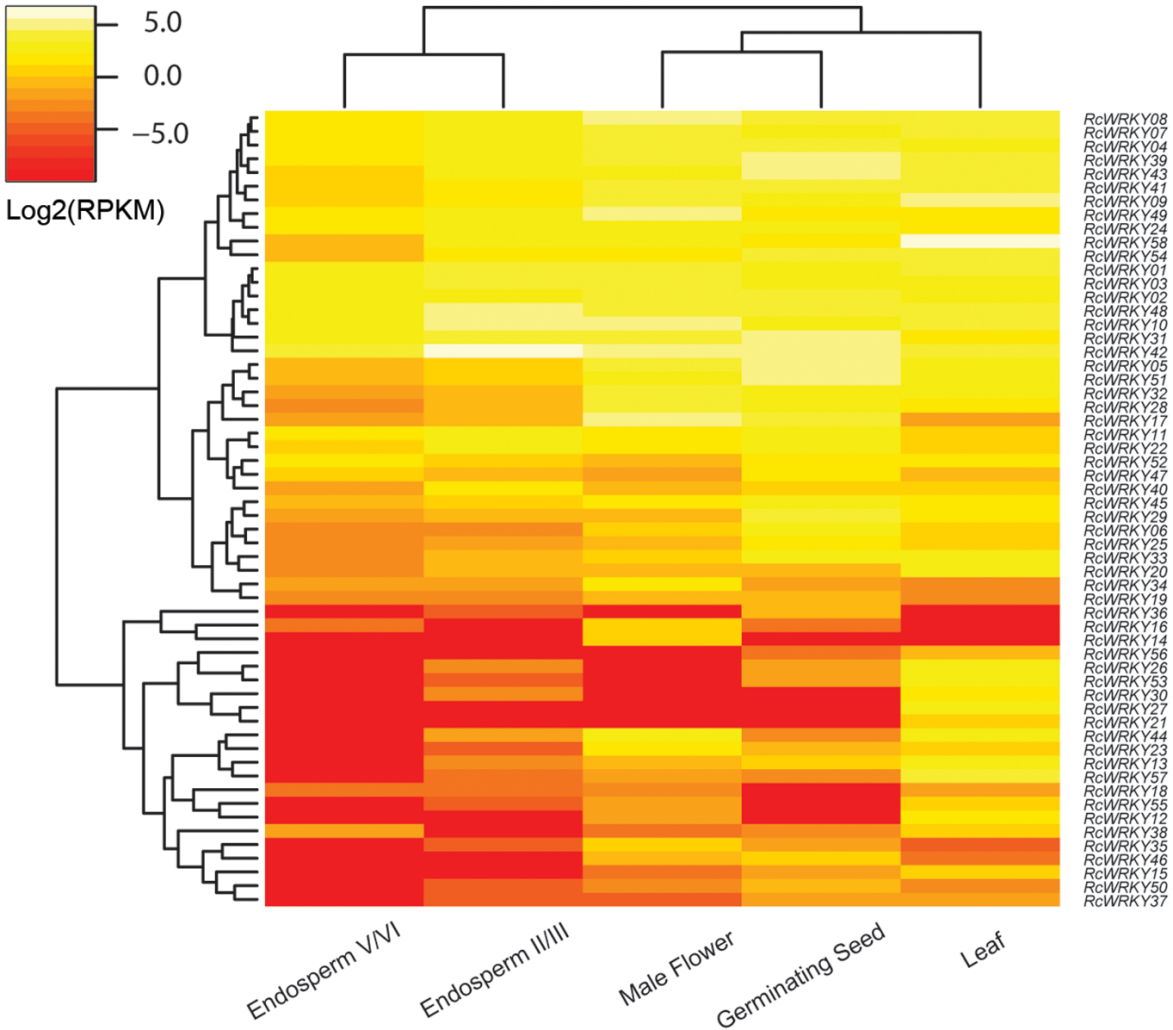
Expression profiles of the 58 *RcWRKY* genes in leaf, flower, endosperm II/III, endosperm V/VI and seed. Color scale represents RPKM normalized log_2_ transformed counts and red indicates low expression and yellow indicates high expression.

To gain more information on the role of *WRKY* genes in castor bean, RNA sequencing data of leaf, male flower, endosperm and seed were investigated. The expanding true leaves, appearing after the first cotyledons and leaf-pair, represent the leaf tissue; the male flower tissue includes pollen and anthers but excludes sepals; the germinating seed tissue was obtained by soaking dry seeds in running water overnight followed by germination in the dark for 3 days; and the endosperm tissue includes two representative stages termed stages II/III (endosperm free-nuclear stage) and V/VI (onset of cellular endosperm development) [24]. Results showed that the expression of all 58 *RcWRKY* genes were detected in at least one of the examined tissues, i.e., 55 in leaf, 51 in male flower, 51 in endosperm and 51 in seed (Fig 6). And the cluster analysis showed that the expression pattern of *RcWRKY* genes was more similar between flower and seed, and two stages of endosperm (Fig 6), corresponding their biological characteristics. Among three genes not detected in leaves, *RcWRKY14* was only and lowly expressed in male flowers, although previous qRT-PCR analysis showed that it was also expressed in roots and fruits at 50 days post-anthesis [21]. In contrast, its ortholog *JcWRKY14* in physic nut was shown to be highly expressed in stems (shoot cortex), roots and seeds of late development (i.e. filling and maturation) stage as well as leaves [8]. *RcWRKY16* was expressed in male flowers, germinating seeds and stage V/VI endosperm, and the expression levels were considerably low in seeds and endosperm, which is consistent with the qRT-PCR result [21]. Similar expression profile of its ortholog *JcWRKY18* in physic nut was also observed [8]. *RcWRKY36* was detected in stage II/III endosperm and germinating seeds, and the previous qRT-PCR analysis indicated that this gene was highly expressed in roots [21]. In physic nut, the expression of its ortholog *JcWRKY37* was also shown to be restricted to roots [8]. Among seven genes not detected in male flowers, all of them were also not detected in stage V/VI endosperm; except for *RcWRKY36*, *RcWRKY21*, *RcWRKY26*, *RcWRKY27*, *RcWRKY30*, *RcWRKY53* and *RcWRKY56* were all detected in leaves; *RcWRKY21* and *RcWRKY27* were detected only in leaves; besides leaves, *RcWRKY26* and *RcWRKY30* were also detected in stage II/III endosperm, though the expression level was extremely low; *RcWRKY53* was also detected lowly in stage II/III endosperm and germinating seeds; and *RcWRKY56* was also detected in germinating seeds. Among seven genes not detected in endosperm, *RcWRKY14*, *RcWRKY21*, *RcWRKY27* and *RcWRKY56* were discussed above; *RcWRKY12* was detected in leaves and male flowers which is consistent with the qRT-PCR result [21] and the expression pattern of its ortholog *JcWRKY12* in physic nut [8]; *RcWRKY15* and *RcWRKY56* were lowly expressed in all other samples examined, in contrast, their physic nut orthologs *JcWRKY17* and *JcWRKY45* were shown to be highly expressed in roots, lowly expressed in stems and leaves, but not detected seeds of both early and late development stages [8]. Among seven genes (i.e. *RcWRKY12*, *RcWRKY14*, *RcWRKY18*, *RcWRKY21*, *RcWRKY27*, *RcWRKY30* and *RcWRKY55*) not detected in germinating seeds, *RcWRKY12*, *RcWRKY14*, *RcWRKY21* and *RcWRKY27* were discussed above. *RcWRKY18* was lowly expressed in all other samples examined. Compared with other tissues [21], qRT-PCR analysis showed that *RcWRKY18* was considerably more expressed in roots, which is consistent with the root-preferred expression of its ortholog *JcWRKY21* in physic nut [8]. *RcWRKY30* was only detected in leaves and stage II/III endosperm, whereas its physic nut ortholog *JcWRKY29* was shown to be lowly expressed in leaves, stems and roots, but not seeds [8]. *RcWRKY55* was lowly expressed in all other examined samples except for stage V/VI endosperm, in contrast, the expression of its physic nut ortholog *JcWRKY55* was shown to be restricted to roots and the expression level was extremely low [8].

Based on the RPKM annotation, the total transcript abundance of *RcWRKY* genes in endosperm tissue (including both stages II/III and V/VI, with RPKM = 337.14 or 123.03, respectively) was relatively lower than that in other three tissues, i.e., leaf (RPKM = 585.83), male flower (RPKM = 576.19) and germinating seed (RPKM = 560.44) (Fig 6). *RcWRKY58* (RPKM = 139.35), the most abundant *WRKY* family member in leaves, was detected in all other tissues examined, though the expression levels were considerably low. Similarly, its ortholog *AtWRKY70* in *Arabidopsis* was also shown to be constitutively expressed during all leaf development stages [45,46]. Functional analysis indicated that *AtWRKY70* plays a pivotal role in salicylic acid (SA)- and jasmonic acid (JA)-dependent defense signaling [47,48]. Moreover, *AtWRKY70* together with *AtWRKY54* co-operate as negative regulators of leaf senescence and modulate osmotic stress tolerance by regulating stomatal movement [46,49,50]. Besides highly expressed in leaves, its ortholog *JcWRKY56* in physic nut was even more abundant in seeds of early development stage, and the expression levels in roots, stems and leaves were up-regulated by stresses such as drought and salinity [8]. *RcWRKY49* (RPKM = 49.08), the most expressed *RcWRKY* gene in male flowers, was also lowly detected in other tissues, which is consistent with the qRT-PCR result [21]. In contrast, its ortholog *JcWRKY50* in physic nut was expressed highly in roots, moderately in leaves and lowly in stems, and the expression levels were regulated by at least one of tested abiotic stresses, i.e. drought, salinity, phosphate starvation and nitrogen starvation [8]. Among two highly abundant *RcWRKY* genes in germinating seeds, *RcWRKY42* (RPKM = 49.46) also represented the most expressed member in stages II/III (RPKM = 81.26) and V/VI (RPKM = 21.86) endosperm, whereas *RcWRKY05* (RPKM = 47.04) was expressed moderately in male flowers (RPKM = 19.96) and leaves (RPKM = 7.90), lowly in stages II/III (RPKM = 2.61) and V/VI (RPKM = 0.62) (Fig 6). Although not detected in two seed development stages, the physic nut ortholog (*JcWRKY39*) of *RcWRKY42* was highly expressed in roots, leaves and stems, and the expression levels were regulated by nitrogen starvation [8]. The expression levels of the physic nut ortholog (*JcWRKY07*) of *RcWRKY05* were shown to be high in roots, leaves and early developmental seeds, and extremely low in stems [8]. The response of *JcWRKY07* to drought, salinity and phosphate starvation stresses was observed in roots [8]. *AtWRKY33*, an *Arabidopsis* ortholog of *RcWRKY05* was shown to function as a positive regulator of resistance toward the necrotrophic fungi *Alternaria brassicicola* and *Botrytis cinerea* [51,52], and gene overexpression can increases salt and heat tolerance [53,54].

As mentioned above, the total *RcWRKY* transcripts in stage II/III endosperm was two folds more than that in stage V/VI endosperm. Among 51 *RcWRKY* genes detected in endosperm, 34 members had a RPKM value exceeding 0.5 in at least one stage of developing endosperm (stages II/III and V/VI). Differential expression analysis indicated that 23 out of the 32 down-regulated *RcWRKY* genes and one out of two up-regulated genes exceeded two folds (Fig 6), suggesting their putative regulatory role in early endosperm development.

In addition, *RcWRKY* genes are promised to be involved in the ABA-mediated seed filling. *In vivo* experiment showed that endogenous ABA levels were closely associated with storage material accumulation in developing castor bean seeds [55]. *In vitro*, exogenous ABA also enhanced the dry weight (including the accumulation of soluble sugar and total lipid content) of developing seeds cultured in a nutrient medium [56]. After the application of 10 μM ABA for 24 h, differential gene expression analysis indicated that 2568 genes were up or down-regulated at least two folds [56], which was shown to include 13 out of the 58 *RcWRKY* genes (S21 File). Among them, eleven (four group I members, two subgroup IId members, one subgroup IIa member, one subgroup IIb member, one subgroup IIc member, one subgroup IIe member and one group III member) were significantly up-regulated, whereas only two (one subgroup IIe member and one group III member) were down-regulated. *RcWRKY41*, the most up-regulated gene (more than 250 folds) (S21 File), was highly expressed in germinating seeds, leaves and male flowers (Fig 6), which is consistent with its high representative in Genbank EST database (Table 1); its ortholog *AtWRKY11* in *Arabidopsis*, was also shown to be constitutively expressed and act as negative regulators of basal resistance to *Pseudomonas syringae* [57]. *RcWRKY28*, the second highly up-regulated gene (more than 15 folds) (S21 File), was expressed more in male flowers and germinating seeds than in leaves and endosperm, though its expression level was considerably lower in stage V/VI endosperm as compared with stage II/III (Fig 6); *AtWRKY40*, its ortholog in *Arabidopsis*, was also induced by ABA and acts as a transcriptional repressor in ABA signaling and abiotic stress but a positive regulator in effector-triggered immunity [58–63]. *RcWRKY17*, a group IIc member preferring to express in male flowers, female flowers and germinating seeds, was up-regulated for more than nine folds upon the ABA application; its ortholog *AtWRKY75* in *Arabidopsis*, was shown to response to phosphate starvation, water deprivation, ethylene stimulus and biotic stress, and participate in lateral root development, leaf senescence and galactolipid biosynthesis [64–67]. *RcWRKY45*, a group IIe member preferring to express in germinating seeds and fruits at 50 days post-anthesis [21], was up-regulated for more than seven folds by ABA; *AtWRKY22*, its ortholog in *Arabidopsis*, was involved in dark-induced leaf senescence and submergence-mediated immunity [68–69]. These results suggested the putative role of RcWRKYs in the ABA signaling.

## Conclusions

Based on the genome and transcriptome datasets, in the current study, a total of 58 *WRKY* genes were identified from castor bean, one of the most important non-food oilseed crops in the Euphorbiaceae family. According to the structural features and evolutionary relationships of the present WRKY domains, the identified *RcWRKY* genes were assigned to the group I, group II (subgroup a-e) and group III. The WRKY domain pattern was characterized as WRKYGQ/KKx_13_Cx_4-7_Cx_22-23_HxH/C. Compared with *Arabidopsis* that feathers a high number of duplicate genes, few gene pairs were identified in the *RcWRKY* gene family, corresponding to no recent whole-genome duplication event occurred in castor bean. Comparative genomics analysis also indicated that one gene loss, one intron loss and one recent proximal duplication occurred in the *RcWRKY* gene family as compared with physic nut, another Euphorbiaceae plant species underwent no recent whole-genome duplication event. Although only 20 family members had EST hits in public database, the expression of all 58 *RcWRKY* genes was supported by RNA sequencing reads derived from root, leaf, flower, seed and endosperm. Compared with tissues such as leaf, male flower and germinating seed, the total expression level of *RcWRKY* genes in endosperm tissue was shown to be relatively low. Distinct gene expression profiles were also observed in different developmental endosperm. Compared with stage II/III endosperm, 23 out of the 54 endosperm-expressed *RcWRKY* genes were down-regulated at least two folds at stage V/VI, whereas only one member was shown to be significantly up-regulated, suggesting their key regulatory role in early endosperm development. In a word, results obtained from this study not only provide global information in understanding the molecular basis of the *WRKY* gene family in castor bean, but also provide a useful reference to investigate the gene family expansion and evolution in Euphorbiaceus plants such as *Hevea brasiliensis* and *Manihot esculenta*, and other plant species that underwent recent whole-genome duplication events.

## Supporting Information

S1 File The gene model for *RcWRKY03*. (PDF)

S2 File The gene model for *RcWRKY06*. (PDF)

S3 File The gene model for *RcWRKY07*. (PDF)

S4 File The gene model for *RcWRKY08*. (PDF)

S5 File The gene model for *RcWRKY10*. (PDF)

S6 File The gene model for *RcWRKY11*. (PDF)

S7 File The gene model for *RcWRKY18*. (PDF)

S8 File The gene model for *RcWRKY20*. (PDF)

S9 File The gene model for *RcWRKY21*. (PDF)

S10 File The gene model for *RcWRKY22*. (PDF)

S11 File The gene model for *RcWRKY25*. (PDF)

S12 File The gene model for *RcWRKY28*. (PDF)

S13 File The gene model for *RcWRKY29*. (PDF)

S14 File The gene model for *RcWRKY30*. (PDF)

S15 File The gene model for *RcWRKY35*. (PDF)

S16 File The gene model for *RcWRKY40*. (PDF)

S17 File The gene model for *RcWRKY41*. (PDF)

S18 File The gene model for *RcWRKY44*. (PDF)

S19 File The gene model for *RcWRKY50*. (PDF)

S20 File The gene model for *RcWRKY54*. (PDF)

S21 File List of 13 differentially expressed *RcWRKY* genes upon the ABA treatment. (PDF)

S1 Table List of the accession numbers of the WRKYs identified in *Arabidopsis* (72) and physic nut (58). (XLSX)
