# Supplementary material for: Gene Structures, Evolution and Transcriptional Profiling of the WRKY Gene Family in Castor Bean (Ricinus communis L.)
Source: PLoS One. 2016 Feb 5;11(2):e0148243. doi: 10.1371/journal.pone.0148243 (PMC4743969; doi:10.1371/journal.pone.0148243)
Supplement: S1 File — (PDF) [file pone.0148243.s001.pdf]

S1 File. The gene model for *RcWRKY03*. The coding region is marked with uppercase letters, above which is its deduced amino acids. The transcribed untranslated regions, including 5' UTR, intron and 3' UTR sequences, are marked with lowercase letters. The start and stop codons are blacked and the misassembled "TTNNNTTGAC" sequence is boxed.

1 ttcaaagacagtgtcttctctgattatctcagtaattttaatttgcaatctttaatctgc

61 aattagtttcattctcatctccaaaaacgggaactgaaaaacttcattaccaagtccccg

121 taaacgcacgctctctccatccattttcaaaaattttaaatgacggtcaggtgactctta

1 M M D P N P P P P

181 tacttccctcctctaaatccaccgtctccgcca**ATG**ATGGACCCCAATCCACCACCACCG

10 S P P R Q Q P S F A D S E T L E M A S I

241 TCACCGCCGCGGCAACAGCCTTCCTTCGCCGACTCCGAAACCCTAGAAATGGCCTCCATT

30 N D A L N D T S T S Q N E S S R G S I A

301 AATGATGCTCTAAACGACACCTCCACTAGCCAAAACGAGAGTAGCAGAGGAAGCATAGCG

50 E R R A A K C G F K A D R I N T A R F R

361 GAGCGAAGAGCCGCCAAATGCGGTTTTAAAGCAGATAGAATTAATACGGCGCGATTCCGT

70 T I T P L T S P A A A V R S P C I

421 ACAATCACTCCCTTGACTTNNNTTGACTTCGCCGGCCGCGGCTGTCCGCTCGCCGTGTAT

87 T I P P G I S P T A L L D S P I M L P N

481 AACTATTCCGCCTGGTATTAGCCCCACCGCGCTTCTCGATTCACCGATTATGCTGCCAAA

107 C Q

541 TTGTCAGgtcaatttcatttatttattttcatttccgtttcttagctttgcaattttctt

601 ttaatttttactaattaaaatttttaatgtaacatgtcgattgcaaatttagaaaaaaa

661 acatttaatatgcatgttagcttagtaaaacgacaaatgatttcgtgtttgatgtttta

109 P

721 atgtgttttattatatattaatttttctcttttattttattttactttttgggtgcagCC

110 S P T T G S F P F P L M N C G S S A T P

781 TTCTCCAACTACAGGAAGTTTCCCGTTTCCGCTTATGAATTGTGGAAGTTCAGCGACTCC

130 A D V E N S S N G G S Y T L K P Q N G F

841 TGCTGACGTGGAGAATAGTAGTAATGGCGGTTCCTACACATTGAAGCCTCAAAATGGCTT

150 A A L E A E

901 TGCTGCCTTAGAAGCTGAGgtacttgtttaagtaatgatatttacgcaagaagcagcagc

961 tatgtaacctcttttaattgttcatacgggtcttggttcggtacaatctgatttttctg

1021 cattaaatagcatcgtctacttgatagcactatatgaattgtgtaattttatcaatgttt

156 R S N A G D

1081 caactgagacttatatgccttctgcatttttactcttgggtagAGAAGTAATGCTGGTG

162 F Q E V G S L M E F E F P V E F L K Q A

1141 ACTTTCAAGAAGTTGGCTCGTTAATGGAATTTGAATTCCCAGTGGAGTTCTTAAAGCAAG

182 I T E S Y A E N S A T D I K V L N S M V

1201 CTATCACAGAGAGTTATGCTGAAAATTCGGCAACTGACATCAAAGTCTTAAATAGCATGG

202 T D A N L S D V Q M D S I P E E N V G T

1261 TTACAGATGCCAACTTGAGTGATGTGCAAATGGACTCCATCCCGGAAGAAAATGTTGGGA

222 C H P E E E E K G R L S A T G I A R N S

1321 CATGCCATCCTGAGGAAGAAGAGAAAGGGAGACTCTCTGCAACAGGGATTGCAAGGAATT
242 E D G Y N W R K Y G Q K Q V K G S E F P
1381 CAGAAGATGGATACAATTGGAGAAAGTATGGACAGAAGCAAGTCAAAGGGAGTGAATTCC
262 R S Y Y K C T H P N C Q V K K K I E R S
1441 CAAGGAGCTACTATAAGTGTACCCACCCAAATTGTCAGGTAAAGAAAAAAATAGAGCGTT
282 H D G Q I T E I I Y K G T H N H P K P Q
1501 CTCATGATGGTCAGATAACAGAAATCATCTATAAAGGTACCCATAATCACCCTAAACCTC
302 P S R R A H V G S T S S F D E V P E I D
1561 AGCCTAGTCGCCGAGCACATGTTGGATCGACTTCTTCATTTGATGAGGTGCCAGAGATTG
322 E G N A T C F K V E I G S A W K N P Q P
1621 ATGAAGGGAATGCAACCTGTTTCAAAGTAGAAATTGGGTCTGCTTGGAAAAATCCGCAAC
342 G S N G G L E R T S S A S V V T E L S D
1681 CAGGGTCCAATGGGGGTCTGGAAAGGACGTCTTCAGCATCTGTTGTGACAGAGCTTTCTG
362 P L S T T Q G K S I G T F E S A G T P E
1741 ATCCTCTATCAACCACTCAAGGAAAATCTATTGGTACATTTGAATCAGCAGGAACACCAG
382 L S S T L V S N D D D D D G A T Q G S I
1801 AGCTTTCTTCCACACTTGTTAGTAATGATGACGATGACGACGGGGCCACTCAGGGAAGCA
402 S L G V D A D I E E S E S K R R
1861 TATCACTTGGAGTGGATGCTGATATTGAGGAATCCGAGTCAAAAAGAAGgtatttatctc
1921 tgttctttgctgtgtatctgcatagcagaagaacatgataagttgtttctatttctttct
418 K I E S C L V E T
1981 tttttttttaatttttattttctcacgttcagGAAAATAGAAAGCTGCTTGGTTGAAACA
427 S L S S R A V R E P R V V V Q I E S E I
2041 AGTCTGTCATCCAGAGCTGTTCGCGAACCAAGAGTTGTTGTCCAAATAGAGAGTGAAATT
447 D I L D D G Y R W R K Y G Q K V V K G N
2101 GACATACTTGATGATGGATACCGCTGGAGGAAGTATGGTCAAAAAGTTGTGAAGGGTAAT
467 P N P R
2161 CCAAACCCTAGgtactgacattttatactgttttaactaaacttcatcttcctatgaatt
2221 tttttcttatttaaaatggatagcactggggtacacatttgatgtttcggagtaaaataa
2281 agtgatatatttgcttttaggatttcagttatctgttttagttttatttactctcttggt
2341 gatacatatttgcatctcaggtacattggagatggtcaaagttggcttgattgatgcaat
471 S Y Y
2401 taaaatgcattctttttgaatatctctctgacttctttttcttcgacagGAGTTATTAC
474 K C T S A G C S V R K H V E R A S H N L
2461 AAATGCACGAGTGCTGGATGTTCAGTGAGAAAACATGTGGAAAGAGCCTCCCACAACTTG
494 K F V I T T Y E G K H N H E V P A A K N
2521 AAATTTGTCATTACAACATATGAGGGAAAACACAATCATGAAGTGCCTGCAGCCAAAAAC
514 S N N L S S G G T S L P Q V T T N A Q P
2581 AGCAATAATCTGAGCTCAGGTGGTACCAGTTTACCTCAGGTTACTACCAATGCACAGCCT
534 A L T L A R N T N R T K P E T Q M Q D Y
2641 GCCCTGACATTAGCCAGAAACACCAACAGGACAAAACCTGAAACACAAATGCAAGATTAT
554 A P G F D R K P V F N N D Y S R L S F P
2701 GCACCTGGCTTTGATAGAAAACCAGTATTTAACAATGACTATTCGAGACTTAGTTTTCCT
574 G H F S N E M K L G A S S I Y P L K F P

2761 GGACATTTCAGTAATGAGATGAAGCTCGGGGCTTCCTCCATCTATCCATTGAAGTTCCCA
594 P L Q H T M P Y S S L E F N R G A T H H
2821 CCCCTTCAACATACCATGCCTTACAGCTCTCTGGAATTCAATCGCGGTGCTACCCATCAC
614 S S S M A P V V P D F Q I S L P S S L H
2881 TCTAGCTCTATGGCTCCAGTTGTTCCAGATTTTCAAATTTCGCTGCCATCAAGTCTGCAT
634 T S A N L S M P G A D F N Y N G R P I S
2941 ACATCTGCAAATCTTTCTATGCCTGGTGCTGACTTTAATTATAATGGGAGACCTATTAGT
654 Q G Q P F L S G Q P F L S G Q Q L V L P
3001 CAAGGTCAGCCTTTTCTGTCAGGCCAGCCTTTCCTGTCAGGCCAGCAGTTGGTCTTGCCC
674 K Q E Q R D D D H F D A G Q A T F E H V
3061 AAGCAAGAACAAAGAGATGATGATCACTTTGATGCTGGCCAGGCCACCTTTGAGCATGTA
694 N G S L S S S V Y Q R I M G N F P S *
3121 AATGGGTCTCTGTCATCATCAGTATATCAACGCATCATGGGAAACTTTCCATCA**TAA**aag
3181 aaggaactttgcttttgaaatactgtcaggttaaaaacaaatggaaaaggaaaaagaaaa
3241 agaaaaga
