# Supplementary material for: Gene Structures, Evolution and Transcriptional Profiling of the WRKY Gene Family in Castor Bean (Ricinus communis L.)
Source: PLoS One. 2016 Feb 5;11(2):e0148243. doi: 10.1371/journal.pone.0148243 (PMC4743969; doi:10.1371/journal.pone.0148243)
Supplement: S2 File — (PDF) [file pone.0148243.s002.pdf]

S2 File. The gene model for *RcWRKY06*. The coding region is marked with uppercase letters, above which is its deduced amino acids. The transcribed untranslated regions, including 5' UTR, intron and 3' UTR sequences, are marked with lowercase letters. The start and stop codons are blacked and the sequences misannotated as the second intron are boxed.

```
   1 aataatccttttattattattatttttctcttcttctattaaccttccttaaattcattt
   1       M A S F T T S S S S S S S C S L N T
  61 cttttcATGGCATCCTTTACTACTTCTTCTTCTTCTTCTTCTTCTTGTAGCTTAAATACT
  19 S I N S Q S S F F S T Q F L M T N P S S
 121 TCTATAAATTCTCAATCCTCCTTTTTCTCAACGCAGTTCCTCATGACTAATCCTTCTTCT
  39 S S L S S F T D L L S S G N I N S K D N
 181 TCTTCATTATCTTCTTTCACTGATCTTCTTTCTTCTGGTAATATTAATTCTAAGGACAAT
  59 N M D N I N V G W G L F D H G N G I D I
 241 AATATGGACAATATTAATGTTGGCTGGGGACTTTTTGACCATGGCAATGGTATTGATATT
  79 P K F K S F P P P S L P L S P P P V S P
 301 CCAAAATTTAAGTCATTTCCTCCTCCTTCTTTACCTCTTTCTCCTCCTCCTGTTTCTCCT
  99 S S Y F A I P P A G L S P T D F L D S P
 361 TCTTCTTATTTTGCTATTCCTCCTGCTGGTTTAAGCCCAACTGACTTCTTGGATTCACCT
 119 V L F S T S N
 421 GTTCTTTTTTCCACTTCTAATgtaagttgcattgtattttactcctgggttttagatttt
 481 aacttgttttcacatgttcttgttactaaatatctatttgtttgtttgtttgtttgtttg
 126       V V P S P T T G T F A G Q T F N W R
 541 tttcagGTTGTTCCATCTCCAACTACTGGGACTTTTGCTGGTCAAACTTTCAACTGGAGG
 144 S N I S N D N Q R G F K G D E K T Y S D
 601 AGTAATATTTCCAACGATAATCAGAGAGGTTTTAAAGGGGATGAGAAAACCTACTCTGAT
 164 F S F Q T Q T R P L L A S S S S S S S L
 661 TTTTCTTTCCAAACCCAAACAAGGCCTCTTCTTGCTTCTTCTTCTTCGTCTTCATCATTA
 184 S S S I F Q S S S S I I S G
 721 TCATCCTCTATCTTTCAATCTTCTTCAAGCATCATTTCTGGGgtatgatttatgattcaa
 781 cagacatcattctgtttcctgtatatactttacttaatttgggtgtttaattgtttattc
 841 cttcttttaaggccaattgtatataaaagaaatgctcttacctttttgtctttatgattt
 901 tgaccctgtttagttgcaaactttgaggtaatgaagtaaagtgctaactgcaaatcattg
 961 caggtcgactagaaatgaagtccacatgtttgctttaggacaaacccgtctggataaatc
1021 aaatataaaaagatactttcttgtttaacatccaaataattttcttgaaaaagaatacaa
1081 gaatcaagagagggagacaagtttcttcgtctttttatcatgctgtgatgagttggggtc
1141 actgatactagtttaaagggttgagataataaattcgaactcaaaaggagccaaattgta
1201 ttacaaagaaaagaaaaaacttctaccaaaaagaattgtataaaagaagcaaaaaatata
1261 tgtaaatcttttctacagatatagtgatgaaaagcaatatgctatctatgtctataattc
1321 attcaattgcatcctagtttgtttttgaatttgttcttgctaacctccctttcattcgac
 198   D E S M K K Q Q E A W N F K K P I K Q A
1381 agGATGAATCGATGAAAAAGCAACAAGAAGCCTGGAATTTTAAGAAACCCATAAAGCAAG
 218   D R P S E K G V V K Q E F T P V Q S F S
1441 CGGATAGACCGTCAGAGAAAGGAGTAGTAAAGCAAGAATTTACACCAGTGCAGAGTTTCT
```

238 S E M A P L Q S S M Q T N A V A P Q P Q

1501 CTTCAGAAATGGCTCCACTTCAGTCAAGCATGCAAACTAATGCGGTTGCTCCTCAACCTC

258 P S Y N H Y S Q P A S Y M R E Q R R S D

1561 AACCGAGTTACAATCATTACAGTCAACCAGCTTCATATATGAGAGAACAGAGAAGGTCAG

278 D G Y N W R K Y G Q K Q V K G S E N P R

1621 ATGATGGGTACAATTGGAGAAAATATGGACAGAAACAGGTAAAAGGAAGTGAAAATCCGC

298 S Y Y K C T Y P N C P T K K K V E R S L

1681 GTAGCTATTACAAGTGTACTTATCCAAATTGCCCAACAAAGAAAAAGGTTGAGAGATCTT

318 D G Q I T E I V Y K G S H N H P K P Q A

1741 TGGATGGACAGATTACGGAAATAGTCTATAAAGGGAGTCATAATCATCCCAAGCCTCAGG

338 R S S S Q L I Q L A A G G T Q E I S D Q

1801 CTAGATCATCCTCCCAATTGATTCAACTTGCTGCAGGAGGCACTCAAGAAATCTCAGATC

358 S F A P V E S V T M Q E D S S L S I G D

1861 AATCTTTTGCTCCAGTGGAATCTGTCACCATGCAGGAGGACTCTTCACTTTCGATTGGAG

378 D E F D Q S S P I S N S G G N E D E N E

1921 ACGATGAATTTGATCAAAGTTCGCCAATTAGTAATTCAGGGGGAAACGAAGATGAAAATG

398 P E A K R F

1981 AACCTGAAGCTAAGAGATTgtaagttgaaaaaagatcacaagactgatgattatgcttta

404 K G Q

2041 tgttctgtatctggattatgaatttaatagtaatctttcatggctttacagCAAGGGACA

407 N E N E S I L A A G S R T V R E P R I V

2101 GAATGAGAATGAGAGTATTTTGGCCGCTGGTAGCAGGACTGTTAGAGAACCAAGAATTGT

427 V Q T T S D I D I L D D G Y R W R K Y G

2161 GGTGCAAACTACAAGTGATATTGACATACTTGACGATGGGTATAGGTGGAGGAAATATGG

447 Q K V V K G N P N P R

2221 ACAGAAAGTAGTCAAGGGAAATCCCAATCCAAGgtaaattagtgatgatattcgatttct

2281 tgctgtttccttgtttgcaagaagtttgtttcatggatttataattgttgcaaatctttt

458 S Y Y K C T S I G C P V R K H V

2341 attctctacagGAGCTACTACAAGTGTACATCTATCGGTTGTCCGGTTCGGAAACATGTT

474 E R A S H D T R A V I T T Y E G K H N H

2401 GAACGGGCATCGCATGATACAAGGGCTGTGATCACCACTTATGAAGGGAAGCACAACCAT

494 D V P A A R G S G Y A S N R L P V N A N

2461 GATGTTCCAGCTGCTCGTGGCAGCGGCTATGCTTCCAATAGACTTCCAGTCAATGCTAAC

514 S S M P I P I R P S V T A N T N Y S N S

2521 AGCAGTATGCCGATTCCTATAAGGCCCTCGGTCACAGCCAACACAAATTACTCAAATTCC

534 L N S T R S S G N Q A P F T L Q M L Q G

2581 CTTAACAGCACAAGATCATCAGGAAATCAAGCACCATTCACCCTGCAAATGTTGCAGGGT

554 T G N I G F S N F G K P S A S Y M N Q T

2641 ACAGGGAATATTGGATTCTCAAATTTTGGAAAGCCAAGTGCATCCTACATGAATCAAACA

574 Q Y T E N V F S G A K E E P K D D S F L

2701 CAGTACACAGAAAATGTGTTCTCAGGGGCAAAGGAAGAGCCAAAGGATGACTCATTTCTC

594 D S F L C *

2761 GACTCATTCCTATGC**TGA**aacgactaactccaaagtagcagttaggattcagggaaaatt

2821 tgttgtggtagatttgcttgtaatagtgtaatgtaggttatagttgcaatttttttcattt

2881 gttttgaggccttttgaagctctatagattaggcctcaatttattacagtacagaataat
2941 tcaacagaaatacaatgaacacttttcttagcaatctcatttgagtctgtagaagttaaa
3001 agaagttcctaaaatcattttcttttcctcttatcttctagattgataataggacttcta
3061 caaagcatttttctttctgatacgacaaaacatctaaatacaggaaacagaagaggatct
3121 tgtttgcatcactctgtattgaagacaagtatttacttacattgaagttaatagtttatt
3181 aattagcaatcaacacttaaaagaatctgaaaccaaaacaagccttggaattaaacataa
3241 gattgagtagataacttgtatcctgaaataagagccgatacagccgacacaaaaaatgca
3301 aagaatgccatgctaatggatgctgcggaggagtctgtaaatatgttatctgcaccttct
3361 cgcatcctgtttgtcataggaactgctgtagaagctgaggatatcaacaaataggataga
3421 agctgcaaccaaacccacaaaaaataatacaacaggaaagtaaatcaataaggcatatta
3481 tcttaaatttcaatttaactcatcatctgttaacagtatgcaataactcaaaagaatgtg
3541 atggtaaggatggaaatttttcagtgcatcctatatatgatataaaactgactcctaaga
3601 aatagcttttatgaatgaatcccatattgaatattctaattcattctggaattcttttta
3661 tatccttaaaaggattggtttctatgagaaataagtcatggttcaaaattgggtccatgt
3721 gcatgctgacacatagatgctctagaagcaaaaggaagtgggaagaacatgaggctaggg
3781 catatcaagtcatatggctcacgcaaattgtcaagtcaaaccagagacgtttcctatgtt
3841 tttcacgacagtatcatttctagaaagtgttaattgctttttctccaagaacagaaagga
3901 tttttctcattgttcaatcaaggaatctccaattccaggctctagaaaaccactaatggc
3961 acttgaatatttgcactctttttctcatccttcaaaatcaaacactttaatcattgaatt
4021 tcacagaaaacctcacatattaatcattttatagataaaactagtacttactttttttcct
4081 accggtcccagatatacatatatattgttttcaaacatacatttaatttgagaaagggtg
4141 tgaagtactgatatttataaactgcccggaccaatatttttgttactgaaataaaaagat
4201 ggcattgacatttttttgattaagcgaattcccaaggttccatgttagttaatatcaatgt
4261 cttctaaattaaaattgcagcaatgaagaattactagactgtctaactaaaaaaagatca
4321 tcttttatattaaagaaaatggaaacgaatcaataatatttcatgaccattcaagtgcca
4381 atcaataaaagtttaatcctttatcttattccaccaattcctcagcaacgtaacagaaag
4441 tttaaaaaaaaaaaaaaaaaagaaagaaagagaaggaaaaagaacaaaagaaggttataa
4501 taaaagaaagaagtctagcctgatcaccaaagaaatctaacatagcagaagtctgtcgct
4561 gaaaaataagttttcctgtagaaagctcatgaacatttcgcagcacttgtcctccagagt
4621 acaaagtcgaaagtattgatattgccaacaagtacctacacccaaatccatcaataaatc
4681 catattcaatcttacagaaaagaaaaatcttgatttgtataatcagacatgaactgacct
4741 gtactcttcatatctatcaaagtttttccaatccccatgtttattactagccatgataat
4801 aaaagcaagcaaagagaacaaaa
