# Supplementary material for: Gene Structures, Evolution and Transcriptional Profiling of the WRKY Gene Family in Castor Bean (Ricinus communis L.)
Source: PLoS One. 2016 Feb 5;11(2):e0148243. doi: 10.1371/journal.pone.0148243 (PMC4743969; doi:10.1371/journal.pone.0148243)
Supplement: S3 File — (PDF) [file pone.0148243.s003.pdf]

S3 File. The gene model for *RcWRKY07*. The coding region is marked with uppercase letters, above which is its deduced amino acids. The transcribed untranslated regions, including 5’ UTR, intron and 3’ UTR sequences, are marked with lowercase letters. The start and stop codons are blacked and the misannotated sequences “gcaa” and “GCAG” are boxed.

```
   1 aatttcaggacagcacggaactaccgcaaaaccagatacatgaaagaaaaggagagagag
  61 gaagaaacaagaaacaaaatcccatttctaacgccataaagatagaagaaagagaattaa
 121 aaccaaaaagcaactgccgtaaactcatgttcttattgtactaacagaaagaaagaaaga
   1                                                     M  A  A
 181 aagtggttttggtgagatacgaaacaaagagaacagtaaggtttctttccaATGGCAGCA
   4 E  K  Q  A  P  P  R  P  T  I  T  L  P  P  R  P  S  M  D  T
 241 GAGAAGCAAGCACCACCACGCCCTACAATAACTCTGCCGCCGAGGCCATCTATGGATACC
  24 L  F  T  G  G  L  S  P  G  P  M  T  L  V  S  S  F  F  S  D
 301 CTTTTCACTGGTGGACTTAGTCCGGGTCCCATGACTCTTGTCTCTTCTTTTTCTCTGAT
  44 N  Y  L  D  S  D  S  R  S  F  S  Q  L  L  A  G  A  M  A  S
 361 AACTACCTTGACTCTGATTCCCGTTCTTTCTCTCAACTTCTCGCTGGTGCTATGGCTTCT
  64 P  I  A  R  P  S  F  F  T  D  A  S  S  N  K  Q  D  G  L  D
 421 CCTATTGCAAGACCTTCCTTTTTCACCGATGCCTCTTCTAACAAGCAAGACGGACTTGAT
  84 N  S  A  N  L  G  F  K  Q  S  R  P  M  N  L  V  V  A  H  H
 481 AATAGTGCCAATTTGGGGTTTAAGCAAAGTAGGCCTATGAATTTAGTAGTTGCTCATCAT
 104 S  P  L  F  T  V  P  P  G  L  S  P  S  G  L  L  N  S  P  G
 541 TCTCCATTGTTTACTGTCCCGCCTGGGTTAAGCCCTTCTGGTTTGTTAAACTCACCTGGC
 124 F  F  S  P  P  Q
 601 TTCTTTTCTCCTCCTCAGgtaattttcttgtttttggatctgaataattcttggttggtt
 661 tttatgtttcgcttggtatggtatttggtgccttctttagcaattcggtggtgttagttt
 721 gcgattggatggttagtgttttgattgcttatgagatttggatggattgagttttttat
 781 tttttctttttgctttgttatgatcatagaaaaagatttgacctttttcctttttttttt
 841 ttgttgatatttattgtcaattgaaatagtttgcagagtttgagagcgagaagggtgatt
 901 tgggtgctccttgttggaatttggatgaaataataggctggattttagatgagcagaatt
 961 ttcggtgcttgttgattatgcaattgagaaagtgataacatttatgagcttaaatattag
1021 ctcaaatatagtggggtttatatgttattatgctcagtcgtgagcaaattaattcgacaa
1081 ttgaaagaaaactgaaattctttttccttgtaacacgagggagtgaggaaccctaactt
1141 gcaaacctttttgcctatcttctggtgagttctgcattggaaccattttatacctagtcta
1201 caacctagcaaattatcagtttcccccaggtcaggattggattacagcttcattatatag
1261 caaggatgatttatttgttgttcttggataaaaagaagaaatttgattaacacagattat
1321 ccgaagtgttggaatgtgactggtcaggctgacagcaaatggagtttggtttaataattt
1381 gattaaacaaaccttccagccgtctaattcgttgactcaaagccttgcgatgctcatgct
1441 tatggttaaggtagtgcaataaattgacagtgcaacaataatgtgcctggaaacacatgt
1501 ctttcttaggggagtaaactcatttgtggtctgtaaatttaattcatgtttcctattatg
1561 ggtcttatgttttaatttctatcatacttttattttaattttagtacactaaattccttt
1621 ttgcatgattccttgagaaaattcaccagagatatgatgacatcatagcttgacatggat
1681 atagtattttataattttgaaaatttcatggtggtatgatgacacatcctatttcagcag
1741 gatttcttttggaatcaatttacaagatacatagatttttgtgatacaaatcaacttgta
```

1801 aggattgtaatgggaaacacggttaaaattagggttgtaaatgagatttacccctacctg

1861 gagaaatgtaattcttttctgggtcctggaaagatgtaacttatgtttttgttttgtttt

1921 tccttaacttagttgaattctattaatattataattgtgcctgataaagagatgggcttc

1981 caacctaattaaaatgaacgaatacttgagaggaaacactctattctggcaacatttggt

2041 gtgatggactgatgctgcttggaaccatattattgagcaatgacttgaaactggaagtga

130 S P F G M S H Q Q A L

2101 gacatttttttctatattataattgcagAGTCCTTTTGGAATGTCCCACCAGCAAGCCTT

141 A Q V T A Q A A L A A Q N H M H L Q A Q

2161 GGCACAGGTTACAGCCCAAGCTGCACTAGCTGCCCAAAATCATATGCACTTGCAAGCGCA

161 Y Q P A P V S A S T E L L T R Q P S F T

2221 GTATCAACCTGCTCCAGTATCAGCTTCCACAGAGTTATTGACTCGGCAGCCCTCCTTCAC

181 P G E A S Q L Q M L P S T S E P H N S M

2281 TCCTGGTGAAGCTTCACAGCTACAGATGCTTCCCTCAACGTCTGAACCTCATAATTCCAT

201 V E A S D L S H S D R K Q Q P P L A V D

2341 GGTGGAAGCATCAGACCTCTCTCATTCTGATAGGAAACAACAACCTCCTCTTGCTGTTGA

221 K P S D D G Y N W R K Y G Q K P I K G S

2401 TAAACCGAGTGATGATGGCTACAACTGGCGCAAATATGGGCAGAAGCCAATTAAGGGCAG

241 E Y P R S Y Y K C T H L N C P V K K K V

2461 CGAATATCCGCGGAGTTATTACAAATGTACACATCTGAATTGTCCTGTCAAAAAAAAGGT

261 E R S S D G Q I T E I I Y K G L H S H E

2521 TGAGCGTTCTAGTGATGGCCAAATAACTGAGATCATCTATAAAGGCTTGCACAGTCATGA

281 Q P Q P N K R A K D S S D Q N G S T S S

2581 GCAGCCTCAGCCTAATAAACGTGCAAAAGATAGTAGTGACCAAAATGGAAGTACAAGTTC

301 Q A K P E P G S L S Q A G N I N K S N E

2641 TCAGGCTAAGCCTGAACCTGGTTCCCTATCTCAAGCTGGTAATATAAACAAATCGAATGA

321 T F P A H S V H G M E Q E P T Q A N T E

2701 AACCTTTCCTGCTCATTCAGTGCATGGGATGGAGCAGGAACCTACTCAAGCGAACACAGA

341 L P G S S D S E E A G E M R A E D G N E

2761 ACTACCTGGGTCAAGTGACAGTGAGGAAGCAGGTGAAATGAGAGCAGAAGATGGAAACGA

361 D E P N P K R R

2821 GGATGAACCAAACCCGAAAAGAAGgcaagttaggaatttttcagtaaatgtagtcataac

369 Q T D V

2881 tggcttttcccccccttattttctgaaaatatctaaccccggagtgcagGCAGACAGATGT

373 G T S E V A L P H K T V T E P K I I V Q

2941 TGGGACATCTGAGGTTGCTTTACCACACAAGACAGTCACAGAACCAAAAATCATTGTGCA

393 T R S E V D L L D D G Y R W R K Y G Q K

3001 AACAAGAAGTGAAGTTGATCTATTAGATGATGGCTACAGGTGGCGCAAGTATGGCCAGAA

413 L V K G N P H P R

3061 GCTGGTGAAAGGGAATCCTCATCCAAGgtgatcaattgattattgctttataggtctttt

3121 gcctctagtgtgtttttgatgtctaggtggattttagggagtttcctaaagataatactt

3181 gcaattacaatgtcaatcacagaaatccatctttattgccttcactatgaagtctgaaga

3241 gtttctagtaggagaaacaaaatacaaccgtctacaaaaaattccagagtttttaccttat

3301 caaatgacctgtcatatgatgccaagaattattgttttgaaagtttgcatgagaatcccg

3361 cgcaagatgaccccgagtttgccacttttgaaattacccctataatgagtacaataatga

3421 aaagtttcctcgaagactcaaaatgtcctataccttcattcagaaccctccccttgccta

3481 gagatttaattgtgcagtttccccataccctctttaaatatgagagccattagctcaaca

3541 ctatacccatagccatgatcttatgtttttttgtccatctgctcttcttgacacttgtcat

3601 tacttgccaaatgtattcttgcatgtctgtaaaatggcttaaatatcttaccttttagaa

3661 tctgttcattatgaaaattcttctctttaaatctgtaagacctttcttgcaatcgacggc

3721 atcttctaagagagttttccatccactttatcatcatcatcattattattgtttttctta

3781 aatattaatgattattattattatcattatctcaattacctagtttgcagtgaatttttt

3841 tttttggttatagctattgctttctatttagggctatatttgccactaaatcagggctat

3901 tgttgttttcttatattcttggccaacatcctattttctcccctttcttggaaatatata

3961 tatatatatatatatctgtgtatttgtatttagcttatctttttttgcttttggtgtataa

4021 gacaaattacttcacatttgattctcctctttctagcatctatgccatatttgagtatgg

4081 ttattatttttgaatcacatgcaactggtggtgaaatctctagtatttgctagtgatttg

4141 gcaaggaataacttatagtttcatgccgtctatatatatatatggttaacttttatgtgc

4201 ggggtttgaccgtttgccctttgagacattaatcctatattcatttcctggttggtgtcg

4261 gactaaatagaattgtttaaattttgatatcagtctctttgaaacttgataaccaatgac

4321 aatgatatcagagagggagagagagagagagacagagttttgccctaatattttgccata

4381 tatcaagattgttttataatctttctatatcctggcaatgatatgcctattctctggttc

4441 tggaacatctgtcaaatttaagttcctattaagcttccaatattttggttattctatcat

422 S Y

4501 agatattgcttgctttctgggagtttcatttctaatgtacttattatatgaatagGAGCT

424 Y K C T S A G C N V R K H V E R A A A D

4561 ATTACAAATGCACTAGTGCTGGGTGCAATGTCCGTAAGCATGTTGAGAGAGCTGCAGCAG

444 P K A V V T T Y E G K H N H D V P A A R

4621 ACCCTAAAGCTGTGGTAACCACATATGAGGGAAAACATAATCATGATGTTCCTGCAGCTA

464 N S S H N T A N N S A L Q L K P Q K V V

4681 GAAACAGTAGCCATAACACAGCCAACAACAGTGCACTACAATTGAAACCTCAAAAGGTGG

484 A E K H P F L F G N N D K R P V L L Q L

4741 TGGCTGAGAAGCATCCTTTCCTCTTTGGGAACAATGACAAAAGACCAGTACTTCTACAAC

504 K E E E I A V *

4801 TGAAAGAAGAGGAAATCGCAGTA**TAA**atgctctgcttaaaactgctagagaactataagg

4861 atgactccagccaactaacctggtagaatgaagaagagaggaaagtcttttgaaagttgg

4921 gagaccttggtggttttggttctttttcattttggtgctttttttcttttttttttttttc

4981 ttttttgtttgttttacctttttgaatgaaatgtagttgtaattgttgctgctgcacatg

5041 ttaacaatttgatacataacattatttcactacagttatgtaaaattaaatttagaag
