# Supplementary material for: Gene Structures, Evolution and Transcriptional Profiling of the WRKY Gene Family in Castor Bean (Ricinus communis L.)
Source: PLoS One. 2016 Feb 5;11(2):e0148243. doi: 10.1371/journal.pone.0148243 (PMC4743969; doi:10.1371/journal.pone.0148243)
Supplement: S4 File — (PDF) [file pone.0148243.s004.pdf]

S4 File. The gene model for *RcWRKY08*. The coding region is marked with uppercase letters, above which is its deduced amino acids. The transcribed untranslated regions, including 5' UTR, intron and 3' UTR sequences, are marked with lowercase letters. The start and stop codons are blacked and the sequence of the first exon that was not annotated previously is boxed.

1 M S E N E T H H S P P
1 catagagggacaaaacatattatcttta**ATG**TCCGAAAACGAAACCCACCACTCTCCTCC
12 P A P S P A S T T S A S S A S A K S S T
61 TCCAGCGCCGTCGCCGGCATCCACCACCTCCGCGTCTTCAGCATCAGCAAAATCCTCCAC
32 S S R P T T I T L P P R S F N E T F F S
121 GTCCTCACGCCCAACCACAATTACTCTCCCGCCACGCTCCTTCAACGAAACATTCTTCTC
52 T P T P N M G L G L G F S P G P M T L V
181 CACTCCTACTCCAAATATGGGTTTGGGCTTGGGCTTTAGTCCTGGGCCCATGACTCTTGT
72 S S F F S D S D D F K S F S Q L L A G A
241 CTCCAGCTTCTTCTCTGATTCCGATGACTTCAAATCCTTCTCTCAGCTTCTCGCCGGCGC
92 M A S P A A V K A P P F P P A Q E D K I
301 TATGGCTTCGCCGGCAGCTGTCAAAGCTCCTCCCTTTCCTCCTGCTCAAGAAGATAAAAT
112 S A I G V T R T P A N L S I V P P P P T
361 CTCCGCCATAGGGGTCACTAGGACGCCGGCGAATTTGTCGATCGTTCCGCCGCCGCCGAC
132 F S P V A L L D S P G F G F F S P Q
421 GTTCTCTCCGGTGGCTCTTCTTGATTCACCTGGGTTTGGGTTCTTTTCTCCTCAGgtagt
481 tgataaattgaaaagggttgcctggtgttgttaatagtttttgttttgtttagttcatta
541 aatttctgaaagttctttttttttatatttttccaaaaagtttttaagcaatttaaataaa
601 acctactttttcttttctttaatgcaattaaggtagatggaggatgattttgatgaacaa
661 atgattgaacttaatgcccataagtccagctgcttaagagtggagtaataagagagaaat
721 tttacagtaattttgagtatattaatggaagagtattttagtcattttaagttatttttc
781 actgtaattgagtctatttagaatggaggcagtaagactttgaccaggtatacgtctgtt
841 tcatgcttttagttttagagaataaatcacgaaccagttccatttgttaagttttggcaa
901 aatgcctaaatggtccagccatgttgctccaatgccttgatttttgtttttttgttttttt
961 accaaaatgtccaatgaagggctgtgcttggaacttgacatttccattttgttacatgct
1021 tatgtttttgagattaagccattgggctagtagctattttcctcattgatataatattag
1081 gaagttgaaccccttgcattacccttccctttgatatagagaaaatgttgggacattgtt
1141 ctgcctagatgtatatcaataacgagaaatgaacagcttgatttattcttcctttatatc
1201 tcttgtttgtcatcacataacatatgaacccttttttgtttttctagaatcgtgtggctgc
1261 atttgtctttctgaatgttcccacatggtcttctctgtgctatgcttatcaaccttataa
1321 gagcactctgttctcactgataattggcaccattttgtttcataaaggccaatgtttggt
1381 ctcctttatagtgattactgaaaggcgtgtaggagaacagtgcaagtatgtgaagagtag
1441 ctatattagcagctcataaatgcaccatctttgtgcttattgtttgaaccaaatacattt
1501 taagcttatagatatataacagatttggagctgttattaatgttctcgtgatatgtcact
1561 aatgtgtctttattcacaatcattttcttttaaacttattttaatcatctgtgaggtctg
150 G P F G M T H Q Q A L A Q V T A Q A A
1621 **tag**GGGCCTTTTGGAATGACACACCAGCAAGCCCTAGCCCAGGTTACTGCTCAGGCTGCA
169 E A N S H I H I Q A Q Y S S A P A T S S

1681 GAAGCCAATTCCCATATACATATTCAAGCACAATATTCATCAGCACCAGCAACATCTTCG
189 T Q F S S I S T N S T I H Q Q M P S S I
1741 ACCCAATTTTCATCCATCAGCACTAATTCAACCATTCACCAGCAGATGCCTTCATCAATA
209 P D T N A S E K E L S D F S F P D Q R A
1801 CCAGACACTAATGCATCTGAGAAGGAATTGTCAGACTTCTCGTTTCCTGATCAGAGAGCA
229 Q A S S V T V D K P A D D G Y N W R K Y
1861 CAAGCTTCTTCAGTCACTGTTGATAAACCTGCTGATGATGGCTACAATTGGCGAAAATAT
249 G Q K Q V K G S E F P R S Y Y K C T H P
1921 GGGCAGAAGCAAGTGAAGGGCAGTGAATTTCCTCGAAGTTATTACAAATGTACACATCCT
269 S C P V K K K V E R S L D G Q V T E I I
1981 AGTTGTCCTGTCAAGAAAAAGGTTGAGCGATCTCTTGATGGTCAAGTAACCGAAATTATC
289 Y K G Q H N H H P P L P N K R G K D A G
2041 TATAAAGGACAGCACAACCATCATCCTCCTCTACCCAATAAGCGTGGGAAGGATGCTGGA
309 N M N G N Q N N Q T D S G L A S Q F Q I
2101 AATATGAATGGAAATCAGAATAATCAAACTGATTCAGGACTAGCTTCCCAATTTCAAATT
329 G N V N K L K D R K D Q E S S Q A M P E
2161 GGAAATGTGAACAAATTGAAAGATAGGAAGGATCAAGAATCAAGCCAGGCTATGCCTGAA
349 H L S G T S D S E E V G D T E T G I D E
2221 CATTTATCTGGGACAAGCGACAGCGAGGAAGTCGGTGATACTGAAACTGGTATAGATGAA
369 N D E D E P E A K R R
2281 AATGATGAAGATGAACCCGAGGCTAAGAGAAGgtacttttgcaactaagttcttatttga
2341 tcatacttaaatgatctgtattttaaatatattacatgtctaacaaaatctgtgttttgc
380 N T E V R V T E P V S S H R T V T E P
2401 agAAATACAGAAGTCAGGGTAACAGAGCCAGTTTCTTCACATAGGACTGTTACAGAGCCT
399 R I I V Q T T S E V D L L D D G Y R W R
2461 AGAATCATTGTACAGACGACCAGTGAGGTTGATCTTTTGGATGATGGCTATAGATGGCGC
419 K Y G Q K I V K G N P Y P R
2521 AAATATGGACAGAAGATTGTCAAAGGCAATCCTTATCCAAGgtggttactatcagcctat
2581 ctgattatgacttggagcagtcattataataaaacaaacattgttcttaggttctgataa
2641 cattcattatcaatttaaaattccttcttgtagtctatctagctcaaattgataggagtt
2701 cttcagttcttagatatttatgcattttgctttgcagaggccaagggcctcttactctct
2761 ctgccatgggggatgcgggtggagttataaaattaaagaagatgaatatattatgccatc
2821 agcaacaattattccttttaggcatagaattattgtctggaactgtttgttaaattaggt
2881 acacagaaatgttaacccatatatatatttgtagtgtgaccattgtggtcattataatct
2941 caaatgcaaaatggtttgaaatcctgtcctcttattgtagtgccaaaatagcctgcatta
3001 agaaaaaacatttgatagaaagttggtcttaagaacttcagtgggagaaaacattgtggt
3061 taaagttgggacatatccttcctcttgctcttggtgctctttgataatcttttgcaggat
3121 ttttaagttcatatttttgctctctcagtctttctttcctctgcttttagaaaacgtcga
3181 tgcttttgctgatatttgattatttattgtgttttgctagtcaatagtgaaaattaaact
3241 attcttttgttgaacttttaatatcttttctacatgtctaattatttattatttcctttg
3301 gtggaggctttctcatcattgccatttctcatcaattcatgtattcatagagaaaaggtc
3361 aatggtgacatctctaacactttgctagtattatctcgaatgcttgggttctcattgctt
3421 attctattgatagaatgtatacatgttgcaatacctagttttatcaaaacaaatctagga
3481 cccatgcatatcgattttccattctgttcagtctcccaaacatgcatgcttgctctatgg

433 S Y Y K
3541 atttgatgttgacgtttaatttgaggtttccttcaatgatctacagGAGCTACTATAAA
437 C T T V G C K V R K H V E R A A T D P R
3601 TGCACAACTGTAGGCTGCAAAGTCCGTAAACATGTTGAGAGAGCTGCAACAGATCCAAGA
457 A V V T T Y E G K H N H D V P A A K G S
3661 GCAGTTGTAACAACATATGAAGGAAAACATAATCATGATGTACCAGCGGCAAAAGGGAGC
477 S H N I A H S N S S E L K Q Q N V E K N
3721 AGCCATAATATAGCCCATAGTAATTCATCTGAGCTAAAACAACAAAATGTGGAGAAGAAT
497 A V N N R R R D S S T N N R P P I A R L
3781 GCTGTAAATAACAGAAGAAGGGACTCGAGCACCAATAATCGGCCACCTATAGCGCGGTTG
517 R L K E E Q I T *
3841 CGATTAAAGGAAGAGCAGATAACT**TAG**tttctttaagttatcacaaaaagtaggatgccg
3901 cagacatttactatgaatcaggaaaagctgtaagatatcttcgctgaaatctgtttttca
3961 tgctctttgaggccattgttttcgtcaaggcccatgtcacaaactgccatattcaaaagg
4021 aagaaaaaatcataaccacaagatagaaggaagtcaatctgaggcaaactttctcagatt
4081 gatctacagggcatgcgatattgttgtatttacacagtttagtaaatatatttcatttgg
4141 aaatctgttcttgtgcattcttttgtacattatctggaagaaatgttgcaaatagctgtt
4201 atacagttgttacatttggattttatgtttgaaagtgcagatatttttctactaa
