# Supplementary material for: Gene Structures, Evolution and Transcriptional Profiling of the WRKY Gene Family in Castor Bean (Ricinus communis L.)
Source: PLoS One. 2016 Feb 5;11(2):e0148243. doi: 10.1371/journal.pone.0148243 (PMC4743969; doi:10.1371/journal.pone.0148243)
Supplement: S5 File — (PDF) [file pone.0148243.s005.pdf]

S5 File. The gene model for *RcWRKY10*. The coding region is marked with uppercase letters, above which is its deduced amino acids. The transcribed untranslated regions, including 5' UTR, intron and 3' UTR sequences, are marked with lowercase letters. The start and stop codons are blacked and the misannotated sequences are boxed.

```
   1 cacatataccccccaaccccacctttgtctttttctctctctttaaaaaagaaaccgatta
  61 cgatacacaaaaattcgattccttctaaaaggaaaaaaaaaaagccggatataaacgtcg
 121 gtatatcggcatcggtttagattttatcgggtttgagtttcggacactataaaattaaaa
   1                                                   M  A  E  S
 181 ttgaaaaggcaactgttttgaaactttactctctgagagagaattggcgATGGCAGAGAG
   5  K  S  V  E  A  L  Q  V  Q  H  K  R  Q  V  D  E  H  N  D  H
 241 CAAGAGCGTAGAAGCTCTCCAAGTACAACATAAACGACAAGTTGATGAACACAACGACCA
  25  V  L  E  E  Q  E  E  D  D  D  D  E  E  E  D  S  Q  L  S  E
 301 CGTTTTAGAAGAACAAGAAGAAGATGATGATGATGAGGAGGAGGATAGTCAACTCAGCGA
  45  L  D  Q  L  G  E  S  Q  Q  P  E  E  L  S  S  G  P  V  E  L
 361 GTTAGATCAACTCGGCGAGTCGCAGCAACCCGAGGAGTTATCTAGCGGTCCAGTAGAATT
  65  E  A  L  V  A  V  P  T  S  T  A  I  L  E  N  K  Q  C  V  G
 421 GGAAGCTTTAGTGGCGGTTCCGACTTCCACGGCGATATTGGAGAACAAGCAGTGTGTAGg
 481 taggtttttcaaaaagaattgcagagtgttcctttggaaatttaatttaatttaatattg
 541 ttatagaaaaaaaagagaagttattgtttgtttggtgagagtggaagtgtctgatctagc
  85     L  E  D  N  A  G  L  E  K  H  V  D  D  S  E  F  K
 601 agGTTTGGAGGATAATGCAGGTTTGGAAAAGCACGTGGATGATTCTGAATTCAAGgttag
 661 agaccaaaagtttttattattattatatttattattatcattcatttctggtctttgctt
 721 tgtttattcattatttaatttagttatttattacttttagtagttgtgttcttttgtgat
 781 tttatgtctgtgcctgtttgaatctatttgaatttagtgtttagtatgcaaatgatttgt
 841 ctgcgaaatgcaaatctttagagaattgatcttctgctagtagttgcatttgctgacaga
 901 tctttatttttacgttattttactttattcatttttaatttgagattgaagtgattctga
 961 accgtgttttggtattcattatgatatttcagttatatacttaatcaacaacatcagaaa
1021 tgtgccgactaaaggataatttgctaggaatggacttaagtttgtaatttaagatgcttt
1081 atgtgctctccaaaagttaatattaacagtagacattgacacttctgggtttttagtcga
1141 tcctataaagaaaatatcttaaatgctttgtctacgcggtacatgtgaatttttttcttaa
1201 agaaaagcataaatgtccatgaactgtagttgaagggtgagatcatggctaacctctaga
1261 aaacctaagattaaggaaaaacgttttggaactgttggaaactattactgctttttaaac
1321 ttttgacaatacagtaaaagtgttccaaccgttttctatgcattgaactataagggcaat
1381 agcttttgaattgtatttgaatgaacaattgaatcaactgttttgaaggttgataaactt
1441 gtggaaatgattagacttgaatgtggaagaaaacaaaaggtgttgacatgctaggagttt
1501 ctataatcattgaccaatccttgcatttgaatgagtgaataaattattgctagtagtatt
1561 gatctaattctctaaacctttaaggatgaaagcatgcatatcaaaaacattaataaagaa
1621 tttaaaattaataatagttaaaaggattcatgcctcttcctgcttctctttggagtagca
1681 ttggcggatgtatccttgaaggtccttctatgggtttttttctttaaaagggtaaaacttg
1741 aactgagtgatgcataacaatgttatcatcatgcattatatcctctcatcttttgtctaa
1801 gcatctaataaagatatgcacaaaaatcaaagcacaaaggttcactgcattgaaaaattg
1861 aaaaaataaaaagaaatatatcttcacttgtgattctttcttatgcaaaattatgggagc
```

1921 caaatgtcctaggtattcttctcttgcattcttgtcgtcctactttaatgtcaatttagc
1981 cataaatgattttccataaagggttagaaagcttatataatattagcatactctctcttg
2041 aagattgccaaaatatatttttttacctaggattgttagggagctttattgctctgactgt
2101 tacactttacctcaatgaacctgtgttatacacgtaatttacgcttgtaagagaactaaa
2161 atctcatttttcagaaattagagaccagagtatcacacctaactttaagggttccattaa
2221 aagcttcaaataaaaaaaagaaatcgaagggtgatgatccgtacagttgacctcaagcag
2281 ttctagattagagcttatttaagttgagcagtcagtctgtaagctgtaactaaaccattg
2341 tcctttagtcaagtgaggagttgttattcctactatttcagaacttaggtggctctttat
2401 ctcaatcagcatcctgagctatgacaatgctttgcagcagcctgcagtaaatatggaaag
2461 aaggtttcagttggtgcatgtatggttaccaattatcgacatctgagttgatacagttta
2521 agttagaaacatttgcttaactttaagatagaatagggttagtatgttgtttagttttga
2581 taaaatattactaggacagtctcttgttttgactttactatgaatatgaatgttctttta
2641 gtactttggcgacctagctagaacaatttacatttcttattggcaaaattcctcttcttg
2701 tatttcctttgtgcctgctagttttaaaacatttaaacattaatgtgccagattttgtgt
2761 agtggtgtttgtgagacgtggactcaccctctgacaagccaacagctgcaatctatgctt
2821 tctaaattgcactctgaactctggatgcatctgtaattctagatactgtgcattttaaat
102 E Q D G I S H P E V S G D
2881 tttgttaaaattctttaatcagGAGCAAGATGGGATTTCACATCCAGAAGTTTCAGGAGA
115 V T S Q C A E A Q T Q N Q L Q S S V C P
2941 TGTAACCAGCCAATGTGCTGAAGCTCAGACCCAAAATCAACTTCAGTCTTCTGTTTGTCC
135 T S L S E L S P T S V T Q P I S S A P S
3001 AACTTCTTTGTCAGAACTTTCTCCAACTTCTGTTACACAACCAATATCATCTGCTCCAAG
155 P T L P E P R V S P S K F N N Y N A C I
3061 TCCAACTCTACCAGAACCAAGAGTGTCCCCATCGAAGTTTAATAATTATAATGCCTGCAT
175 P E A D Q Q N S S E L K S L S L P I V K
3121 ACCAGAAGCAGACCAACAAAACTCTTCTGAACTGAAAAGTCTTTCTCTTCCCATTGTGAA
195 A H V L D G Y N W R K Y G Q K Q V K S P
3181 AGCACATGTTCTTGATGGTTACAACTGGCGAAAATATGGTCAGAAGCAAGTGAAGAGTCC
215 K G S R S Y Y K C T Y S D C C A K K I E
3241 TAAAGGTTCTCGAAGCTATTACAAGTGCACATATTCTGACTGTTGTGCTAAAAAGATTGA
235 C A D H S G H V I E I V N K G T H S H D
3301 ATGTGCTGATCATTCGGGCCATGTAATAGAGATTGTTAATAAAGGAACACACAGTCATGA
255 P P R K N N S T R G S K V A L L S A P V
3361 TCCACCTCGAAAGAATAATAGCACAAGGGGAAGCAAGGTTGCTTTATTATCTGCCCCTGT
275 L E N S M K E H S M G M H T D S S Q S T
3421 TCTGGAAAATAGCATGAAAGAACATTCCATGGGCATGCATACGGATTCAAGTCAGTCCAC
295 L F K D S I Q E T P N I S E K K R Q N S
3481 GTTGTTTAAAGATTCTATACAGGAAACACCAAATATTTCTGAAAAAAAGCGGCAGAATTC
315 S G S D G N G K I L I K E E H V S E P E
3541 AAGTGGCTCTGACGGGAATGGGAAAATTCTTATCAAAGAGGAGCATGTCAGTGAACCTGA
335 P K R R
3601 ACCAAAAAGAAGgtaaggtgtccctttttctttgttctgctgaattgaatttttcatctgt
3661 gatgttggcactttctgatataactgtaaaaaggcagctttggatattagttgattacag
339 M K K E N L E C S G T L

3721 tatttttctgcttatttatccagAATGAAGAAGGAGAATTTAGAATGTTCAGGAACTCTA
351 L K P G K K P K F V V H A A G D V G I S
3781 TTAAAACCTGGAAAGAAGCCTAAATTTGTGGTCCATGCAGCGGGTGATGTGGGAATTTCA
371 S D G Y R W R K Y G Q K M V K G N P H P
3841 AGTGATGGATACAGATGGCGCAAGTATGGACAAAAGATGGTGAAAGGAAACCCCCATCCC
391 R
3901 AGgtgcatgtttatttctttatttgttctttgcttgcatggggattttgacctggtatac
3961 taatacaaattaacatcagataatgcagtttagatgccatattaactatatatatatgca
4021 aagtgtagtttcttggtttgtttctatttatcttatcatagtggaagaaagtatcataac
4081 gcggaaattgatttattattctcatttttttaatgctcatgctactatggctctttctt
4141 tgtgtgcaaactcatgctattagagatcatgttctctggatggagtatttttattactct
4201 gttgctttagattttggcaaaagcatttgaaatatgaatcagtctaaaatatgcaacttc
4261 tttcttcgttataatattttgggagttgtgtcatgttccagttacaaagaccaaaatatt
4321 taggtgaaactgctttgtttctctggagagtatttgggaaaggagtggactaaaatgggg
4381 gtctttgagattttgtgcgaattgtcatgttcagttacaaagagtttgtgtaccacaaat
4441 gctatgtcttttgaaggacattcactctaacagaccagtttcaaatgaatgttgatgcaa
4501 gtcctataagggacatggcttcctatgaattatggttggtgacgagtggagaataatggc
4561 atttttgatagataacttatagttggtcttctggcttctggatgagggtacaactgaagc
4621 atttctatgaggtggcttatagggaagaattgtatctcggatagtctcttgaagaatagc
4681 ttttcaaatggatgtttaccagtgtcatacaacaaagccaagtcactcatgatttttgga
4741 tgatgagcagtggagatagtattttctcccttttcattgtaaacctttgcacattactct
4801 tctcgtcctctgatgggtagcagagttcgttaaaacatttaatattccaacttttgaggt
4861 caaatgcaggagtgttggccatatggttctgcaatgatgatacaagagtaggtagataaa
4921 aacaccttcttttccctttgtttcttcataaatttaggaaaattcttaatttctat
4981 gataaagggaaagttcttttttcttattagaattcctgcatccctgatagaatctcaac
5041 atatttgaattgaggaagtagcatccaaggtcataatctatgtagtagctctgattattc
5101 atttctcggtccgagttagatcttccaaattcaatccagcctgtggattctgttcttttg
392 N Y Y R C T S A G
5161 tgctgatttctcactctaaacaaattgtgcagGAACTATTACAGGTGCACTTCTGCTGGA
401 C P V R K H I E T A V D N T D A V I I T
5221 TGTCCAGTCCGTAAGCATATTGAAACAGCGGTGGACAACACAGATGCGGTCATTATAACA
421 Y K G V H D H D M P V P K K R H G P P S
5281 TACAAGGGAGTACATGACCATGACATGCCTGTACCCAAAAAGCGACATGGTCCGCCTAGT
441 A P L V A A A A P A S M S N L Q L K K T
5341 GCTCCCCTTGTAGCTGCTGCTGCTCCTGCTTCTATGAGCAATTTACAGCTCAAGAAAACT
461 D T L P N Q I S S T Q W S V G K E G E L
5401 GATACATTACCGAACCAAATAAGTTCAACCCAATGGTCAGTGGGGAAGGAAGGTGAATTA
481 T S E T L D L G G E K E K A I E S A R T
5461 ACTAGTGAAACCTTAGACCTTGGAGGCGAGAAGGAGAAGGCCATCGAATCAGCTAGAACT
501 L L S I G F E I K P C *
5521 CTTCTAAGCATTGGATTTGAAATCAAGCCTTGC**TGA**taggtatcttttcctgactatggc
5581 ataaactaaaagattctgttcttgctggaaaatcacgtagttgtctagatgtgaattatc
5641 attaaattattaggtctgattgatgatggttttcctttttctcttctcctttcttatatt
5701 ctgcttacccttttccttccttttcttcaaatttccttcttccttcagccttctccactc

5761 tatcatggaacagttgtatatttggttattgtacgcagcaggaagaccttgtgagatatg
5821 gtaagctaatagtattggaaggtcggcagtcggcaccaatttttatttttttttcctcat
5881 cttggttgggacccatttgtttcaagatctccattcaggattagtgtctgtcatttttgc
5941 tgcggatctagaattatttagaatgggtagttggcagctttaaaggtgggcctttgttga
6001 gtgatgaaattggagcacggggagcaggaaacctgtcaaatatcagatctaaagaacttc
6061 cagtgtcccagtgtcaaaaaggaaaaagaaaactgtaataaaaattaatgtaaggtggaa
6121 atcccaactgttcaaattggtcataagattataattagtcccaagtagttc
