# Supplementary material for: Gene Structures, Evolution and Transcriptional Profiling of the WRKY Gene Family in Castor Bean (Ricinus communis L.)
Source: PLoS One. 2016 Feb 5;11(2):e0148243. doi: 10.1371/journal.pone.0148243 (PMC4743969; doi:10.1371/journal.pone.0148243)
Supplement: S6 File — (PDF) [file pone.0148243.s006.pdf]

S6 File. The gene model for *RcWRKY11*. The coding region is marked with uppercase letters, above which is its deduced amino acids. The transcribed untranslated regions, including 5' UTR, intron and 3' UTR sequences, are marked with lowercase letters. The start and stop codons are blacked and the misannotated sequences are boxed.

1 caaaggcataaaaattaacaaaagccctaaaacattgaaaaaaaagaagaaaaaaccctc

61 gaaagaattaaatagtttgatgatgatgatgatgttagatggtcaaaggcaacaaaaact

121 gcccggtcaataataagtcaaaaagacagcaaaagggtcccataatataccattccctct

181 ctgtttcggtgtctcttaatcacctttctttgtttctcaaatcttctcttctcttctctc

241 cataaccgaacagaggacaggactctccaattcaaacaaacccaacagctttttgcacta

301 aatactcgcaataacactcttttgtaccctctctcttttctctctgttaaggcttgaagg

1 M C D S S M A D S A S F S S L L K S V

361 ggtc**ATG**TGTGACTCATCAATGGCCGATTCAGCTTCCTTCTCCAGCCTACTCAAAAGCGT

20 L T N N S S T V R F Q

421 TTTGACTAACAACTCTTCAACTGTTAGATTCCAGgttcctttctgcttttgtttttatgc

481 ttgttttgtatatattttttttaatttatcttatttgaatgggactttacttcgctttcg

541 ctttgttgttgtctcatgaattttctctttctcaatttggtttcttgagttgatcttttc

601 tgaatttgttacttatgcagttcgagtttttttctttgctatttggtacactttagccac

661 tgaatcacaacaagttcactgggatctttttttttttctttctttcccactatcgttagga

721 aatgaatgagcttagtttttttatactatgtcctgtaccgtttttgtctaaattactcatt

781 ttttggaagatcaggtaatcagttttcataaaatttcaaatacttttaaaattttataat

841 ggatcatttaatttgacgttataatttgctttatgagtatgcagccacgatttgttttag

901 ttaattattttggaaaagaatttcaaagtataggaattcgcaagaaactgaaaacatgct

961 gctttcatgaataatcaaatggcctgatatagattattcttcttggttggcaaggtatcc

31 G

1021 ttctgccttgaacttctttagagttgttttatgagaattatgtgatatctatgatgtagG

32 Q Y N T S Q Q E I M V S T K P R V S E A

1081 GGCAATACAATACATCCCAACAAGAAATTATGGTGTCAACAAAGCCTAGAGTGTCTGAGG

52 Q F E L P A E C T N P S T N I S S L A P

1141 CACAGTTCGAACTTCCGGCTGAATGTACGAACCCTTCTACCAATATATCATCTCTTGCTC

72 A T I S I S H S A P V I Q K H L L T E N

1201 CAGCTACAATATCAATCTCCCATTCAGCACCTGTAATACAAAAACATTTGTTGACGGAAA

92 I D R I T S H P R I V M E S P A T D G Y

1261 ACATTGACCGCATTACTAGCCATCCCCGTATTGTTATGGAGTCACCTGCCACTGATGGGT

112 S W R K Y G Q K Q V K S S R S F R S Y Y

1321 ATAGCTGGAGGAAGTATGGCCAGAAGCAGGTGAAGAGTTCTAGAAGTTTTAGAAGCTACT

132 R C S H S N C H A K K K V Q R C D Q S G

1381 ATAGGTGCTCACATTCTAATTGCCATGCGAAGAAGAAGGTTCAACGCTGTGATCAATCTG

152 Q V I D T V Y I G Q H N H D L S Q N K C

1441 GTCAGGTTATCGATACTGTTTATATAGGTCAACATAATCATGATCTCTCGCAAAATAAAT

172 N I S R G S A S S A K L T A S S H I V D

1501 GCAACATCTCAAGAGGGTCTGCATCATCTGCTAAGCTTACTGCAAGCAGTCATATTGTTG

192 S D N K V D N A D V S I C W E D G R Q S

1561 ATTCAGATAATAAGGTAGATAATGCAGATGTGTCTATCTGTTGGGAGGATGGAAGGCAAT

212 S L H M T E S E Q Q S S S S S N G N F G

1621 CATCACTACACATGACTGAATCAGAACAACAAAGTTCAAGTAGTTCTAATGGCAACTTTG

232 I K G E E Q N G T E L E S S K F

1681 GGATTAAGGGTGAGGAGCAGAATGGTACTGAGCTAGAGTCAAGCAAATTgtgagtcgttt

1741 gcaggatcctcttgtatcagttttaagttaagcttctcttcaacatactcattgttttgg

248 Y C T S E I G H E T K N S C G I A K

1801 tttacagTTATTGTACAAGTGAGATAGGACATGAAACAAAGAACTCTTGTGGAATTGCAA

266 A E V P E K H G A E P W P R K R

1861 AGGCTGAGGTTCCGGAGAAGCATGGTGCTGAACCATGGCCCAGAAAAAGgtatgctcttc

1921 tgtagaatttggttctcttgctaatgaaaagctgcttccttttggtattttaaatgcaat

282 K K E S S V Y L A P V L K A

1981 aattggtctacaaacagGAAGAAAGAAAGCAGTGTATATTTAGCTCCTGTCTTAAAAGCT

296 T K D T N I V V H A A D G A M S S D G F

2041 ACTAAGGATACCAACATTGTCGTGCATGCTGCTGATGGGGCCATGTCGAGCGATGGCTTT

316 R W R K Y G Q K M V K A N S Y L R

2101 CGATGGCGGAAGTATGGTCAGAAAATGGTGAAGGCGAATTCTTATTTGAGgtatagttgt

2161 atattaatctatgcatttattggtggataagttataacacatttcttctgctgtgggtat

2221 tagattagccaaggatgttgacactcgtatgctttagacatggtattagacagcatatac

2281 aatgttctatggcatgcgttgtcacccatttctgtgcaggccgtaatattttgagtttat

2341 tgctgtttttcatgctcaatttagttcaggctaaatattttatctgaacttatcgttctt

333 S Y Y R C T S A G C

2401 actttcccctgtcatcttgatcaaatattagGAGTTACTATAGATGCACATCAGCAGGAT

343 P S R K H V E M A I D D A R T T T I K Y

2461 GCCCTTCCCGTAAGCATGTTGAGATGGCCATAGATGATGCAAGAACCACGACAATTAAAT

363 E G K H D H D M P V P R K Q K G S K S L

2521 ATGAAGGGAAACATGACCATGACATGCCAGTACCTAGGAAACAAAAAGGTTCAAAAAGTC

383 V H N S P P A N A N A A H C K K T S N L

2581 TTGTTCATAATTCCCCTCCTGCCAATGCGAATGCTGCTCACTGCAAGAAAACTAGCAATT

403 S S Q R F S S S T E W A V D R E E N M M

2641 TATCCAGTCAAAGGTTTTCAAGTTCAACTGAATGGGCAGTGGACAGGGAAGAAAATATGA

423 D E K V L E L G G E M A L E S A Q T L L

2701 TGGACGAAAAGGTCTTGGAGCTTGGAGGTGAGATGGCATTGGAGTCGGCTCAAACTCTTC

443 S I G T E L R P C *

2761 TCAGCATTGGAACTGAACTTAGGCCATGT**TGA**gaaacgaattgtaatttaaactgaggca

2821 gaaacttatctgaatgagctccatcttcgatggagattttgcaccctccccaactgaagt

2881 ttcaacgaagttgacagtttttccatggtgatttccttacaaggtttctgtacagcatct

2941 tagtatctatagatcattgatttgtagcttcctcaattcttgtaatagaggttttctaat

3001 cagcaattccattcagcttggaaggtcaagattgattcattgaatcatacaagagaattc

3061 tatttagcctattttaaaacttcaacgtcccagcttcatattatccaagaaaaaactgac

3121 tggcatctaatgacatgctaatgtcaatgacgcactgtcgtcagaagcagcaatttacga

3181 tgatccgcttgatatttttcatttaaagaggggcaggtcataaaatgcagtaacttggg

3241 gcttttcgttttccacaaaatggcgccatcataacttccatggagacattgaaatttcat

3301 cagttgccatgtcattcgagcgtgatgcattaacctaaagttgcatccaacaagggcttt

3361 tcccttcgtttctaggtgacaggaaaaccaacatttacatttactgctggcctgctacca
3421 catctaagtctagcaaaatcttatacaagcacatcctcgtgtcaaaaatatgccaatgaa
3481 tgagaaaaacagcattttagctattacagcatattgcgttgggaagtgattacagaaaat
3541 ttgaatggctcaattgccacctagccatagaatgtgagctagcagcactctccctatttt
3601 agactcttaattgatccttctcacatataagattctcgtattgctgtgggcagctctagc
3661 tcagattcaagctcgcttagcggagagaaatggcagtccaccatgtcttcacttacttct
3721 cttaaactaggaggatc
