# Supplementary material for: Gene Structures, Evolution and Transcriptional Profiling of the WRKY Gene Family in Castor Bean (Ricinus communis L.)
Source: PLoS One. 2016 Feb 5;11(2):e0148243. doi: 10.1371/journal.pone.0148243 (PMC4743969; doi:10.1371/journal.pone.0148243)
Supplement: S7 File — (PDF) [file pone.0148243.s007.pdf]

S7 File. The gene model for *RcWRKY18*. The coding region is marked with uppercase letters, above which is its deduced amino acids. The transcribed untranslated regions, including 5' UTR, intron and 3' UTR sequences, are marked with lowercase letters. The start and stop codons are blacked and the misannotated sequences are boxed.

1 cagaagtaggtgaaatatagttgcctttaacccttgggtgcaagataggtattcagactt

61 tttttcaaaatccatatctatcaaaaaccgcctcctcttttcttctttctccatgtatg

121 tagacatgttctcttctgtccaacctcttctcaccttccaccacttcttttgaccctata

181 tatatatatatatatatatatatacctaatccaaaacatcttcagttcctctctgaacat

1 M E G Q E T P Q P P L L V H

241 tctatctggagccccacaa**ATG**GAAGGACAAGAAACCCCACAACCACCATTATTAGTCCA

15 E L P P Q N S P Y L F T P P H P L L P S

301 TGAACTGCCACCCCAAAACTCTCCCTACCTCTTTACACCACCACATCCACTACTTCCATC

35 S S L H P P V I D P Q V V L P D I D W V

361 ATCTTCATTGCACCCTCCTGTAATTGATCCTCAAGTTGTTCTTCCTGATATCGACTGGGT

55 A L L S S Q S V V G E N R P M M M E N A

421 TGCCCTTCTCTCCAGCCAATCAGTAGTTGGTGAGAATAGGCCAATGATGATGGAAAACGC

75 S L I G E T G A E E E K G N K D K L R K

481 TTCTTTAATTGGTGAAACAGGAGCTGAGGAAGAAAAGGGCAATAAGGATAAGCTAAGAAA

95 S G R I K K H I T P R F A F Q T R S A D

541 AAGTGGCAGAATCAAAAAGCACATCACGCCAAGGTTTGCTTTCCAAACCAGGAGTGCTGA

115 D I L D D G Y R W R K Y G Q K A V K N S

601 TGACATTCTTGATGATGGATATCGATGGAGAAAATATGGGCAGAAAGCTGTCAAGAACAG

135 S Y P R

661 CTCATATCCCAGgtgtgcacatgtaaatagttctcaaggtcatggacttaattaatacaa

721 tgctcaaaaactcaattcataatgccgcttgagatagcaataaagattgtggatcgtctt

781 tattacatttttcagcaggttttataaataccatgcatgctttaattatataaaaactga

841 gttagttaaggtttattaattttaggtgagcgacaatgaaaactccaagtttctcctaga

901 atagagacttaagaattctcatctcaactagacataatactttatatagttatagctagt

961 tatgttaatgtttgataagaatgtctttcatttaagaattgaactgaaaactctgaacaa

1021 ttaagcaataatggagacaattaattaactaaaatagttatttgtgatttcaaaagtaaa

1081 taaaatagtagtgttgtaatgtgccgttttgatttgaaatttggtcggatatgaagctct

1141 aattgatggaaaaatatatagttatgatacagaatagacctaatgatgctatgttaaata

1201 atatgtttatgctaaacttaaatgaaaattctggttaggaaatattttttttagtcttaa

1261 ttatataaaaaataataatctgaaattggcaaaaagaacctaaggaaaaatctcgcatta

1321 atcagaaaaaataattaaatgtagtcactaattacataaagagtgcttggcttctaaaaa

1381 cttatataaatacatctcataaaatatgttatttaattaattactataataagtcactgt

1441 atttatcattaatatagcaaacttgctgtcaaaatagtgccggacgccataatattattt

1501 agcggcaatgtattttggtcgcttatggtctgttcttttttttttctttgtcgttgttga

1561 atatattatcaacctgccaccacttttgcgagctcgctcgcagaagtgattattttcgac

1621 ttcccctcgcgcgcaaataaaaaaaaataaaaaaaaaattgcaggcgagagagggttgag

1681 acaaataacgacggtaatggcagtcgttaacgcgacgccattagcgataaatatttagtc

139 S

```
1741 gtataaagttttctagtgtctgatataaatattcttctccggatttgaaaaataagGAGT
 140 Y  Y  R  C  T  H  H  T  C  I  V  K  K  Q  V  Q  R  L  S  K
1801 TACTATAGGTGCACCCATCACACATGCATCGTGAAGAAGCAAGTGCAAAGGCTATCGAAG
 160 D  T  S  I  V  V  T  T  Y  E  G  I  H  N  H  P  C  E  K  L
1861 GACACGAGTATTGTGGTGACCACGTATGAGGGAATCCATAACCATCCTTGCGAGAAGCTG
 180 M  E  T  L  T  P  L  L  K  Q  M  Q  F  L  T  R  F  *
1921 ATGGAAACCCTAACTCCTCTTCTCAAGCAGATGCAGTTCCTCACTAGGTTCTAAtttgtt
1981 taagtttaatacctagctacttacatgattatgatattggaatgtttcatcaacgacaga
2041 acccgaaaagctcagtcattttatgttgttatatacgtttctttttgtaaattgaaatgt
2101 caaattcgctgttaataccgcaagatcaataaattttatgctttttatatgtacttctcg
2161 gaaaatctgcaatcttttgtttcaccttagattctttgacgtgtattaattaatcgggaa
2221 tagaaatac
```
