# Supplementary material for: Gene Structures, Evolution and Transcriptional Profiling of the WRKY Gene Family in Castor Bean (Ricinus communis L.)
Source: PLoS One. 2016 Feb 5;11(2):e0148243. doi: 10.1371/journal.pone.0148243 (PMC4743969; doi:10.1371/journal.pone.0148243)
Supplement: S8 File — (PDF) [file pone.0148243.s008.pdf]

S8 File. The gene model for *RcWRKY20*. The coding region is marked with uppercase letters, above which is its deduced amino acids. The transcribed untranslated regions, including 5' UTR, intron and 3' UTR sequences, are marked with lowercase letters. The start and stop codons are blacked and the sequences of the first intron and the first two exons that were not annotated previously are boxed.

1 agccacctctcagtccatgttaaaccaaggattatttgaggagcaagagattccttctac

1 M G F F S L P Q N M S F P Q L G S C H

61 acag**ATG**GGTTTCTTTTCTTTACCTCAAAACATGTCCTTTCCTCAATTGGGTAGTTGCCA

20 Q S L K G I F T I P H S L A V D A P S S

121 TCAATCTCTTAAAGGCATATTCACTATACCTCATTCACTTGCAGTTGATGCACCCTCTTC

40 T A N L T E I L L S S S S S T A T K Q R

181 TACTGCTAATCTCACCGAAATCCTACTCTCTTCATCATCATCCACTGCTACGAAGCAAAG

60 D D F T S N F G G P H L L S L Q R S S A

241 AGACGACTTTACTTCTAATTTTGGAGGACCCCATCTTCTTTCTCTGCAAAGATCTAGTGC

80 N L W

301 AAATCTCTGgtaaagataatacactttttacaaacacaatcatgcaaaaaataccctcct

361 ttttcagttttcatggttttatcactttttttttctttattctttctctctattttttatc

421 acttagtttaaatttaaactagctagattatattctaacttcgtttccccaatcaatgaa

481 atataagtcatactagatctcttaattttaatggaattaataagatattcaatttatgaa

541 tcttcataattttcatgcatgtaccctctccctttcactatacatataatgaagtgaata

83 A W G D V N E C L S N K R

601 tttattggaattgttttcagGGCATGGGGAGACGTAAATGAATGTTTAAGCAACAAAAGA

96 S S V E D D H H H H H H L G I S T M K M

661 AGCAGCGTAGAAGACGATCATCACCATCACCATCATCTAGGGATTTCTACAATGAAAATG

116 K K I K A R R K V R E P R F C F K T M S

721 AAAAAGATAAAAGCAAGGAGAAAGGTAAGGGAGCCTAGGTTTTGCTTCAAGACAATGAGC

136 D V D V L D D G Y K W R K Y G Q K V V K

781 GATGTAGATGTGCTTGATGATGGTTACAAATGGAGGAAGTACGGCCAGAAAGTGGTAAAG

156 N T L H P R

841 AACACACTTCATCCCAGgttagacattcaaatctaataaaatttcaaatcctatatatgt

901 atttgttttctagcttttcttgatcttcttgttcttgttgttgtcgtttttgtgtacccc

961 atatacacatgctatgctcctcttccctttctctctctctctcacatttttttttttcttt

1021 ttctttctgtcgttctcattattatcgttgttatgattattgttataggcttttacagac

1081 ttggtgcaggagattttgatggataaatgtgtgtatatatatgtgtgtgtgtgaatatta

1141 atcattatttgataataaaaataaattgaagaaagtggccaaaaagaagtaattattatg

1201 gctttcaaaagacaaaatcgcaattgtgaagaaagtaaagaaaaggaaaataaaaaaagt

1261 gctaacaattgatcaatcataatgtcatgtgtaaagcaaagtatgacataatattcttga

1321 atcaaaaatgaaagttagacaaacagcataatattcttgatacactttgattaaacttgg

1381 aatcatataagaatgattccatagcgaagatatgatgagattatgtctataatattgaat

1441 gccgagtttagattacttcctcatgatagttgaggttcttgtgtatgtaaaatgtgcata

1501 actagattcttgtatttaacttgaaaaatgggtataaatgttttaccaaaaaaatatgtt

1561 gaattacaatacactttcatctctccgagaacaaggaaaagagataccatttgcttgtct

1621 gcgtttatgacccaagagaaattcattgatagacaaagtctgctctaagtaatgtgacat

1681 aataatttgacatgactcatttttattagatgtgtctatgaagaatgccaattggagaat
1741 ttctttccaatcccagaatgttataatcttaggatttataacaagagaggtggactttta
1801 gatttagcagttctttgacaatcttgtaatatagagataaaagattatttaagatatact
1861 ttagagttttccatctgaacttttgacgtgtacaccgcaacaaataagagagtgacatgt
1921 gtatatgagtattttagactttgacatttgatgattgatatatactcaatttatgctaag
1981 catatatcagtttagtgtatagacgagacacacctaaactagacaagaaatttcccaact
2041 attatttgtcttggccgggaattgcattgttcatttgtcagtacaacatagaacgtgatg
2101 aggcttattgctcataatgtccattaaaattctacagcaatgatttcagctttttgaatt
2161 tgtaaaatcttgcatgattataaatgagtacgttgctcatttgttttgtgctttgcatgt
2221 atgtctttcttgtgagcatggccacatatatatagccaaagtcaaaaaagtggggatcct
2281 agttttcaaaatacaattttcctccaactagtggctacccttaccaacttggcttagagt
2341 tttaactatagttttgagcaatacatgcatctaaggccacatgcatctagaaactcttgt
2401 actattatagtcaaatttgcaattaatagaagacttaattaatactgaatactattagtt
2461 aaaaaagacaaataaaggacaaaatactaaatggattagtgggtaatgctgatgtggcct
2521 tctaaacaaaagacagtaataataaggataaaattcttacactgtaatagttaaacaaac
2581 ttctaatattgacaaggttagaaagaaaggaaaaaaagaaaagaaaaaagagaagaagat
2641 tacatagaggctgctccttgcacttttcactttttcctgtttctttttcttcctccattt
2701 gtgtttgagcagctatatgctattattgaggaaattaacttttcagttatcacgctccaa
2761 aggtgttgtacgtaaagtttctttgatcattatgatttttacagtgcatggacttggtgt
2821 atacctgcagtaaaatatgttcagtgtgtatatatatatatatgtacctttctttattca
2881 ggggtaaatatatatatatatatatatatatatatatatataacaattctccaaaacatg
2941 aactaaaatgtgtttgattagtgagaactttttggcaataataattcaaagtttttgaac
3001 agtagcctcccatcatttcttcatttgcttaagaacgaacttagtttcatattgaattaa
3061 gtaacaaaagcacaacactagatgcttgagtcagagcaacttaaaaaaaaaaattatttt
3121 tacccaattggtgaatttcacatacttttggtagagatagatcatgcgggaaaatctgat
3181 gtcaactaatatagacaaaatcaatgataaaagtattaaaaatagtgttgaatcttgaat
3241 catttttttccttctaaaagtagatatggttagcatgttagaagtatagtagtgcgtta
3301 gaaatgagacggggaattctttttttataaagtgaggaataagccattataattataatt
3361 ataattaaaaaggagagtcttcgattcgagacctcaacttccgatgttaagcgtctggtt
3421 tttaattcatataactttatacttggcctgtttcttaaacttttatatatataactatatag
3481 attttttctacacattccatcctgtgatacttggcctctcctaaaaactaacttgcctc
3541 tgcccctgaacacaaggatcatttttttatatatattcttgtgctacttctttatgttca
3601 tatcgtgtgatttaagttacagtttccatagagaattccatgcagttaattaacttcttc
162 S Y Y R C T Q D N C R V K K R
3661 tggtacaaaatccagGAGCTATTATCGCTGTACACAAGATAACTGCAGGGTAAAGAAACG
177 V E R L A E D P R M V I T T Y E G R H A
3721 AGTGGAGAGATTGGCAGAAGATCCAAGAATGGTGATAACAACGTATGAAGGGAGACATGC
197 H S P S H D L E D S Q A Q S Q L N N F F
3781 ACATTCTCCATCACATGATCTCGAAGACTCCCAAGCTCAATCCCAGCTTAACAATTTCTT
217 F *
3841 TTTC**TAG**ttctctttgtgtttttttcttttctttctcttttaattaaagattacttgatt
3901 cccaatatgtttggagctttgttagtaaatttgctgcttgtgtaagagcttttccattat
3961 tatgtctttgattaatatactacttactgtgttcaaggcatgtaaaaaataaaacagtat
4021 atccatgaattcatatatggccgcatttctaaggcttccatttcca
