# Supplementary material for: Gene Structures, Evolution and Transcriptional Profiling of the WRKY Gene Family in Castor Bean (Ricinus communis L.)
Source: PLoS One. 2016 Feb 5;11(2):e0148243. doi: 10.1371/journal.pone.0148243 (PMC4743969; doi:10.1371/journal.pone.0148243)
Supplement: S9 File — (PDF) [file pone.0148243.s009.pdf]

S9 File. The gene model for *RcWRKY21*. The coding region is marked with uppercase letters, above which is its deduced amino acids. The transcribed untranslated regions, including 5' UTR, intron and 3' UTR sequences, are marked with lowercase letters. The start and stop codons are blacked and the fourth exon misannotated is boxed.

```
   1 agagagacagagagagtgactgtggaattctttcattttctgtagaaattcgaaagtaga
  61 tgagagaatatttcccggagacttgctattcgacgtttacttgttcaccagacttcatcc
 121 tcgtgcaagacaggcgcagttctttctactgacgcatatatgcaccattccataaacagg
 181 agaaggagaacaaaaacaagaattgggagtggaagcttgatttgataagcgttactaaaa
   1      M  E  G  G  E  R  E  V  P  N  Y  E  L  Q  V  S  F  S  T
 241 gcatATGGAAGGAGGAGAAAGAGAAGTTCCAAATTATGAATTACAAGTCTCGTTCTCGAC
  20  P  Q  A  I  H  E  M  G  F  V  Q  F  E  E  N  Q  V  L  S  F
 301 GCCACAAGCAATCCATGAGATGGGTTTCGTACAATTTGAGGAGAACCAGGTTCTAAGCTT
  40  L  A  P  S  H  S  Q  Q  S  S  Q  I  S  Q  P  L  N  T  T  T
 361 TTTGGCACCTTCACACTCACAGCAATCTTCTCAGATATCTCAACCACTCAATACCACAAC
  60  T  T  T  Q  I  G  F  S  T  H  N  D  Q
 421 CACCACTACCCAAATAGGGTTTAGTACTCATAACGACCAGgtcagttagattcttgaggt
 481 ttgttatatatatacatactaagtttatcctgagattctgtttacttttcctggtactta
  73                                                            V
 541 tttattgattcactagtcaacctttgaaatttattcattcttgttatggaaatcttagG
  74   G  N  L  D  P  K  A  T  N  E  D  T  C  T  A  S  A  N  D  G
 601 TAGGAAACTTGGATCCAAAGGCTACAAATGAAGATACCTGCACTGCTAGTGCTAACGATG
  94   N  N  S  W
 661 GCAACAATTCTTGgtattcttcttctctttgtttcttctcctttatcttttcaagggtgg
 721 ggatctttcttccttgagcatgcacacatacaacaatatccaagaaaaatattcttcaag
 781 gtataagttcaatattggtttttaaaaaaaggtataagttcaacagaatctaaggagatc
 841 tatccacccgcatgtggaagagaagagagtgctcctgttcagccacagtactacttttct
 901 tttgtccagcaatatatcttctctctttctctttctctcccgtggctgccaggaggaaag
 961 tgcgaacttgacatcataaaagtctcagatccaccattccattaaaccgatctattttta
1021 tttatcaatttcctgttcactttcattctttccccctatcttattgcctggaaacaaaaa
  98                            W  R  S  S  S  S  S  E  K  N  K  V
1081 gcctgaagatttgaaatcttggtagGTGGAGAAGCTCATCATCCTCAGAGAAGAATAAAG
 110   K  V  R  R  R  L  R  E  P  R  F  C  F  Q  T  R  S  D  V  D
1141 TGAAAGTCAGGAGAAGGCTTAGAGAACCAAGATTCTGTTTTCAAACACGAAGTGATGTGG
 130   V  L  D  D  G  Y  K  W  R  K  Y  G  Q  K  V  V  K  N  S  L
1201 ATGTGCTTGATGACGGTTATAAATGGAGAAAATATGGCCAGAAAGTAGTTAAAAATAGCC
 150   H  P  R
1261 TTCATCCAAGgtaaatctttccgttccttctcgattccttgatgcatatgcaccaaaaat
1321 tagcttaattttagttatttgaagttaatgagcaactatttattctgcttttctattcat
1381 tatatctgatttcatgcattaatgctgtcatcatctaaccctaatcaatgatcttcttgt
1441 ttttactgtaaaaagctgcttttcacataaaaatacgagcttaaatgcaggttaattata
1501 gctctttaaagataataatattgaaaataatatataacaacatctactaaccctttgaag
1561 caatacacaataatttcgttcaaagttatcaaccagtagaggagaatattaggagtacag
```

1621 tgtgtcagccattcaagcttcttttaatcattggtgggagagtcaggagcaatacttagc

1681 cttcgcaagtatcaacaacatctcgttctttttcttaaaaaaaaagaagattaattcttg

1741 catttatatacctatgcaagtgtctattgcaaagtaaatgctgagttgagcgaaatcccc

1801 tctccagagcatgaagtatctttcctatttgaaaattctgagaattcaaatgcctttgcc

1861 taagttcttgccgtagtgaaagaaaaagaagcattggaaaagaaaattgcaattggagtt

1921 tattaataatattattcaaaagagacattaatttgaagggaaaactcatgcctagccttg

1981 atgtatccatacactaaaaattgatagataattgatacatattcattaattttaaatata

2041 acatgtgtcaatcctccaattttaaaatataaaatacaaatatatacgtattattctctt

2101 actaatattgatatatacaccaagtctagataaaaatttctcacaactaataagagagtg

2161 atacatatggatgagtattttataatttgatatgagatgactgacacatactatattaat

2221 taaaactaatgaatatatatcaattatctatcagtttagtgtatagatgaagacatacat

2281 taaaaactgttttattaggtttttgtacctgtcattgcactacaataccgcaagtggtat

2341 ctcagaattagagaagactgaaacaggagtctaaaagctgaagagaaaatgaaaaatcta

2401 ataatagttttatgtagtacccaagatgagatccgaattataatgcataagaaaaccgaa

2461 tgtataagaagtctatgtgaaatctcaaccaaaatggtaggtacaagacacactgacatt

2521 atagaagacatttatctgttttaagataaatagattctttaaatagggatttgaaataat

2581 tccacatgaaactaatcattaacttggattttgatactaatcacgttacaatattttgac

2641 agttgcttaaccatctaccgtcattgaaattgtagaatctgtttcactaatagcattaca

2701 tacaagaagaaatttacgttgctctcattttcctgcgtcttacttctcttatctcattca

2761 gtttagttgcacccttatatacacacttgcaattcgcttgagaagttctaaacgaagagt

2821 tctgtttataggcatctctacaggtagacgnnntttttaatatcttatatattacataag

2881 cattatgagatccgttgtcatggaatggataaactccttaaaaaattagaaaggttaaaa

153 S Y Y R C T H S N C

2941 ggttttcttatattctgaatgactagcagAAGTTACTACCGATGCACCCACAGCAATTGT

163 R V K K R V E R L S E D C R M V I T T Y

3001 CGAGTAAAGAAAAGAGTCGAACGATTGTCAGAAGATTGTCGAATGGTGATAACAACATAT

183 E G R H N H S P C D D S N S S E H E C F

3061 GAAGGTAGACACAATCACTCTCCATGCGATGACTCAAATTCATCTGAACACGAATGCTTC

203 T S F *

3121 ACTTCTTTC**TGA**tagttgaatttttattaaattataaaagaaacattgttgtaattatta

3181 taatgtgttagccagcttgtg
