# Supplementary material for: Gene Structures, Evolution and Transcriptional Profiling of the WRKY Gene Family in Castor Bean (Ricinus communis L.)
Source: PLoS One. 2016 Feb 5;11(2):e0148243. doi: 10.1371/journal.pone.0148243 (PMC4743969; doi:10.1371/journal.pone.0148243)
Supplement: S10 File — (PDF) [file pone.0148243.s010.pdf]

S10 File. The gene model for *RcWRKY22*. The coding region is marked with uppercase letters, above which is its deduced amino acids. The transcribed untranslated regions, including 5' UTR, intron and 3' UTR sequences, are marked with lowercase letters. The start and stop codons are blacked and the fourth exon misannotated is boxed.

```
   1                                                 M  E  K  V
   1 atgtgctgttgatttttattatatgtacttgacaatttaaagggttctATGGAGAAAGTT
   5 R  E  E  K  K  M  I  K  I  G  N  S  S  A  A  V  A  A  N  S
  61 AGAGAAGAGAAAAAAATGATAAAGATTGGGAATTCATCGGCTGCAGTTGCTGCAAATTCA
  25 T  T  F  I  S  E  L  Q  E  E  M  P  S  I  N  V  S  L  P  L
 121 ACGACATTCATTAGTGAGCTACAAGAAGAGATGCCTAGTATTAATGTTTCTTTACCATTA
  45 S  S  A  A  S  S  I  T  T  T  S  I  F  D  M  M  M  P  C  D
 181 TCATCCGCTGCATCATCAATAACAACAACAAGCATCTTTGACATGATGATGCCATGTGAT
  65 Y  M  G  E  K  G  Y  S  L  G  F  L  D  L  L  D  G  N  N  Q
 241 TATATGGGTGAAAAAGGTTATTCTTTAGGTTTCTTGGACTTGCTTGATGGGAATAACCAA
  85 D  F  G  A  T  S  S  L  F  D  W  F  Q  F  Q  T  P  I  L  P
 301 GATTTTGGTGCTACTTCTTCTTTGTTTGATTGGTTTCAGTTTCAGACTCCAATTTTACCC
 105 D  H  H  Q  Q  Q  Q  Q  P  L  P  S  P  A  S  T  V  P  E  T
 361 GATCATCATCAGCAGCAGCAACAACCACTACCATCACCGGCATCTACAGTGCCTGAAACG
 125 S  E  V  L  N  T  P  A  T  P  N  S  S  S  I  S  S  S  S  N
 421 TCGGAGGTGTTAAATACTCCGGCGACTCCTAACTCTTCATCGATCTCTTCATCATCTAAC
 145 E  A  G  I  D  S  S  S  Q  V  K  A  G  D  E  D  E  Q  D  Q
 481 GAAGCTGGAATTGATTCTTCTTCACAAGTTAAGGCTGGAGATGAAGATGAACAGGATCAA
 165 D  K  N  K  K  Q
 541 GACAAGAATAAGAAACAgtgagtttttattttattttattaagcgtgttatgggtgttt
 601 tctttttttctttttcttgatagatttgttgctttctttgttgtgtttgttattgttta
 661 cttagttaagtgagctagttttggatgaaacatgaagttgaaaagtgataagtccagtga
 721 tgggttctgtttctcttttatgttttctgcaattttatggggtttctttagaaattactg
 171                           L  K  P  K  K  K  N  Q  K  R  Q  R
 781 aaagtgtaatgttttatgggtttagGTTGAAACCGAAAAAGAAGAACCAAAAAAGGCAAA
 183   E  P  R  F  A  F  M  T  K  S  E  V  D  H  L  D  D  G  Y  R
 841 GAGAACCAAGATTTGCTTTCATGACAAAGAGTGAAGTTGATCATCTAGATGATGGGTATA
 203   W  R  K  Y  G  Q  K  A  V  K  N  S  P  Y  P  R
 901 GATGGAGAAAGTACGGCCAAAAAGCAGTGAAAAACAGCCCTTATCCAAGgtatcatgaaa
 961 atattatctttcatcttgtctcctatatatatatatatatatgttaatgttaatggctct
1021 gatacggttttatgtatattaatcaaattttacttgtattgcaagaatatgcaacccttt
 219
1081 tttaggtttggtcttgtgctaaaaaaagattgttaatttatttttatttttttcgcagG
 219 S  Y  Y  R  C  T  S  A  G  C  G  V  K  K  R  V  E  R  S  S
1141 AGCTATTATCGTTGCACTAGTGCAGGATGTGGAGTGAAGAAGAGAGTAGAGAGATCATCT
 239 E  D  N  T  I  V  V  T  T  Y  E  G  Q  H  T  H  P  S  P  V
1201 GAAGATAACACTATTGTTGTTACGACTTATGAAGGGCAACACACGCATCCAAGCCCAGTA
 259 T  P  R  G  S  I  G  F  L  P  D  S  S  A  F  G  A  A  N  S
```

1261 ACACCAAGAGGAAGCATCGGATTTTTGCCAGATTCAAGTGCTTTTGGTGCTGCAAATTCT
279 S F V I P Q P R P Y Q Q Q Q Q Q Q H A Y
1321 TCTTTTGTTATTCCACAACCTCGTCCATACCAGCAGCAGCAACAACAACAACATGCCTAT
299 M Y S S S P S L N I T T S T S S S I N P
1381 ATGTATAGTTCATCACCTTCTCTCAACATTACCACCAGTACAAGTAGTTCTATTAATCCT
319 P F S P S F L H H E R R F S P S P A S L
1441 CCATTCTCTCCAAGTTTTCTTCATCATGAGAGACGGTTTAGTCCTTCACCAGCTTCTTTG
339 V R D H G L L Q D I V P S Q M R K D A T
1501 GTTAGAGATCATGGTCTTCTTCAGGACATAGTGCCATCCCAGATGAGAAAGGATGCTACA
359 E E *
1561 GAGGAG**TAA**atgaaggacgttattaaggtttcgtgttagttactggtatgtaattctcta
1621 actgacttatatggatcatcatcatcagcagcacccatttcatgacacagaaccttattg
1681 ttggaaatgatgatgatggctatagccttttagcttatgcaggcctagctagttttcttt
1741 ttctttttcttctttggttcttttctgttattaattctttgtatccagagattatctttt
1801 tatgaagataattgagttttgtatcatccttttcccccactctagtagttattattccat
1861 ttcttta
