# Supplementary material for: Gene Structures, Evolution and Transcriptional Profiling of the WRKY Gene Family in Castor Bean (Ricinus communis L.)
Source: PLoS One. 2016 Feb 5;11(2):e0148243. doi: 10.1371/journal.pone.0148243 (PMC4743969; doi:10.1371/journal.pone.0148243)
Supplement: S11 File — (PDF) [file pone.0148243.s011.pdf]

S11 File. The gene model for *RcWRKY25*. The coding region is marked with uppercase letters, above which is its deduced amino acids. The transcribed untranslated regions, including 5' UTR, intron and 3' UTR sequences, are marked with lowercase letters. The start and stop codons are blacked and the misannotated sequence of the first intron is boxed.

1 tttttttcaaaaaatgtctttgatttgtatattttatgattctgtttgaattatagcaga
61 cccaatcctatcaattaattgctggtaaacaatttttgggaaataaaattaaattaatag
121 agtttgtttccttaagtttgaaggcaaaacgtgttgagaacattccttgttataataacc
181 tgccatagtagaaagcagcagcaatctcaaagagaactccaaaatttactcaaagcattc
241 cattcttgataaacagtttctttataatccggttagcagtcagcattggagaaatgaacc
301 caccactataccttacaacacatgcacttgcaaagttacaaatatatatagtaaatatta
361 acagatgttgattattgataagactcttttcaaaattaaattggtgaagcacatttatat
421 ttgctatttgaatcggttgctcagagtgcaattcataagtgaaatgttaaaaacaataag
481 gaaaatcaaaatcaaacaaatagacattgctaagcatcaatatgattttctaatgctgtc
541 tctcttgtcaggaaagtatccactatttctattcatgcgcgtgggggcggcccttttcct
601 tcccttcttcatcttcattcctgtccctcgcttaaatatcaccctctgtactatttttgc
661 agctgacgccttttacttttctaacttatctctatttcttttttctttttttttttctgc
721 ttaaaaaaaacggaaataataaatttaatgtactgactgacagacacacctttcagaact
781 cttcaaaagaaaaaaagaaaattttgaaaaactacgcgtccttcccaacacaacaacaac
841 gaacttctttcttcgcacctcaaaccctaactcctctctctctctctctctctctctct
901 atattcattctgtttaacttttttatctttctaaaactccctctcagttctcacgtcattt
1 M D D K D N T G D D
961 caaatccccaatcagtagatctctgggttcc**ATG**GATGATAAAGATAATACCGGCGATGA
11 F V T D S T W T L G P D S D G V S Y F F
1021 CTTTGTTACTGACTCCACCTGGACGCTAGGACCTGACTCCGATGGCGTCAGTTACTTCTT
31 A T E R E S S I L S E F G W N L H T S E
1081 TGCAACCGAGCGAGAGAGCAGTATACTCAGCGAATTCGGATGGAATCTTCACACCAGTGA
51 P E R L D E L D P I Q S Q N T A D L A G
1141 ACCGGAACGGCTCGATGAACTTGACCCGATACAATCTCAAAATACAGCCGATTTGGCGGG
71 N L R F A P E S S S S S S V L Q G S V P
1201 AAATCTTAGGTTTGCACCTGAGAGCAGCAGCAGCAGCAGCGTGTTACAAGGATCGGTACC
91 T G R D T R T G T A I P G S G G G D A S
1261 TACCGGTCGCGATACTCGTACTGGTACGGCAATCCCTGGCAGTGGCGGTGGCGATGCTTC
111 T S N P S I S S S S S E D P P D K S A S
1321 GACTTCGAATCCATCAATATCGTCAAGCTCCAGTGAGGATCCGCCAGATAAGTCCGCGAG
131 S V G K P P E I P
1381 TTCCGTCGGGAAACCACCTGAGATACCgtgagtagtggatatacagaaattgaaaaatta
1441 gctcgaatctcgaggttttgcacttaaaataatggggataaaatcaagaaacagttgcag
1501 aactcggtcctttctgtttggagagagggagagtgcaatttatgcgtccgtacactctac
1561 aaactgtcaaaaaaatttggttttagctcatgcaagttggggaggagaggtagtttcgta
1621 atttatcagaaaaatagggctaaatgggataatttttctcgaaacaaaaggggcattttg
1681 ctaagagaaaaaaagaagacagagccctcgaacccgccgttattgcggcgggaatggcgt
1741 gctcttaactttttttaacacgaacttttaaaattctcttttctttttctctttcggttt

1801 ctaattctattagtaacacaattataaagctgaatatatacagcagaaggaaaaaagaag
1861 ttccttgatacttgttttattagttttttggtcgatattctttcaagaaaaggaaata
1921 ttgtaggagatattctgttatgctatgtttgtttttttttttttgttttaaatttaaata
1981 aatcaattgatgcttgttttatgttctagattcatcccctttggatagtgctttatacaa
2041 ttataatcattttgaatgcaatacgtgtctgcaagttgcacgacacacacaaatgcttca
2101 ccgtattgaatctcattttctattttcggtttttcttattctgcatgaacatattcatgt
2161 gcattactatcactgcatatgtactattatttttcttgaaaaaactatattctacccgct
2221 tccagcattgcatataaatgtcattcaatccttaccctatattatttattttgccatgag
2281 agattagttttctttttgtcttttccctcattggctttacatcatatcatttatgatgat
2341 atttgcatctagtttctagatttgatttgatttgatttttatttttcttatgttcatg
2401 gtcaatgccactgttttgctcattgtattgcccaatgggctaggaaatagtcttgatgaa
2461 ggatgtccatctctgtgcactcagatgttgcttcgttgttccatctccatgacgttctct
2521 tttatatccagggaaaaataaaaaaagaaccaaccattagtcccattttggcattaacct
2581 tttttccaatttcaatatcttaaaatcttctttcagctagccatctggactgcactgatc
2641 ttattaggaaacagcgagagaaatgtagttaccttcggaaagataacatgaagtgcacaa
2701 tcaattttctagcaacatggacacattatttactgctacacttgggcttttttttttc
140 S K G K
2761 agaattttatttgatttttcataattgtaggtgaaatatttttgtagGAGTAAGGGCAAA
144 K K G Q K R I R Q P R F A F M T K S E V
2821 AAGAAGGGGCAGAAACGAATCCGACAACCACGTTTTGCGTTTATGACAAAGAGTGAAGTT
164 D H L E D G Y R W R K Y G Q K A V K N S
2881 GATCATCTTGAGGATGGATACCGTTGGCGTAAATATGGACAGAAAGCAGTTAAAAACAGT
184 P F P R
2941 CCATTTCCCAGgtctgcagttaatctactttattaatttctattgtcccaaaggtatct
3001 acatttcctttcttccattgtcttcatcatttctccagcaacgtctatctaaagtttcc
3061 aatcaatcaattctactataagtagttctactattaatgatattagatataagtcgaatt
3121 ctattgaattgactcctttttactcaatatggttctacgaattttcaatttactagaaa
3181 ataaatacaataataagtatatttgataaacatttaaaattcacttaacaaaggaaaagg
3241 gcagcaatatatatataaaacaaaaaattggttttgcttttcagaaaagtttttttattt
3301 ttttcgctttctaacattggtttttgttgtctaaccatgtttgcttacaaaaagctaaaa
3361 actttaaaaaatccgacaagaaaaacgaaagagcaagctaatcaaacgaagcgttaagaa
3421 ttcattaacttctataattctatatttttcagccagttagaagaacttttagctttcatt
3481 tcttttccacgttagtttggtgtgcattcatggataaattctcaaaatgatctcaaggag
3541 gatcgaaccctagttggaaaggtaaagaaatcccttgactaaatggattaacactcttag
3601 agttttggccatcactgtttaatgtttactacaaaattatatattttactggtatgctaa
3661 acaagttccattaactctatttggaagattgtatatgcaaaagaaaagaccatattctca
3721 tttttaaagttcctgattcaaaatccgttgcttttaatatttcttgaaagtgtgtttgat
188 S Y Y R C T N S K C T V K
3781 ttgacacataaacatttcagGAGCTATTATCGTTGCACAAATAGCAAATGCACTGTGAAG
201 K R V E R S S E D P T I V I T T Y E G Q
3841 AAGAGGGTTGAACGGTCTTCTGAAGATCCCACCATTGTGATTACAACATATGAAGGCCAG
221 H C H H T V G F P R G G I I S H E V T F
3901 CACTGTCACCATACTGTTGGTTTTCCTCGAGGCGGAATAATTAGTCATGAAGTCACATTT
241 V N H M T P P V S Q F Y Y T G I Q T P R

3961 GTTAACCACATGACTCCGCCAGTTTCACAATTTTATTATACTGGAATTCAGACACCTCGA
261 E N P P T I T Q S Q Q T L P A Q T G E P
4021 GAAAATCCTCCAACTATTACACAATCACAACAAACATTACCGGCTCAAACCGGTGAACCC
281 G T L R E P N R R G P T D E G L L G D I
4081 GGTACACTGAGAGAGCCAAACCGGCGGGGCCCAACAGATGAAGGACTTCTTGGGGACATC
301 V P P G M R N R *
4141 GTGCCTCCTGGAATGCGTAACAGA**TGA**gacagaattggtacattgcttgccttccctaat
4201 tttaattattaaacttgatcctttagtatctatgaatcttgcatgaagatagcatttcca
4261 atcacttcatgtgtaattgacagagagagttagtacattggactttcgactaaaggtaca
4321 ctaaaatttaaaatcattacgtatgtcactggaaaaatttcagtagatgttatttttctt
4381 ttttaatttttttgtagactcttaagaggcctgctcttgaaatctatgtaagaattagtta
4441 atttttggcttttaaattaaagattaaactaattctgtgctgaattttctcaataaatct
4501 ctatataagtttatcaatcatgttaaatgataacaaaagatgttcatgagctgtcaaaaa
4561 tgaaagaaaacgagtacgagccttagaattgttaactataagaataaaatcagattttag
4621 ccatttagcgctaaaacttaatttagctcatgggaagaataaaagctctaaatatagcaa
4681 gatgcgtaatagaacttagttccactcatctgtatatgtagataacctttgatgaggtca
4741 gaagaaaaggagagaatccgatgtatataccagtcaactactatagagtttcagcttcta
4801 cattagtggacaagctagcgtctgaggcatgaatggttcttgagtgaactagtctgatat
4861 tatctgttagaaccacaggtatcacaccgcatatgtgacttatnnnnnnnnnnnnnnnnn
4921 nnnnnnnnnnnnnnnnnnnnnnnnnnnnnnnnnnnnnnnnnnnnnnnnnnnnnnnnnnnn
4981 nnnnnnnnnnnnnnnnnnnnnnnnnnnnnnnnnnnnnnnnnnnnnnnnnnnnnnnnnnnn
5041 nnnnnnnnnnnnnnnnnnnnnnnnnnnnnnnnnnnnnnnnnnnnnnnnnnnnnnnnnnnn
5101 nnnnnnnnnnnnnnnnnnnnnnnnnnnnnnnnnnnnnnnnnnnnnnnnnnnnnnnnnnnn
5161 nnnnnnnnnnnnnnnnnnnnnnnnnnnnnnnnnnnnnnnnnnnnnnnnnnnnnnnnnnnn
5221 nnnnnnnnnnnnnnnnnnnnnnnnnnnnnnnnnnnnnnnnnnnnnnnnnnnnnnnnnnnn
5281 nnntgaaccaatgctctacaaccctaaacgaaccagattcaagttaaacaaaatttttta
5341 aatatatacattgtaagttatagcaatgattgttgaatcttttattgtagtcccaaaatt
5401 tcgatatgattgcaatcattgaacttcattatgttttaatttacacattttatacttgag
5461 agattgatacatcacaaaatagctgataacaaaaggtgaggaaaaaattataatatatta
5521 aaacgaagataactaactaaaatgcaattgaagtttaataactaaaagaaagtttatgaa
5581 taaactgaaactaaatgtaaaaatttagttttaataattggtacaactaccctatgttat
5641 acattctcttttagatattttgatcttctccttgacttgttgaaaacttctttaattatc
5701 tacatataccacgacaactgcacttcttttctctaggttgtagataaatgaaacataata
5761 ccacaagtcttaagttcatcgtagtaaaatatttaagcagggttttccttcaaaggtttt
5821 ccataactcatcctaaacttggcttggtcttgaagtattcaagtagtcatatcgcatata
5881 tatacaattgttttctcccaagccggggacatatcatgcttagaggcttccgcagagaat
5941 tgtgtcactgtttgctgtttttccttgggtatttgcacttttcttattagggagtgataa
6001 atacgagtatttgtttagcatgattgaaaactctatataaccacttatattagattgtta
6061 tgagacaggtggctataccatgcgtcttttgtagcctatacattagtttgtgtaagaatt
6121 gcagtacatggcttaatgtttttgtgttccatatctgggtcgaccgacatgatcccatat
6181 gtgtaaaggtggattccaagaaacggtgacccaaaaaaattaatttcttatgctgccttg
6241 tcaggagttaaaatctttttaaaggtgcttgcttgatagaatataaccctttgtctcacc
6301 cacctgttttgtagcaaggcacgggtcaagtgtgtggaacattccctttatattatttta
6361 tctataggcatattcatgaagctacgtgttctatattttattcctcactattaagagaag

6421 ataaacccatttatgttttatcattcttatttaacctagccttcttaacacggtttacat
6481 atgtatatatgtatgtatggtgcagagaattcattttactttattgtggagaacaaaagg
6541 agcaagccagaattttctagctcgaagtgagtcctcaagtcttgtatttatctcttggta
6601 atagagtgcatgactttatacgtatctcacattgcagcaggtatttttcaattgattggc
6661 tcttatatagaacctttttcatttcaaatttgcattttgaggatggcttgctgtatgtgt
6721 aaatagctttcacagctgaaatattgttaacctaattctgcactcttttagtttgagtcc
6781 gctctgaattcagagtgttttaagaggtctgtactatatgtaaagttgatgatataaatc
6841 cttctttatttctaaatcaaatcttataatttgattcattaatcgtcctttttttttctgt
6901 ttgctttttgggtagctgtagttaattcatttctgtttgggagttcttcttcatgggagg
6961 tacgtgacccg
