# Supplementary material for: Gene Structures, Evolution and Transcriptional Profiling of the WRKY Gene Family in Castor Bean (Ricinus communis L.)
Source: PLoS One. 2016 Feb 5;11(2):e0148243. doi: 10.1371/journal.pone.0148243 (PMC4743969; doi:10.1371/journal.pone.0148243)

S12 File. The gene model for *RcWRKY28*. A, The coding region is marked with uppercase letters, above which is its deduced amino acids. The transcribed untranslated regions, including 5’ UTR, intron and 3’ UTR sequences, are marked with lowercase letters. The sequence of scaffold43951 is boxed and its overlapping sequence with scaffold30131 is marked in yellow. The start and stop codons are blacked. **B**, EST mapping graphical representation of *RcWRKY28*.

(A)

1 ctctatatatccccacactcgccactttccaccttgtttcttcaaaacgagtagagcctt

61 gattctttttatttccttctctatatgcgtatatatatatatatacgtgttcttgcattt

121 tgggttcatcaggaggactagctagctacaagaatttttgaaggatttactatcttcatc

181 aagaaaagttttcaagagaaaagtataaagatatacatagacttgtttcttggtcgtttc

1 M D Y S S S W V

241 agttttgcttcaagattcttaagtacaagttttatt**ATG**GATTATTCTTCTTCTTGGGTG

9 D T S L D L N L N P R R V K D D V P I Q

301 GACACTTCTTTGGATCTTAATCTTAATCCTCGACGTGTGAAGGATGATGTTCCTATTCAA

29 E R L A M A N D V Q S N F M E L G R N I

361 GAAAGGTTAGCCATGGCTAACGACGTTCAGAGCAACTTTATGGAGTTGGGAAGGAATATT

49 S V K E E

421 TCTGTTAAAGAAGAGgttagttatgtgagtcgatttcttgtttaagtgtgtctcgataa

481 tgattgaatcaattgtttcttgaaactgagaggttttgannnnnnnnnnnnnnnnnnnnn

541 nnnnnnnnnnnnnnnnnnnnnnnnnnnnnnnnnnnnnnnnnnnnnnnnnnnnnnnnnnnn

601 nnnnnnnnnnnnnnnnnnnnnnnnnnnnnnnnnnnnnnnnnnnnnnnnnnnnnnnnnnnn

661 nnnnnnnnnnnnnnnnnnnnnnnnnnnnnnnnnnnnnnnnnnnnnnnnnnnnnnnnnnnn

721 nnnnnnnnnnnnnnnnnnnnnnnnnnnnnnnnnnnnnnnnnnnnnnnnnnnnnnnnnnn

781 nnnnnnnnnnnnnnnnnnnnnnnnnnnnnnnnnnnnnnnnnnnnnnnnnnnnnnnnnnnn

841 nnnnnnnnnnnnnnnnnnnnnnnnnnnnnnnnnnnnnnnnnnnnnnnnnnnnnnnnnnnn

901 nnnnnnnnnnnnnnnnnnnnnnnnnnnnnnnnnnnnnnnnnnnnnnnnnnnnnnnnnnnn

54 T G A L V E

961 ngttttgaagtttgattaatttgttgtgttttaattactttcagACTGGAGCTTTGGTGG

60 E L N R V S A E N K K L T E M L T V M C

1021 AGGAATTGAACAGGGTGAGTGCAGAAAACAAGAAACTGACAGAAATGCTTACTGTGATGT

80 E N Y N A L K S Q L M D Y V S K N R D K

1081 GCGAAAACTATAATGCTTTGAAAAGCCAGTTGATGGATTATGTGAGCAAGAATCGCGATA

100 E L S P S R K R K S E S S N N D N T I A

1141 AGGAGCTTAGCCCTTCAAGGAAAAGAAAGTCTGAAAGCAGTAATAATGATAATACCATTG

120 M N G N S E S S S T D E E S C K K P R E

1201 CAATGAATGGGAATTCAGAAAGCAGCTCAACTGATGAAGAATCTTGCAAGAAACCAAGAG

140 E I V K A K I S R T Y V K T E A P D T S

1261 AAGAAATTGTCAAAGCAAAGATTTCAAGAACTTATGTTAAGACAGAAGCACCCGACACAA

160 L

1321 GCCTTgtaagtcatcaatcaatttaactaattctgcataactttatgaggcttgtttaga

161 I

1381 tatttaatgttttattttatgggcttttacttagcaattgcataaaattgttgtttcagA

162 V K D G Y Q W R K Y G Q K V T R D N P S

1441 TTGTAAAGGATGGATATCAATGGAGGAAGTATGGCCAAAAGGTCACCAGAGACAACCCTT

182 P R A Y F K C S F A P S C P V K K K

1501 CTCCAAGAGCTTACTTCAAATGCTCTTTTGCTCCTAGCTGCCCTGTCAAAAAGAAGgtaa

1561 tgttcctgatatttatcagaatcattttaacattaatgttctttcttacataaaagcaat

1621 agtttaggtttcagtctttgatacattcataaaataattccgtgaaatgaaatcaaagtt

1681 tccatgtgcattggcatagagttctcagttcctaatggtttgtatcatctgtggttcttc

200 V Q R S I E D Q T I L V A T Y E G E H N

1741 agGTTCAAAGAAGCATTGAAGACCAAACTATTCTAGTAGCAACATATGAAGGAGAGCACA

220 H P H P S Q M E A T S G A S R S L T L G

1801 ATCATCCACATCCATCACAAATGGAGGCAACATCAGGCGCAAGCCGAAGCCTGACTCTTG

240 S V P C S A S L G S S G P T I T L D L T

1861 GCTCAGTACCCTGCTCAGCCTCCCTTGGATCGTCCGGACCGACCATTACTCTTGACCTGA

260 K S K S S N D A R S S K P R M E T P E V

1921 CCAAATCCAAGTCCAGCAATGACGCCAGAAGTTCAAAACCAAGAATGGAAACTCCTGAAG

280 R Q F L V E Q M A S S L T K D P N F T A

1981 TAAGGCAGTTCTTGGTAGAACAGATGGCCTCTTCGTTGACAAAAGATCCTAATTTCACAG

300 A L A A A I S G K M L Q Q N H K D K W *

2041 CAGCATTAGCAGCAGCCATCTCAGGAAAAATGTTGCAGCAGAATCACAAAGACAAGTGG**T**

2101 **GA**agatttctcagtttaggccattttgaaatttggaatatgtagttgtacatacaacatc

2161 aggaaaaggggaaacacaattttcataaatttgctttgaattgctatttaaggaggaagt

2221 atattgtcattggaaatgattgatgaaaaattcatccccaattttgcaagacgcaattgg

2281 ggtggagttcatgccatattgaattgatcttcaacatttttttttcctttcttggatt

2341 tattttagttattagttccttttaattttaacattaatagtttctctagcaatat

(B)

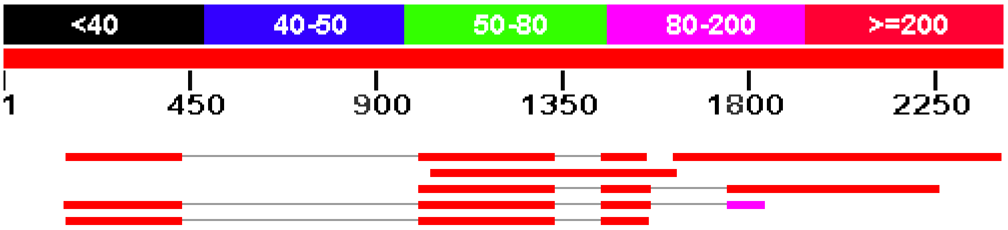

Supplement: S12 File — (PDF) [file pone.0148243.s012.pdf]
