# Supplementary material for: Gene Structures, Evolution and Transcriptional Profiling of the WRKY Gene Family in Castor Bean (Ricinus communis L.)
Source: PLoS One. 2016 Feb 5;11(2):e0148243. doi: 10.1371/journal.pone.0148243 (PMC4743969; doi:10.1371/journal.pone.0148243)
Supplement: S13 File — (PDF) [file pone.0148243.s013.pdf]

S13 File. The gene model for *RcWRKY29*. The coding region is marked with uppercase letters, above which is its deduced amino acids. The transcribed untranslated regions, including 5’ UTR, intron and 3’ UTR sequences, are marked with lowercase letters. The start and stop codons are blacked and the misannotated sequence is boxed.

1 tgtgactttccttctcatccaattgccaattgccaattgctatctttccctcttctatat
61 attcaacttttcttgatatgttttctttcattctcttgaccatcttctttcaaagacttg
121 taatctaatctaaagctattacgtgagaagctaagctatactgttatatctctctccttc
1 M D S A S W V N T S L
181 tttcccaattctcggaagagtttcagttc**ATG**GATTCTGCTTCCTGGGTTAATACTTCTC
12 D L N V N S T F K H I D S V P
241 TCGATCTCAACGTCAATAGTACTTTCAAGCATATTGATTCAGTTCCGgtaagttgattaa
301 tcttaatctttctattgaatgtttcaaatttcttttctttttgatctgaattgtctcac
27 K R E L L A V D F S G L
361 tcctgaatttcttgcacatgttcagAAGAGAGAATTGTTGGCAGTAGACTTTTCTGGGCT
39 D P K V P I K E E K P F L D P Q T S V L
421 TGATCCGAAAGTACCAATTAAGGAAGAGAAACCTTTTCTTGATCCACAGACTAGCGTTTT
59 V E E L T R I S T E N K K L T Q M L T V
481 GGTTGAAGAGCTTACCAGAATAAGCACCGAGAACAAGAAGCTCACTCAAATGCTAACTGT
79 L C E N Y N V L Q N H L A D L M S K N S
541 TCTATGTGAAAATTACAATGTTTTGCAAAACCATTTGGCTGATTTGATGTCCAAGAATTC
99 D K E I I A P S S S R K R K A A E S E D
601 TGATAAAGAAATCATCGCCCCATCATCATCAAGGAAGAGAAAGGCTGCTGAGAGCGAGGA
119 Y S N I I S G A N N I N N G T T E S T S
661 TTATAGTAATATCATCAGTGGAGCCAATAACATTAATAATGGAACTACAGAAAGCACCTC
139 S D E L E S G T K R T K E I K T K I S S
721 TAGCGATGAGTTAGAATCAGGTACTAAAAGGACTAAGGAAATCAAAACAAAGATTTCAAG
159 A Y L R T D P S D T S L
781 CGCTTATCTTCGGACCGATCCATCAGACACAAGCCTGgtaagttccctcatttacccata
841 tcttttctttcttgcgattaagtcttatttttgttagaagtagtattaacagaccaactg
171 V V K D G Y Q W R K Y G Q K
901 gtgcatatatcgtattagGTTGTGAAGGATGGATATCAATGGAGGAAGTATGGCCAAAAA
185 V T R D N P S P R A Y Y K C S F A P S C
961 GTTACTAGAGACAACCCTTCTCCTAGGGCTTACTATAAGTGCTCATTTGCCCCAAGCTGC
205 P V R K K
1021 CCTGTCAGAAAGAAGgtaacagcaacaatatccctcttaattattgcttgtagtcatnnn
1081 nnnnnnnnnnnnnnnnnnnnnnnnnnnnnnnnnnnnnnnnnnnnnnnnnnnnnnnnnnnn
1141 nnnnnnnnnnnnnnnnnnnnnnnnnnnnnnnnnnnnnnnnnnnnnnnnnnnnnnnnnnnn
1201 nnnnnnnnnnnnnnnnnnnnnnnnnnnnnnnnnnnnnnnnnnnnnnnnnnnnnnnnnnnn
1261 nnnnnnnnnnnnnnnnnnnnnnnnnnnnnnnnnnnnnnnnnnnnnnnnnnnnnnnnnnnn
1321 nnnnnnnnnnnnnnnnnnnnnnnnnnnnnnnnnnnnnnnnnnnnnnnnnnnnnnnnnnnn
1381 nnnnnnnnnnnnnnnnnnnnnnnnnnnnnnnnnnnnnnnnnnnnnnnnnnnnnnnnnnnn
210 V Q R S A E D P S

1441 nnnnnnnnnnnnnnnnnnnnnnnnnnnnnnnagGTGCAAAGAAGCGCTGAAGATCCATCA

219 I L V A T Y E G E H N H A R L S Q P E F

1501 ATCTTAGTAGCAACTTATGAAGGAGAGCACAACCACGCGCGCCTTTCACAACCCGAATTT

239 S L G P N R C N S N S V P V P N S T S S

1561 TCTTTAGGCCCAAACCGATGCAATTCAAATTCAGTTCCAGTTCCCAACAGTACTTCATCA

259 T P M M K V P P A S V T L D L M Q D S N

1621 ACACCTATGATGAAAGTTCCTCCAGCTTCTGTAACTCTTGACTTGATGCAAGACAGTAAT

279 A R K A I Q E S E S P A P I Q Q I L V Q

1681 GCTAGAAAGGCAATCCAAGAATCTGAATCTCCAGCTCCAATCCAACAGATTCTGGTGCAA

299 Q M A S S L T R D P N F T A A L A A A I

1741 CAAATGGCTTCCTCATTGACAAGAGATCCAAATTTCACGGCAGCACTTGCAGCTGCTATT

319 S S R F S K T P I Q K W *

1801 TCAAGCAGATTCAGCAAGACTCCGATCCAGAAATGG**TGA**aattaatagtagaattaagag

1861 ttttacttttgtctttgaattttttttttttgaagttacttagagagtacgaagtgatgta

1921 tatagaagatggacactgattctaaatttcag
