# Supplementary material for: Gene Structures, Evolution and Transcriptional Profiling of the WRKY Gene Family in Castor Bean (Ricinus communis L.)
Source: PLoS One. 2016 Feb 5;11(2):e0148243. doi: 10.1371/journal.pone.0148243 (PMC4743969; doi:10.1371/journal.pone.0148243)
Supplement: S14 File — (PDF) [file pone.0148243.s014.pdf]

S14 File. The gene model for *RcWRKY30*. The coding region is marked with uppercase letters, above which is its deduced amino acids. The transcribed untranslated regions, including 5’ UTR, intron and 3’ UTR sequences, are marked with lowercase letters. The start and stop codons are blacked and the misannotated sequences are boxed.

```
   1                                                     M  D  S  H  S
   1 accctctccttttctctaatactcaagtcttattaagtaaaatcatATGGATTCTCATTC
   6  Y  T  K  H  K
  61 ATATACCAAACACAAGgtaaactttcttacttacttcccttttctctcactcactgctct
  11                                                           M
 121 ctctagtctgcatgtctgagtttttctttttaaatcttatgtttcttctgatcagATG
  12 E  S  L  Q  D  E  L  E  R  T  Q  K  E  N  E  T  L  R  F  M
 181 GAGTCTCTACAAGATGAGCTGGAACGTACCCAGAAAGAGAACGAAACTCTTAGATTTATG
  32 L  E  V  M  S  R  K  F  S  T  L  Q  A  N  F  Q  E  K  K  V
 241 CTTGAAGTTATGAGCAGGAAATTCAGCACCCTTCAAGCTAACTTTCAAGAAAAGAAGGTA
  52 Q  E  T  P  S  S  S  C  Y  E  V  Y  E  S  N  K  R  P  R  I
 301 CAAGAAACACCGAGTAGTTCATGCTATGAGGTGTATGAATCCAATAAGAGACCAAGAATT
  72 E  I  P  L  S  K  P  S  Q  I  F  V  R  T  D  S  K  D  K  S
 361 GAGATTCCTTTGTCCAAGCCTTCACAAATCTTTGTTAGAACTGACTCAAAAGACAAGAGC
  92 L
 421 CTTgtaagcttacgttcttttactttgaaaaaaaaatgcatgtttaagccttgattcagg
  93                                        I  V  R  D  G  Y  Q  W  R
 481 ttttcattttagttctctttttggatttgttgcagATTGTGAGAGATGGTTATCAATGGA
 102  K  Y  G  Q  K  V  T  K  D  N  P  S  P  R  A  Y  F  R  C  S
 541 GAAAGTACGGCCAGAAGGTTACTAAAGATAATCCATCACCTAGAGCTTACTTCAGGTGTT
 122  M  A  P  G  C  P  V  K  K  K
 601 CCATGGCCCCTGGTTGCCCTGTCAAGAAGAAGgtatgccattcacaagctttatttctct
 661 ctctctctctctctctctctctctctctgtttaattagggttcaattatgctagggct
 721 tgaatttcatagtctagagatgacttagtaccactgagtcaaactcatcgttattcacaa
 781 tgtttctttcaaggaagtaaaatcaaaattctccgactattattttatgaaacatgcatg
 132                                                   V  Q  R  C
 841 actaattaagaccgagtgtaaaagtcttgtttcttgggttttaattagGTGCAAAGGTGT
 136 A  E  D  K  S  I  L  V  A  T  Y  E  G  E  H  N  H  E  P  N
 901 GCAGAAGACAAGTCAATACTAGTTGCAACTTATGAAGGAGAACACAACCATGAACCCAAT
 156 D  S  P  A  Q  S  V  Y  S  P  D  S  S  S  R  S  S  R  T  T
 961 GATTCACCTGCACAGTCTGTATATTCTCCTGATAGTTCATCAAGAAGCTCAAGGACTACT
 176 I  S  F  Q  P  A  A  V  G  L  D  L  T  L  S  S  S  I  H  D
1021 ATTTCTTTTCAACCAGCTGCTGTTGGCCTTGATCTTACTCTCTCAAGTTCCATTCATGAT
 196 K  R  R  N  F  L  R  V  S  R  K  T  T  N  A  A  T  S  I  V
1081 AAAAGGAGAAACTTTCTCAGAGTCTCAAGGAAGACAACAAATGCAGCAACTTCAATAGTG
 216 V  R  L  K  N  M  W  H  L  S  P  K  I  P  T  L  L  L  L  W
1141 GTAAGGTTGAAGAATATGTGGCATCTCTCACCAAAGATCCCAACTTTACTGTTGCTGTGG
 236 L  L  L  L  Q  G  L  *
```

1201 CTGCTGCTGTTGCAAGGTCTA**TGA**gtggccagctatatagtccaagactactgtgaagat
1261 ctcggtaccttatgttatcatgtatttcactttagatgtaaagggaattgatgaacaaga
1321 tcaattagctttttagctattcttcgcctggccacttagtgttgcatgtaactttgctta
1381 tgcttcgagctactgaatatgag
