# Supplementary material for: Gene Structures, Evolution and Transcriptional Profiling of the WRKY Gene Family in Castor Bean (Ricinus communis L.)
Source: PLoS One. 2016 Feb 5;11(2):e0148243. doi: 10.1371/journal.pone.0148243 (PMC4743969; doi:10.1371/journal.pone.0148243)
Supplement: S15 File — (PDF) [file pone.0148243.s015.pdf]

S15 File. The gene model for *RcWRKY35*. The coding region is marked with uppercase letters, above which is its deduced amino acids. The transcribed untranslated regions, including 5’ UTR, intron and 3’ UTR sequences, are marked with lowercase letters. The start and stop codons are blacked and the misannotated sequences are boxed.

```
   1 ttgagatggagtctccccatccactaacattttatgaagaattatcatctgaattgcaag
  61 ggaaagggctacgtcaaattatgtaagacattttcaagaacttaatgcatgaaattctga
 121 gaatgttacccagtaaatatcatgccctaaaaacttcaggcggccaaaaaaaaaagcagt
 181 cgcaggatctacatgaaattctgagaatgttacccattgaggtaacggcggctatttaaa
 241 tttgaccaagcattggctgtttacacatatatgaatcttgaacatgactactggttcaag
 301 gtttttcctctttctacatgcacaaacgttgaaattgagcatttctttcttacttttaat
 361 caaaattttcttttctcagaacttcaattaaaaaaaaaaattcaataagaaatatgacct
 421 tctctctttggagtatatgtgtttgacatttctcaacgagagtaacatgcataatagata
 481 agtactcgagtttgaatatctattttctttctttttttaagtataaaagtcagaatctgga
   1                                                 M  A  N  K
 541 ggctctattatgtgtatataatgacccagcctagctactaaatgggcatATGGCAAATAA
   5 R  I  S  S  I  S  M  E  T  D  K  S  G  S  L  E  K  P  D  H
 601 AAGAATAAGCTCAATATCTATGGAGACCGACAAGAGTGGTAGCTTGGAGAAGCCTGATCA
  25 C  A  N  T  A  A  V  Q  E  E  K  K  M  K  S  S  G  D  D  E
 661 TTGTGCAAATACTGCTGCAGTACAGGAAGAGAAGAAAATGAAATCAAGCGGTGATGATGA
  45 E  V  V  V  A  E  I  N  K
 721 GGAGGTTGTTGTTGCAGAAATCAACAAGgtgattatgtctttagttaattaggacctgag
 781 tgcatggaagattttagatatgtttatatgctattgtgttctttttcttgggaagttttc
 841 tcttgtcttgttgattagctttccatgcataggaatccacaaaggaactaaaaagattta
 901 tccattaaaaagaaagaagaaaaatctccaaatgaaatcttccttttccttcacttaaat
 961 tcttggaaatacacatagcgaatgatttgtcttttttggattaattaaatctggtatcat
1021 atacttatagttttctccatgacaagatccactttctagaaaattagtttccaagttca
1081 tccattaatgactcttcccatgttttgactctttgcttgaatattgattttcttaattct
1141 ttttcagtaatatacacaaaatgcttttgaagcttatgtatattgaaatgaaatcattat
1201 tcacgttatttcagaaatatttatcttatcaagcaatattcatgtttttttttttcctttt
  54                  E  E  G  D  V  N  H  V  I  N  I  K  Q  E  E
1261 ggtgtatgccatcttagGAAGAAGGAGATGTGAATCATGTGATAAATATTAAACAAGAAG
  69   N  I  R  K  R  P  S  S  T  M  N  K  D  L  T  S  I  N  H
1321 AGAATATCAGAAAGCGGCCTTCTTCCACGATGAATAAAGATCTTACTTCTATCAATCATg
1381 taagaattttcatatacttgtttcgttttatatgtaaacaccttaatttttcttttcact
1441 attattactttggttgttgggcaaagacttgtatgtaaagaaaaatgtgttcttccactc
1501 atatgtgcaagttgatctataacatattaatagtttgattatttcttataataattaagt
1561 tcacttaattttgaagaatttatttcgataagttttccttaaactattgagttgttcaac
1621 caaaatgatggtagaccaattctgtaaatcctaatattgaatattaattattgaggagtt
1681 ttgctttcaaaagatgatcactgcattattaattgatctattctaaacatttttgtgcta
1741 taccgagtcaaacattgttcacaaatcccaccatgaagatcagatctcctcttaattagt
1801 catttgcagaaattgactgagatctattgttattattttatcttcattatatatattcta
1861 gttagtacctttttcttttcaaaaaagaaaatcataaaagcactcagactgcataaatttg
```

1921 aattcttaaatataaagtagtattgggtttattggttatggtcttaatttctagtcttga
1981 ttcctttgcttggttttacaattttagtttgattttgattattgtagtctgctattttgt
2041 ttctcattttcaatgtgaaatttgctgcccatattttacaagtagccacttcatgatta
2101 gaatattatctcaataataattattcgatgccatattatgtaactaaacctatagaattc
2161 agatgttacatttttctgaagctcttagttaagtcgtgtagtctggagatggactaactc
2221 aatgcaagaaactttcccaaagattactattattattatttttattattttagaagtctt
88 E D Q L R S T K A K
2281 aaatcatttagttcacttcttttgacaccagGAGGATCAGCTGAGGTCCACAAAAGCTAA
98 I G E V K E E N E R L K Q L L S K I L N
2341 AATTGGTGAGGTTAAAGAAGAGAACGAAAGATTAAAACAGTTACTATCCAAAATTTTGAA
118 D Y Q S L Q K H F C K V V Q E E E E K K
2401 CGATTACCAATCTCTACAGAAACATTTCTGTAAAGTTGTTCAGGAGGAAGAAGAAAAAAA
138 P A K L T T A H Q K N Q E P E L V S L S
2461 ACCCGCCAAACTAACGACTGCCCATCAAAAAAATCAGGAACCAGAGCTCGTCTCTCTCAG
158 L G R S S S S E P K K E E K K S S N L S
2521 CCTTGGTAGAAGTAGTTCCAGTGAACCCAAAAAGGAAGAGAAAAAGAGCAGCAATCTAAG
178 D G N E D D E L N N K G L S L G L D C K
2581 TGATGGGAATGAAGATGATGAACTGAATAATAAAGGACTTTCTCTTGGTTTGGACTGCAA
198 F E P D S S V T V K N N A S S E N S F D
2641 GTTCGAGCCAGATTCATCTGTAACTGTGAAGAATAATGCAAGCTCCGAAAATAGCTTTGA
218 E D P K E E E P T E T W S P N K I R K T
2701 TGAAGATCCAAAGGAAGAGGAACCTACTGAGACATGGTCACCAAACAAAATCAGGAAGAC
238 T I T P D D E A M Q Q N Q I K K T R V S
2761 AACCATAACTCCAGATGATGAAGCGATGCAACAAAATCAGATCAAGAAAACTAGGGTTTC
258 V R A R C D T P T
2821 AGTCAGAGCCAGATGTGATACTCCAACGgttagcatctaacttttatttattatatatca
2881 tgcactggaatgcatgcatgtgcggcatcaaaaaatactgtttaagggttaactcagttg
2941 gtacttcttattttacgtgattgttttcaaattctgagattggatcattattaggagtta
3001 attttcttacttgtggtctcaatgtaaatgatttgactaattggtaaattaaaaaaaaaa
3061 agtaataggaatattttcatattaagaaaaaaagtaatgaatttgtttttttcaatgaatt
267 M N D G C Q W R K Y G Q K I A K G
3121 ataatacagATGAATGATGGATGTCAATGGAGAAAATATGGACAAAAGATAGCGAAAGGA
284 N P C P R A Y Y R C T A S P T C P V R K
3181 AATCCGTGCCCTAGGGCTTACTATCGTTGCACAGCCTCACCAACTTGTCCAGTGAGAAAA
304 Q
3241 CAGgtatgttttctgcatgccattgttgacatttcaatgattaaaatatttgtttggttt
3301 gtttgaaccatcgatatatgattatgtcctagctacaaccatcaattttagtagaaatta
3361 cagtcaaatatatgacataatgtacttagaaaatagaacttttaggagattatttattta
3421 acatcaactgtcatcatatggaaaagtgcaagaatgcatcatatatatgtcggtaatatc
3481 aatataatgtataagtttttgacatattgttagaaaatacgtaccatcaaactaatgctc
3541 attacttgacatttgatataagtataaagattatattttccaaaatctagttaagttgct
3601 ccaatgtaaatttgatttttatctcgtactatttgaatcaacacatgtaaaatgatttac
3661 ctggtacaaatgataatttaattatacgaaaactccgactttggtattcagtttctcatg
305 V Q R C A K D M S V L I T

3721 cagctttttcttgaatttcagGTGCAAAGATGTGCTAAAGATATGTCAGTCTTGATCACC
318 T Y E G T H N H P L P L S A T A M A S T
3781 ACATATGAAGGAACCCACAACCATCCACTTCCACTTTCAGCAACGGCAATGGCTTCCACA
338 T S A A A S M I Q S R S S T S A Q P G S
3841 ACATCTGCAGCTGCATCCATGATACAGTCACGTTCATCAACATCAGCACAGCCAGGCTCA
358 S I S A P S S I S T S N G L N F S L S Q
3901 TCGATCTCTGCACCAAGTTCAATCTCAACTTCAAATGGACTAAACTTCAGTCTCTCCCAA
378 N S R P Q Q I Y F P N S S I S T S N S H
3961 AACTCAAGACCCCAACAAATCTATTTCCCGAACTCTTCAATCTCAACTTCCAATTCGCAC
398 P T V T L D L T T A P S T T T A Q Y F N
4021 CCCACTGTTACTCTTGACCTCACTACTGCACCATCCACAACTACTGCCCAATATTTCAAC
418 R F S S A P R C L N F S S S P S S T S L
4081 AGATTTTCTTCTGCTCCAAGATGTCTCAACTTTTCTTCTTCACCATCATCTACTTCTCTA
438 D Q S N I N T L Q S L W N P S S Y S T Y
4141 GATCAATCCAACATTAATACTCTGCAATCACTATGGAACCCTTCAAGCTACTCCACTTAT
458 G T V P L N R N Y V E K Q P T P G N H H
4201 GGAACAGTACCTCTAAACAGAAACTACGTAGAAAAGCAACCAACTCCAGGTAATCATCAT
478 V Y Q P Y M H I I N S E T T P P P N Q Q
4261 GTTTACCAGCCATACATGCATATAATTAACAGCGAGACTACTCCTCCTCCTAATCAACAA
498 S L T E S I A A A T K M I T S N P N F R
4321 TCTTTGACAGAGAGCATTGCAGCTGCAACAAAGATGATTACTTCAAATCCCAATTTCAGG
518 S A L A A A I T S F V G N N G G S T T T
4381 TCAGCCTTGGCAGCTGCAATAACATCATTTGTTGGTAATAATGGAGGGAGTACTACTACT
538 T T T T T N S N N S N N P G A G E H Y S
4441 ACTACTACTACTACTAATAGTAATAATAGTAATAATCCTGGTGCTGGAGAGCATTATTCT
558 G L N L K L G D H H Q S L A F N T L Y P
4501 GGCCTGAACTTGAAGTTGGGTGATCATCATCAGTCATTAGCATTTAACACACTTTATCCA
578 S S N S N G I G C A S S Y L N K S A T S
4561 AGCAGCAATAGTAATGGAATAGGGTGTGCTTCAAGCTACCTGAATAAATCGGCTACCAGT
598 L S S Q Q G S L V L F P G S L P F S S A
4621 TTAAGCTCTCAACAAGGGAGCTTGGTTTTGTTTCCAGGTTCATTGCCATTTTCTTCTGCT
618 T R S A S E S P A N S R D H N K G *
4681 ACCAGGAGTGCTTCTGAATCTCCTGCTAATAGTAGGGACCATAACAAGGGT**TAA**ggtatg
4741 tacagagaaattaatatgtaaattttgctgcactttgtttagctagatcgattgatattt
4801 tctgtgtaaaattgtagagatgtttccatatgtatttctgctacaaattgtacattgatt
4861 tttctttttctctctaaagaaatttctggttttggactaaacttgccctatatatatata
4921 tataacatgaccaataaataagtacggattataaatatatataagcaaaacaattaaatt
4981 gatataaaagggatcaatcaattcatgaagtttgtttttcattagaatcaaattggatga
5041 atgattcgacaagataaaatatagattattatacattcatcatttgatttgattgtccag
5101 ctaaacataactaagagaagtcgtttatgaaacaagccaattcaaacactagccgccaac
5161 aatcaatttcatatatttgagatgttcagctgaataatccaggttgggcctaggctattg
5221 atgctggttttctgcacaaaaaaaaaagaaaaaagaaaaaagttcagagagttgaacata
5281 tatagtgttcacttcagcagcagctttaatgatcaatctgtaattaataattagggtttc
5341 tgaacggtt
