# Supplementary material for: Gene Structures, Evolution and Transcriptional Profiling of the WRKY Gene Family in Castor Bean (Ricinus communis L.)
Source: PLoS One. 2016 Feb 5;11(2):e0148243. doi: 10.1371/journal.pone.0148243 (PMC4743969; doi:10.1371/journal.pone.0148243)
Supplement: S16 File — (PDF) [file pone.0148243.s016.pdf]

S16 File. The gene model for *RcWRKY40*. The coding region is marked with uppercase letters, above which is its deduced amino acids. The transcribed untranslated regions, including 5' UTR, intron and 3' UTR sequences, are marked with lowercase letters. The start and stop codons are blacked and the misannotated sequence is boxed.

1 cttttactacttttcttttattttatctttttatttttaagtatgatgatttgtttatgt
61 tgttttcgagttaaagagagcctttgttttcttttgggtcattttagcaaaatccaagaa
121 agagagacagctctagtcttctctttcacaccatggtatagtctgcataatctcactgat
181 caacttagcaaggtcttttttttattttttcgctttggtcattgttgtctgttctttttc
241 tgtttttctattgttttagccaagcttagttctatcctattataagttattagtgacgag
301 aaaaggctaaatttttttgggattattattatttttatggatagtttttatttcagtatgg
361 tggttttaaactctcactctgcttggaagaagaactttttggtgatgcaaaaattttgag
421 atttgagcaatagttatttgggttttgccatttgtggaaatgggttttgtgtgttttgtt
481 ttgtagtggtagtttgaactctcacttgctactgagagtttaaagggttttaaaaactga
1 M E E V E E A N K A A
541 ggttttggtttcttgagttttggtgatt**ATG**GAGGAGGTTGAGGAGGCAAATAAGGCAGC
12 V E S C R R V I A L L C Q P R D Q V Q A
601 TGTTGAGAGCTGCCGTAGAGTTATTGCTCTCCTATGTCAACCTAGAGATCAAGTTCAGGC
32 R N L V T E T G E T V S K F K R V V S L
661 TAGAAACTTGGTGACGGAAACTGGAGAAACTGTGTCTAAGTTTAAAAGAGTGGTGTCTCT
52 L S N S L G H G K V R K L K K F K S S L
721 GTTGAGTAATAGTTTAGGTCATGGAAAAGTGAGAAAGTTGAAGAAGTTTAAATCATCTTT
72 P Q N I F L D S P N C T T I L A P K P L
781 GCCTCAAAACATCTTTCTAGATAGTCCTAATTGCACAACAATTTTGGCACCGAAACCCCT
92 Q M V P A S F L E T P I S E M D S K S K
841 CCAAATGGTTCCTGCTAGTTTTCTCGAGACCCCTATTTCTGAAATGGATTCCAAATCTAA
112 S S M Q P T R K I F L E N S V V E M S S
901 ATCTAGTATGCAACCTACCCGGAAAATTTTTCTTGAAAATTCAGTTGTTGAAATGAGCTC
132 N V R P S F Q I S Q I K P T Q Q Y Q F L
961 AAATGTCAGGCCTTCTTTCCAGATTTCTCAAATAAAACCAACACAACAATACCAGTTTCT
152 Q Q Q Q Q I Q R M Q F Q Q Q Q Q M K Y Q
1021 TCAACAACAACAGCAAATACAAAGGATGCAGTTTCAGCAGCAGCAACAAATGAAATATCA
172 A D M M Y S R S N S G I N L K F D G S T
1081 AGCTGATATGATGTATTCTAGGAGTAACAGTGGGATTAATCTTAAATTCGATGGGTCTAC
192 C T P A M S T T R S F I S S L S M D G T
1141 CTGCACACCAGCCATGTCAACCACAAGATCATTCATATCATCTTTAAGCATGGATGGCAC
212 V T N F D R D S F H L I G V P Q S S D Q
1201 CGTGACTAACTTTGATAGAGATTCATTCCATCTGATTGGTGTACCTCAGTCATCTGATCA
232 I S Q Q T R R R C S V R G E D G S V K C
1261 GATCTCGCAGCAGACAAGACGAAGGTGTTCTGTTAGAGGGGAAGATGGCAGTGTTAAATG
252 A S S G K C H C S K R R
1321 CGCCAGCTCCGGTAAATGTCATTGTTCAAAGAGAAGgtcaaactcaaatctctgttagtt
1381 tctaattctggttcttttcttttaaaattataattctgatccaaaatacttttgttttag

264 K L R V K R S I K V P A I S N K V A D I

1441 GAAACTGAGAGTGAAGAGATCCATAAAGGTGCCTGCTATTAGTAATAAGGTGGCAGATAT

284 P P D E Y S W R K Y G Q K P I K G S P H

1501 TCCTCCTGATGAGTATTCATGGAGAAAATATGGGCAAAAGCCGATTAAGGGCTCTCCACA

304 P R

1561 TCCTAGgtatttatctaattcaaccttttattataagttttaagatacatataccctgtg

1621 atttatttatttgtcatgttctaagattgttcctgtgcatgcataacagtaatctacctt

1681 aagtagttggttaattgcttaaatacaaattgaatgacgcatgttaagagatatttggaa

1741 acaagaaccttagctgtctttactttctgcaactcttaattggttaggtaactccgtttg

306 G Y Y K C S S L R

1801 tctgacacttttgaaactccacttttactgcagGGGATACTACAAATGTAGCAGCCTTAG

315 G C P A R K H V E R C L E D P S M L I V

1861 AGGTTGCCCAGCAAGGAAGCATGTCGAGAGATGCCTGGAAGACCCTTCAATGCTAATTGT

335 T Y E G E H N H S R L L S T Q S A H T *

1921 CACTTATGAAGGTGAGCATAATCATTCCAGATTACTCTCGACACAATCTGCCCATACA**TA**

1981 **A**aaactttgcagatgtcttcagtcctctctccaagaattcaggattccaaaaatgtttcg

2041 aatccaacccggaatcggctggcgcttatttgtgaattactactgtatataatattgttg

2101 ttcgaaccagagactagcaatggtggagcagattttatgattagatgacagggggttagt

2161 ccggtttgagctgcttttattatacagaatagggattaaaaattgtaaacttatgcttcc

2221 ctatatatattgaagttgtgtttcttctgcaaacccatggatttctaatcaaaagcaacg

2281 cctgttaggctaggtttttgttctaatttaatattatgcttctataggcagcatcagcc

2341 attgtaggtgcaatat
