# Supplementary material for: Gene Structures, Evolution and Transcriptional Profiling of the WRKY Gene Family in Castor Bean (Ricinus communis L.)
Source: PLoS One. 2016 Feb 5;11(2):e0148243. doi: 10.1371/journal.pone.0148243 (PMC4743969; doi:10.1371/journal.pone.0148243)
Supplement: S17 File — (PDF) [file pone.0148243.s017.pdf]

S17 File. The gene model for *RcWRKY41*. The coding region is marked with uppercase letters, above which is its deduced amino acids. The transcribed untranslated regions, including 5' UTR, intron and 3' UTR sequences, are marked with lowercase letters. The start and stop codons are blacked and the misassembled "TCTTGCTCCAGAAGAG" sequence is boxed.

1 cagtgacatgctttaaatcttacacagccgatgccatcattttgaccagactgcagagga
61 gaggaagaaagaaagactcttcgccctttctcttctcgtttatcaacagcctactcccct
121 tcatttttctcttccatagctctgctctctgttctctctctctctctctctcttttcatg
1 M A V D L M M G Y S R T N
181 ttcttgtttcaccatcacagtg**ATG**GCTGTTGATCTCATGATGGGTTACAGCAGAACCAA
14 S F V S K T M E E N A V V Q E A A S G L
241 TAGTTTTGTATCTAAAACTATGGAAGAAAACGCAGTTGTTCAAGAAGCAGCTTCAGGGTT
34 E S V N K L I K L L S Q Q N Q D K L I H
301 AGAGAGTGTGAACAAGTTAATAAAGTTACTTTCACAGCAGAATCAAGATAAATTAATTCA
54 Q S S S S S S P P C S T M D I E I D C K
361 TCAATCATCATCTTCATCATCTCCTCCTTGCTCAACCATGGATATTGAGATTGACTGCAA
74 A V A D A A V S K F K K V I S L L G R T
421 AGCAGTAGCTGATGCTGCTGTGTCTAAATTCAAGAAAGTCATTTCTCTTCTTGGTAGAAC
94 R T G H A R F R R A P V S T P L I S T S
481 AAGAACCGGTCATGCTCGCTTTAGAAGAGCTCCTGTATCTACTCCTTTAATAAGCACTAG
114 Q D Q E N H H Q Y Q I L E S K V Y Y A T
541 TCAAGATCAAGAAAATCATCATCAATATCAAATTCTTGAAAGTAAGGTCTATTACGCTAC
134 P I Q Q V P P P P P P P P P P P P P S Y
601 GCCGATCCAGCAGGTTCCACCTCCACCGCCACCGCCACCGCCACCGCCACCGCCTTCTTA
154 H D Y S S M V I M P K N N H N N S N N G
661 CCATGATTATTCTTCTATGGTGATTATGCCAAAGAATAATCATAACAATAGTAATAATGG
174 V T N D R K E S S T T I N F S Y S S A A
721 AGTTACTAATGACAGAAAAGAATCGTCAACTACCATTAATTTTTCTTATTCTTCTGCTGC
194 N S F V S S L T G D T N D S K P S P S S
781 AAACTCATTCGTGTCTTCTTTGACGGGTGATACTAATGACAGTAAACCATCACCTTCATC
214 A F Q I T N L S Q V S S A V K P P L S T
841 AGCTTTCCAGATTACTAATCTTTCTCAGGTTTCTTCCGCTGTGAAGCCTCCTTTGTCTAC
234 S S L K R K C S S E N L G S G K C G S S
901 TTCTTCTTTGAAGAGGAAGTGTAGCTCTGAAAATCTGGGTTCTGGCAAGTGTGGCTCTTC
254 G R C H C S K K R
961 TGGTCGATGTCATTGCTCCAAGAAGAGtcttgctccagaagaggtataactttctttcct
1021 tttagttaatcttgatttttatttcagctgtttctggttaatcttgattttttttttcttt
263 K L R L
1081 cctaaattataaattaaatggatcttgatttttcgatttattatagcagAAAGTTGAGAT
267 K R V V R I P A I S L K L S D I P P D D
1141 TGAAGAGAGTGGTCAGAATTCCAGCAATTAGCTTGAAGTTGTCTGATATACCACCGGATG
287 Y S W R K Y G Q K P I K G S P H P R
1201 ATTATTCTTGGAGAAAGTATGGGCAAAAACCCATTAAAGGATCTCCTCATCCAAGgtaaa

1261 ttttcttaaagaataaaaatgatttcataacggttttgatcttgtgactagtttatttaa

1321 gactatgagaagaatataattttggataaattaagtgtaattgggttgtgcattatttat

305 G Y Y K C S S V R G C P A R K H V

1381 gtgaacagAGGATACTACAAGTGCAGTAGTGTGAGAGGATGTCCTGCACGTAAACATGTG

322 E R A S D D P S M L V V T Y E G E H N H

1441 GAAAGAGCTTCAGATGATCCATCAATGCTTGTAGTGACATATGAAGGAGAGCACAATCAT

342 T L S V A E T T N L I L E S S *

1501 ACTCTCTCAGTTGCAGAGACGACCAATCTCATCTTAGAATCATCA**TAG**ataatttaatca

1561 tgaatcccattatcatcatcaacagcaagagtgtaatgaattagctccattgcaagtttg

1621 aaagtaaagaaggtaggcaaatagttgtagtaatatttagtggtagtagtagtggtagct

1681 tgtggtgtggaagaaggacaggactccagactttaaaaaaaaaaaaaaaaaaagggcctc

1741 tctgctttaattatctcgaaggagattatagtgagagttggttcatttcatttaatttct

1801 tttctctttgtaaaatattttggaattaatttgaattccttgatcctttttttctttccct

1861 tt
