# Supplementary material for: Gene Structures, Evolution and Transcriptional Profiling of the WRKY Gene Family in Castor Bean (Ricinus communis L.)
Source: PLoS One. 2016 Feb 5;11(2):e0148243. doi: 10.1371/journal.pone.0148243 (PMC4743969; doi:10.1371/journal.pone.0148243)
Supplement: S18 File — (PDF) [file pone.0148243.s018.pdf]

S18 File. The gene model for *RcWRKY44*. The coding region is marked with uppercase letters, above which is its deduced amino acids. The transcribed untranslated regions, including 5’ UTR, intron and 3’ UTR sequences, are marked with lowercase letters. The start and stop codons are blacked and the misannotated sequence is boxed.

```
   1 ttttttattaaaaactacttcactttgccatcactgtgaaattataattcgttagacatgc
  61 actttttgaatcgattgtttccctttttacaaaagtattagttttgttgaatcattta
 121 ttattaaaacacttaaaccctgcaaattttgagcatgatttgaataaaataaccttagtt
 181 tctactccaccattttctgtcttcttcaattttcttttcaccttctctcatttttctctt
 241 tttctttggatattgtttaatgcttgttttacgattatttcaactgtctattttgtatcc
 301 gaagctgccattgattatctaggcatcttcttggatttgatcagctcacttagttgcttc
 361 tgactcagcttatattgtcagaaacatgatcattcatggctgtctagttcacttcttta
 421 actacttatagctttaatttgccttcctataaggcctttatttataaggatcacgaagga
   1                                M  A  P  R  G  D  F  K  V  H  E
 481 agcatcagcttcaggtgtatgtgtttcaATGGCCCCCAGAGGGGATTTCAAAGTTCATGA
  12  V  A  Q  S  S  F  R  Q  A  H  F  L  F  T  C  I  S  D  K  N
 541 AGTTGCACAAAGTAGTTTCAGGCAAGCTCATTTTCTCTTCACTTGCATTTCTGATAAAAA
  32  Q  K  R  S  I  Q  E  V  S  L  I  A  Q  G  A  V  N  E  F  R
 601 TCAAAAGAGAAGCATTCAAGAAGTGAGCCTGATAGCTCAAGGTGCAGTAAATGAATTCAG
  52  N  L  L  T  L  L  D  G  S  T  Q  S  D  H  P  K  R  I  K  K
 661 AAATCTACTTACTCTTCTTGATGGATCAACACAATCTGATCATCCTAAGAGAATAAAGAA
  72  G  P  L  P  L  S  S  V  K  I  N  P  V  E  L  M  D  S  P  N
 721 AGGTCCTTTGCCACTTTCCAGTGTCAAGATAAACCCTGTCGAATTGATGGATAGTCCCAA
  92  S  M  P  L  I  M  S  S  S  G  C  N  I  R  Q  F  F  P  L  Q
 781 TTCTATGCCTTTGATCATGAGTTCTTCAGGGTGCAATATTAGGCAGTTTTTCCCCCTTCA
 112  T  I  Q  S  A  G  S  V  A  P  T  N  S  F  N  L  Y  V  Q  K
 841 GACTATTCAATCAGCCGGTTCAGTAGCCCCCACAAATAGTTTCAACTTGTATGTGCAAAA
 132  H  K  T  K  T  N  T  D  F  R  N  S  L  V  M  N  S  S  N  P
 901 ACACAAGACTAAAACCAATACTGATTTCAGAAACAGTCTGGTCATGAATTCATCAAACCC
 152  S  P  L  K  P  I  R  T  S  F  L  S  L  D  D  R  S  G  K  S
 961 CTCCCCATTAAAGCCGATAAGAACATCTTTTTTAAGCTTGGATGATAGAAGTGGTAAGAG
 172  K  R  S  V  G  Y  S  S  S  E  I  M  A  S  R  D  D  F  T  M
1021 CAAGAGATCAGTTGGTTATTCATCATCTGAAATTATGGCATCTAGAGATGATTTTACTAT
 192  H  S  S  K  C  K  S  E  I  K  S  E  E  T  N  S  T  K  C  L
1081 GCATAGTTCAAAGTGTAAGAGTGAGATTAAGAGTGAAGAGACAAATAGTACCAAGTGTCT
 212  A  S  T  G  G  C  H  C  S  K  R  R
1141 TGCCTCAACTGGAGGATGTCACTGTTCAAAGAGAAGgtaaattctcatataatttggcat
1201 ggaaatgtttggtttagatattaatggtagttctaatgtaatcttccttaaattatgtgt
 224           K  M  R  I  K  K  I  I  Q  V  P  A  T  S  S  G  K
1261 gatttatagGAAAATGAGAATCAAGAAAATAATTCAAGTTCCTGCTACTTCAAGTGGTAA
 241  L  A  D  I  P  P  D  D  Y  T  W  R  K  Y  G  Q  K  P  I  K
1321 ATTAGCAGACATACCTCCAGACGATTACACTTGGAGGAAATATGGACAGAAACCCATTAA
 261  G  S  P  Y  P  R
```

```
1381 AGGATCTCCTTATCCTAGgtatgtctgtatttttctttaatttttttaaatgaattggtg
1441 tctgtttgatcactaaaatgctttgatatttgccacttctcttggcaaggcacaaggtgt
1501 ttaattctacttattttctttggtaggctgtgggatgggcttaggaaaataaattaaaaa
1561 caaaaggtaacaagaggcaactgcaatttaatagaatagccataggatagattcaccatt
1621 ggacagtcaactggtccaaagtctagtctctgacctcatcaagaagccagcagttttagt
1681 tcctgacatccccaaacatccctcaaaccgatctaaccgagtttaaactggaacaatcct
1741 aaaagaggatgtcaaaactttactattagcctgtcctcttagaggcaccagtaaacatgt
1801 tagacatgggtgttatcttcactcggaccatcctttagaataacagttcaagttgcgggt
1861 tcttaaggtgccccatatttggtttcagtcaattctgggtgctgacatgtttatctttga
 267                  S  Y  Y  K  C  S  S  M  R  G  C  P  A  R  K
1921 taatttgttcgcgaagGAGCTATTACAAATGCAGTAGCATGAGAGGCTGCCCTGCAAGGA
 282  H  V  E  R  C  L  Q  D  P  A  M  L  V  V  T  Y  E  G  D  H
1981 AGCATGTCGAACGATGTCTGCAAGATCCGGCTATGTTAGTCGTCACCTATGAAGGAGACC
 302  S  H  S  K  I  P  L  Q  S  P  N  I  L  I  Q  V  *
2041 ATTCTCACTCCAAAATTCCGCTCCAATCACCCAACATTCTTATTCAGGTTTAActaaagc
2101 aaagcaagttccagtttccttggtaacaagcctgtgttggtgtattattttcttgtgttt
2161 ataagacatgtatgcttctctggtttatataattggaacttcaacttcagtaacagattt
2221 ctacatctgttacgtgtatattcgctcttttgagagtatcttaatgatgttttagattat
2281 g
```
