# Supplementary material for: Gene Structures, Evolution and Transcriptional Profiling of the WRKY Gene Family in Castor Bean (Ricinus communis L.)
Source: PLoS One. 2016 Feb 5;11(2):e0148243. doi: 10.1371/journal.pone.0148243 (PMC4743969; doi:10.1371/journal.pone.0148243)
Supplement: S19 File — (PDF) [file pone.0148243.s019.pdf]

S19 File. The gene model for *RcWRKY50*. The coding region is marked with uppercase letters, above which is its deduced amino acids. The transcribed untranslated regions, including 5' UTR, intron and 3' UTR sequences, are marked with lowercase letters. The start and stop codons are blacked and the misannotated sequences are boxed.

```
   1 ctgaaacgaagaagaagaacaagaacaagaaatgcccagtagttgtaaccttgcagtgag
  61 aaaatccctgtttttgttttgttttgttttatatgcttcttctctcttttctttattcat
 121 cattctttgcttcctatcttagctaataactcaaaaccacaaaagacccatcttctcaaa
 181 acatcaatatcccttaaggatagcaagaaattaaagaaaaagaaaaaccttatcttttta
 241 aaaaaaaatttgcatgcctttattgggtgcaagaaagtacacagataacaccaaattctc
 301 tccttcttactctatctctctctctctctccctttttcttacctatataggtatacatg
 361 tatttggtgtctagatatggattgattctggtgtattagtataatatgtgcagcttcttt
   1           M  D  N  Y  Q  G  D  L  T  D  I  L  R  A  S  G  T
 421 ggcttggaaATGGACAACTACCAAGGTGATTTAACAGATATACTTAGAGCAAGCGGTACC
  18 S  T  G  G  A  L  G  Q  S  D  G  P  A  A  A  L  S  N  W  E
 481 AGCACTGGCGGCGCTTTAGGCCAATCGGATGGACCTGCTGCAGCTCTTTCAAACTGGGAA
  38 F  N  C  D  P  L  G  N  L  S  S  A  S  A  L  E  D  N  S  S
 541 TTCAATTGTGACCCGCTCGGTAATCTTTCTTCAGCTTCGGCTTTGGAAGATAACAGCAGC
  58 S  S  R  D  N  D  N  N  N  N  D  N  N  N  V  F  G  D  P  F
 601 AGCAGCAGAGATAATGATAATAACAACAACGATAATAATAATGTTTTTGGTGATCCTTTT
  78 I  N  M  R  D  P  L  L  H  E  L  N  N  V  V  A  S  G  C  S
 661 ATTAACATGAGAGATCCACTTCTTCATGAGCTCAATAACGTTGTTGCCTCCGGCTGTTCA
  98 A  Y  F  C  S  T  N  S  T  D  H  L  L  S  T  T  A  G  T  S
 721 GCCTATTTTTGCAGCACAAATTCTACAGATCATTTGCTTAGTACTACTGCTGGTACTAGT
 118 I  D  D  A  C  N  F  P  G  A  N  S  R  S  S  H  H  H  H  K
 781 ATTGATGATGCTTGTAATTTTCCTGGTGCAAATAGTAGAAGCAGTCATCATCATCATAAA
 138 V  F  E  E  E  I  N  N  N  N  N  S  N  N  S  P  C  N  I  F
 841 GTTTTTGAAGAGGAGATAAACAATAATAACAATAGCAATAATAGTCCTTGCAATATATTT
 158 S  R  I  Q  I  S  Q  N  N  S  A  A  K  L  P  V  S  P  C  D
 901 TCAAGGATTCAGATCTCCCAGAATAATTCAGCAGCTAAGTTGCCTGTTTCTCCATGCGAT
 178 S  P  V  I  A  A  A  V  S  P  R  G  F  K  A  S  P  M  V  S
 961 TCTCCGGTGATAGCTGCTGCTGTTTCTCCCAGGGGTTTCAAGGCTTCTCCTATGGTTTCA
 198 I  D  M  I  N  T  N  T  S  K  N  C  L  I  D  N  T  G  Q  M
1021 ATTGATATGATTAACACTAATACTTCAAAAAATTGCTTGATTGATAACACTGGACAGATG
 218 Q  I  S  S  P  R  N  L  G  I  K  R  R
1081 CAGATCTCTTCTCCGCGGAATCTGGGCATCAAGAGAAgtgagagtatcgtttaatttaat
1141 ctttctcacttgtttctatgnnnnnnnnnnnnnnnnnnnnnnnnnnnnnnnnnnnnnnnn
1201 nnnnnnnnnnnnnnnnnnnnnnnnnnnnnnnnnnnnnnnnnnnntttgaacttgaattttct
1261 acaaaagaatggtctgaggaattagggtttggcattcaatatgtgtgatgattctcaaga
1321 ttgattgtcttgttcttaggtgtgtgtatatatcatatcaggtgagattgttcactgttt
1381 acattacatgctaaatttacataaaatcttgatgggttctgatcaaaaacctgaaaaggt
1441 atcatctatataactgttgtctttatgttactcacatagcttagctttcatgtacttatc
1501 tcaaactcctaattatttatttatttatttattttacttctttctctttctctcccccca
```

1561 acacgcacctgacctaaatgaaataactgttctttgattagcacttgtctttagaatata
1621 tcacatccctttcatcctgacaaataagccaaattaaggttttcttgttcaagaatggac
1681 taatcatcattgtcattgctagcttaatcagtgtattctcaaatttgatgaaattttgat
1741 attcatcatcgttgtcatctcaaattctttttctttggtcttggataatgtggaaaaggc
231 K S Q A K K V V
1801 aagaaataaacaaaatttggtatttgttccattcagGAAAGAGTCAAGCAAAGAAGGTGG
239 C I P A P A A A N S R P S G E V V P S D
1861 TTTGCATACCAGCACCAGCAGCTGCAAACAGTAGGCCAAGTGGAGAAGTAGTTCCTTCTG
259 L W A W R K Y G Q K P I K G S P Y P R
1921 ATCTGTGGGCATGGAGAAAGTATGGTCAGAAGCCTATCAAGGGTTCTCCTTATCCAAGgt
1981 aagtgatgatagaaaaatttgtattgtcattctagtttaataactataaaaagttcctaa
2041 gacaaagaacaaaagctgaattcctccattccatttcctttcctctgacatatattattc
2101 cattgaaaaaataaaaagaaacatttcttgcttcttttctttttagatcttttactatag
2161 gaattgccattattttcctgtttgagcatgaaatacaacaatttatatacatgtcttcaa
2221 acaatgtcctgtagagtgtgcttctggaccaactaaaatttccctagaaactgaattcaa
2281 acaaattctttcatcaaatactgatcgattagtaatatctttagaaatcagtggagggta
2341 tttcaatttcatttgttaatttctttttgataaaaactctaggagagtttcccaacca
2401 ttgtgcagaatttgcatgagtgaatagatgctgacatttcctgtatttgtaatggtttat
2461 ttccctttctattgtcattttgactgttgaactcggacttgaataagtgaataactacct
2521 aaaaacggcgataaattcagtgatcattataactaactaaataactaactcatgtctcta
278 G Y Y R C S S S K G C S A R
2581 tatataacatattccagAGGGTACTATAGATGCAGTAGCTCAAAGGGTTGCTCAGCAAGA
292 K Q V E R S R T D P N M L V I T Y T S E
2641 AAACAAGTGGAGCGTAGCCGAACTGATCCTAACATGCTAGTCATTACTTACACTTCTGAG
312 H N H P W P T Q R N A L A G S T R S Q P
2701 CACAACCATCCATGGCCAACTCAAAGGAATGCTCTTGCTGGCTCAACAAGGTCACAGCCA
332 P K N N A G S S K S S P T S H P Q K T T
2761 CCAAAGAACAATGCTGGTTCTTCGAAGAGCTCACCGACCTCTCATCCTCAAAAGACAACA
352 L N T K E Q K E S S N D T F S P S N M V
2821 TTAAATACAAAAGAACAAAAGGAAAGCAGCAATGATACATTTTCTCCTAGTAACATGGTT
372 A A A S A S V K E E I E D I H E K H I M
2881 GCAGCTGCCAGTGCATCAGTAAAAGAGGAGATTGAGGATATTCATGAGAAGCATATAATG
392 E M E D A D Q F S S D G F P Q S S S Y R
2941 GAGATGGAGGATGCCGATCAGTTCAGTAGTGATGGCTTTCCTCAGAGTAGTAGTTACAGG
412 P T M P D S S Q S E D F F A D L G E I E
3001 CCTACAATGCCAGATTCTAGTCAATCTGAAGATTTCTTTGCTGATTTGGGAGAGATAGAA
432 A D P L D L L F S Q G G F S G D H E Q K
3061 GCAGACCCTCTTGATCTTTTGTTTAGTCAAGGGGGATTCAGCGGAGATCATGAGCAGAAA
452 G S K G L D P F G L F D W S E D T N N I
3121 GGAAGCAAGGGTTTGGATCCATTTGGTCTTTTTGATTGGTCAGAAGACACCAACAACATC
472 S F E E A K R D L *
3181 TCATTTGAAGAAGCCAAGAGGGATTTA**TAA**caagacctcctttcaactgaaaatagactg
3241 tttgaagaaaacaagtatgtagttatatcttggtacaatctatctgctaaaaaaccttg
3301 tctttttggctgtgaatttcatgtggagaggatgaagaggaagagatggggaaggttttt

3361 cttcttccttctactttcatttaaaatttttctttttcttgttatttaccagaacagtca
3421 gggaagtttagggaaaaagttaaagagtaaaaaaaagacgagaagagagagagagagaga
3481 gagagagagagagagacagtgctggttgattgggagcagaagcaatggagtcccacattc
3541 cttttggaatattggtggatgggacagaaagctgaggaacagaaggtttgaacagttaaa
3601 acaagtacatccatcagacatgactggaaagctcagcaaaggatagattgatacaaggat
3661 gaaagaaagatgggacttgcccttctctctctttcatcttttcttttcaagtgggattaa
3721 ttattcaatccttcactctcctttgatcatgtgggcattctttaaag
