# Supplementary material for: Gene Structures, Evolution and Transcriptional Profiling of the WRKY Gene Family in Castor Bean (Ricinus communis L.)
Source: PLoS One. 2016 Feb 5;11(2):e0148243. doi: 10.1371/journal.pone.0148243 (PMC4743969; doi:10.1371/journal.pone.0148243)
Supplement: S20 File — (PDF) [file pone.0148243.s020.pdf]

**S20 File. The gene model for *RcWRKY54*.** The coding region is marked with uppercase letters, above which is its deduced amino acids. The transcribed untranslated regions, including 5’ UTR, intron and 3’ UTR sequences, are marked with lowercase letters. The start and stop codons are blacked and the misannotated sequence is boxed.

```
   1 tctacgctttctgttcttcttgcaactgatcttgtgatcccattagctttttcagctctt
  61 ttcatctttataactcaacattcttctctttctcttttaagcccaactttcatgtttgtt
 121 gcttttaattcggcttatcagagctttatcttagccttctttttcacttccttctcccac
 181 atagcaatagctctgtttaatatcttctatccaaagcctgggtacaagccacagctgtag
   1                                                        M  E  K
 241 ctgtttcgaagtatatcagtagtttgaacttgtccttggctgcatttttctaaATGGAAA
   4  H  T  L  I  G  E  L  T  Q  G  K  E  L  T  E  Q  L  K  H  H
 301 AGCATACTCTCATCGGTGAACTAACACAAGGGAAGGAGCTCACGGAACAGCTCAAACACC
  24  L  N  P  A  S  S  L  Q  T  R  E  F  L  V  G  K  I  I  S  S
 361 ATCTTAACCCTGCATCGTCACTTCAAACTCGTGAATTCCTTGTTGGAAAGATAATTTCTT
  44  Y  E  K  A  L  S  L  L  N  C  G  A  S  F  D  V  H  H  N  P
 421 CTTATGAGAAAGCACTTTCTTTGCTGAATTGTGGTGCTAGTTTTGATGTTCACCATAACC
  64  N  I  C  I  S  E  S  M  H  P  I  H  K  R  S  P  R  S  E  I
 481 CCAATATTTGCATATCAGAATCGATGCACCCAATTCATAAGCGTAGCCCGAGAAGTGAAA
  84  P  D  E  D  C  K  D  Q  A  Q  K  N  V  F  K  K  R
 541 TCCCTGATGAAGACTGCAAAGACCAAGCTCAGAAAAATGTATTCAAGAAAAGgtaaatta
 601 attagtttgtaatattgagtcattgagctaagctatattttcagttttctttatctgatt
 101                                         K  T  Q  Q  R  C  T
 661 tcgcggttggatattcatcgtatattgtttctttgcagAAAGACACAGCAAAGATGTACA
 108 E  Q  V  K  V  C  S  G  T  G  L  D  G  P  L  G  D  G  Y  S
 721 GAACAAGTGAAGGTGTGTTCAGGAACAGGACTAGATGGTCCGCTTGGTGATGGTTATAGC
 128 W  R  K  Y  G  Q  K  D  I  L  G  A  K  F  P  R
 781 TGGAGGAAATATGGTCAAAAAGATATTCTTGGAGCTAAATTTCCAAGgtgtgtgagatac
 841 tctgttcatctttatcttcacattttaatttcctaagaaaaaagaatagaaaaagtacag
 901 gtggacttgaattatatgcagcagctttgattaaatggttagtgtgtagatgcagcttgg
 961 aacatgaaccagtaataggagcatatgtcactgatattaataaatataatctattggatg
1021 tacgttatgaaaggtaaaaagtaattataggtaaagtttttaagataaaaacacacgtc
1081 tggtagtttttattaacttacttaacagtaccaaagtctgtatgtatgttctctaagaa
1141 gctggaaaggattggcttatttaagtacttccgtttgcatattattatttcttcttgaga
 144                                                        G  Y  Y
1201 aaaagaagtaaagaaaggattgctctaacttggagagttatgcaattacagGGGATATTA
 147  R  C  T  H  R  H  S  Q  G  C  L  A  I  K  Q  V  Q  R  S  D
1261 TAGATGCACTCACCGTCACTCGCAAGGTTGTTTGGCGATAAAGCAAGTACAAAGATCAGA
 167  E  N  P  S  I  F  E  V  T  Y  R  R  K  H  T  C  V  Q  A  S
1321 CGAGAACCCTTCAATTTTTGAAGTGACCTATCGACGAAAGCATACGTGCGTCCAAGCTTC
 187  H  S  A  M  S  N  D  N  P  K  Q  N  Q  G  H  P  Q  L  Q  Q
1381 GCACTCAGCAATGTCTAATGACAACCCTAAACAGAACCAAGGACATCCTCAGCTACAACA
 207  E  E  K  P  I  P  T  T  M  E  E  F  L  N  S  E  G  L  I  V
```

1441 AGAAGAGAAGCCAATACCAACAACAATGGAGGAGTTCCTTAACTCGGAAGGACTTATAGT
227 K T E N L D T G E E I F P S F S F L D A
1501 TAAAACTGAGAATTTGGATACTGGGGAGGAAATCTTTCCATCCTTTTCCTTCCTGGATGC
247 L I D K H N E E N S F V G S L S P T F I
1561 ATTGATCGACAAACATAATGAAGAAAACAGTTTCGTGGGCAGCCTTTCTCCGACATTTAT
267 S P A T S E S N Y F S T S S C Q F D T L
1621 ATCTCCAGCGACTTCCGAGTCCAACTATTTCTCGACGTCGTCTTGCCAGTTTGACACCCT
287 G I G Y N V Q T P E S E L T E I G S V P
1681 AGGAATAGGCTACAATGTGCAGACTCCAGAGTCGGAACTCACGGAGATAGGCTCGGTTCC
307 T S V T N S P I G D F D F S N M D L E T
1741 GACTTCGGTCACTAATTCTCCAATAGGGGATTTTGACTTCTCTAACATGGATCTCGAAAC
327 S F Q F G N L E L E F L A *
1801 CAGTTTCCAATTTGGCAACTTGGAGTTGGAATTCCTTGCTTAGgagtgttagccacatac
1861 tgcattaaacgtatcaatatagccttagaaaggggatagagtagaaattttccagctga
1921 ttaggtagctgaattttggaaataatgtgtagcctgttagtccatttttcctgtttcttt
1981 cttttctttcctcactcaacttattggttatttcaagatgctgcagtgttgtaattattg
2041 tttcacctgtttcttaatcttgaatgtattcataatctcatgcgattcattctc
