# Supplementary material for: Gene Structures, Evolution and Transcriptional Profiling of the WRKY Gene Family in Castor Bean (Ricinus communis L.)
Source: PLoS One. 2016 Feb 5;11(2):e0148243. doi: 10.1371/journal.pone.0148243 (PMC4743969; doi:10.1371/journal.pone.0148243)
Supplement: S21 File — (PDF) [file pone.0148243.s021.pdf]

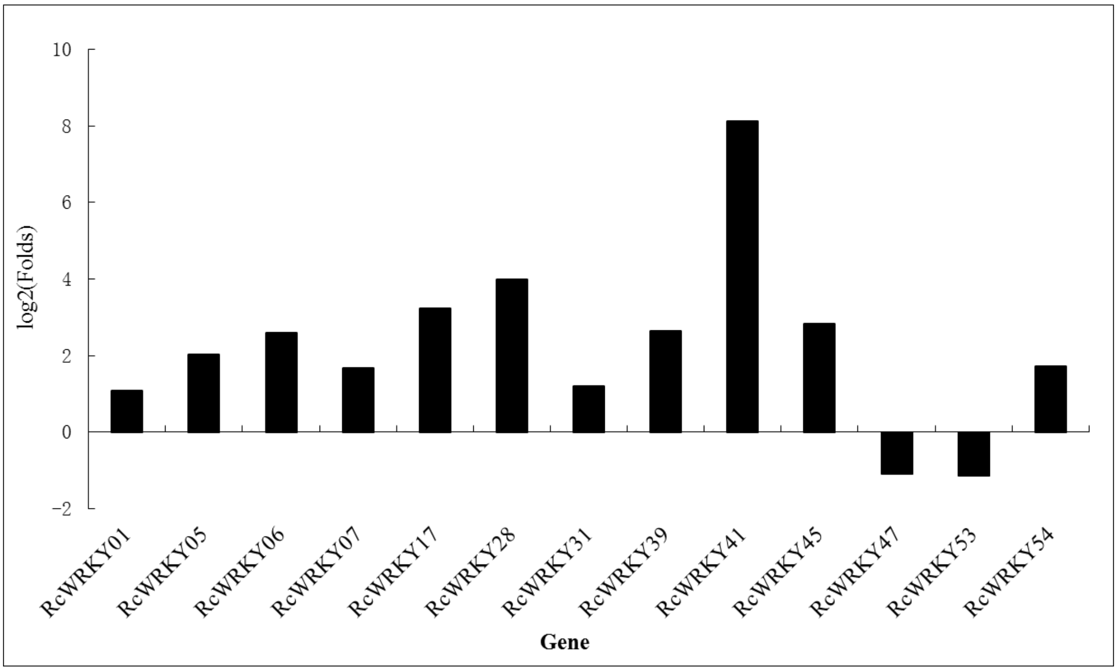

S21 File. List of 13 differentially expressed *RcWRKY* genes upon the ABA treatment. The y-axis represents the $\log_2$ transformed folds which were resulted from the application of 10 μM ABA for 24 h by using the *in vitro* developing seeds as reported by Chandrasekaran et al., (2014) [52].
